# Comparative Proteomic Analysis of Susceptible and Resistant Rice Plants during Early Infestation by Small Brown Planthopper

**DOI:** 10.3389/fpls.2017.01744

**Published:** 2017-10-17

**Authors:** Yan Dong, Xianping Fang, Yong Yang, Gang-Ping Xue, Xian Chen, Weilin Zhang, Xuming Wang, Chulang Yu, Jie Zhou, Qiong Mei, Wang Fang, Chengqi Yan, Jianping Chen

**Affiliations:** ^1^Agricultural Insect Laboratory, College of Plant Protection, Nanjing Agricultural University, Nanjing, China; ^2^State Key Laboratory Breeding Base for Zhejiang Sustainable Pest and Disease Control, Ministry of China Key Laboratory of Biotechnology in Plant Protection, Institute of Virology and Biotechnology, Zhejiang Academy of Agricultural Sciences, Hangzhou, China; ^3^Hunan Provincial Key Laboratory of Crop Germplasm Innovation and Utilization and Hunan Provincial Key Laboratory of Biology and Control of Plant Diseases and Insect Pests, Hunan Agricultural University, Changsha, China; ^4^CSIRO Agriculture and Food, St Lucia, QLD, Australia; ^5^Institute of Plant Protection, Jiangsu Academy of Agricultural Science, Nanjing, China; ^6^Plant Genetic Engineering Laboratory, College of Plant Protection, Zhejiang Normal University, Jinhua, China; ^7^Plant Pathogens Laboratory, College of Plant Protection, Shenyang Agricultural University, Shenyang, China; ^8^Institute of Biotechnology, Ningbo Academy of Agricultural Science, Ningbo, China

**Keywords:** rice, small brown planthopper, defense, proteomics, SBPH-susceptible, SBPH-resistant, differentially expressed protein

## Abstract

The small brown planthopper (*Laodelphax striatellus* Fallén, Homoptera, Delphacidae-SBPH) is one of the major destructive pests of rice (*Oryza sativa* L.). Understanding on how rice responds to SBPH infestation will contribute to developing strategies for SBPH control. However, the response of rice plant to SBPH is poorly understood. In this study, two contrasting rice genotypes, Pf9279-4 (SBPH-resistant) and 02428 (SBPH-susceptible), were used for comparative analysis of protein profiles in the leaf sheath of rice plants in responses to SBPH infestation. One hundred and thirty-two protein spots that were differentially expressed between the resistant and susceptible rice lines were identified with significant intensity differences (≥2-fold, *P* < 0.05) at 0, 6, and 12 h after SBPH infestation. Protein expression profile analysis in the leaf sheath of SBPH-resistant and SBPH-susceptible rice lines after SBPH infestation showed that proteins induced by SBPH feeding were involved mainly in stress response, photosynthesis, protein metabolic process, carbohydrate metabolic process, energy metabolism, cell wall-related proteins, amino acid metabolism and transcriptional regulation. Gene expression analysis of 24 differentially expressed proteins (DEPs) showed that more than 50% DEPs were positively correlated with their mRNA levels. Analysis of some physiological indexes mainly involved in the removal of oxygen reactive species showed that the levels of superoxide dismutase (SOD) and glutathione (GSH) were considerably higher in Pf9279-4 than 02428 during SBPH infestation. The catalase (CAT) activity and hydroxyl radical inhibition were lower in Pf9279-4 than 02428. Analysis of enzyme activities indicates that Pf9279-4 rice plants defend against SBPH through the activation of the pathway of the salicylic acid (SA)-dependent systemic acquired resistance. In conclusion, this study provides some insights into the molecular networks involved on cellular and physiological responses to SBPH infestation.

## Introduction

Rice (*Oryza sativa* L.) is a primary staple cereal (Tuyen et al., 2012) and is also a host species to many pests, which can significantly reduce rice yield. Among those insect pests, brown planthopper (BPH), white-backed planthopper (WBPH), and small brown planthopper (SBPH) are typical delphacidae planthoppers and major piercing-sucking pests of rice (Yang and Zhang, 2016). Like BPH and WBPH, the SBPH is a typical phloem-sucking insect and a widely spread pest during the main rice-growing areas in East Asia (Akira et al., 2010). SBPH causes significant yield losses by directly sucking the sap from rice phloem. The infected rice plants turn yellow and wilt and eventually die (Tuyen et al., 2012). In 2004 and 2005, 3.4 million hectares of rice were infected in Anhui and Jiangsu Provinces of China (Zhang et al., 2005). One generation of SBPH needs about 35–40 days and about 5–6 generations occur in Zhejiang and Jiangsu Provinces each year. SBPH also serves as a vector of many pathogens such as *rice black-streaked dwarf virus* (RBSDV) (Wang et al., 2010) and *rice stripe virus* (RSV) (Zhang et al., 2010; Wang et al., 2014). Use insecticide to control SBPHs is expensive, detrimental to natural predators and conducive to resistance built up in pests (Tanaka et al., 2000). In contrast to the chemical control, host-plant resistance is the most economical and effective approach to control SBPHs. Understanding on how rice responds to SBPH infestation will contribute to developing strategies for SBPH control. However, the lack of resistant sources to SBPH has potentially impeded efforts to understand how rice responds to SBPH infestation (Yang and Zhang, 2016).

Interactions between rice and planthoppers are complex. Gene expression profile studies have shown that genes involved in cell growth and photosynthesis are down-regulated, and genes involved in plant defenses and macromolecule degradation are up-regulated after BPH infestation (Wang et al., 2008), suggesting the alteration of C and N metabolism and a shift from growth and development to defense (Wang et al., 2008; Zhang et al., 2015). The key BPH defense include Ca^2+^ signaling, PR genes, MAPK cascades and receptor kinase, transcriptional regulation and protein post-translational modifications (Wang Y. et al., 2012; Lv et al., 2014). In general, rice plants defend against BPH through activation of the pathway of the SA-dependent systemic acquired resistance (Yang and Zhang, 2016). Proteins involved in SA biosynthesis are induced by BPH infestation (Wei et al., 2009). In response to WBPH attack, the defense proteins of thaumatin-, pathogenesis-, germin-related protein and α-amylase/trypsin inhibitor are up-regulated (Yang et al., 2014). Defense genes involved in secondary metabolism (i.e., multicopper oxidase protein, terpene synthase, agmatine coumaroyltransferase and anthranilate N-benzoyltransferase) are up-regulated in WBPH-resistant cultivar (Yang et al., 2014). In the interaction process between SBPH and rice, genes for TIFY protein, trypsin inhibitor genes and transcription factor genes are associated with SBPH resistance (Zheng et al., 2013). Fe homeostasis-related genes encoding enzymes that are involved in phytosiderophore biosynthesis, Fe transporters and regulators also display altered expression in rice after attack by SBPH (Zhang et al., 2017). The protein related to kinases, β-glucanases and oxidative stress response are differentially induced after BPH infestation (Wei et al., 2009). Comparing IR64 with its mutants, 22 proteins are associated with resistance to BPH (Sangha et al., 2013). The protein expression profile analysis in phloem sap between BPH-susceptible and BPH-resistant rice lines after BPH infestation has shown changes in the levels of proteins involved in redox regulation, signal transduction, protein and carbohydrate metabolic processes (Du et al., 2015). Proteomics is a useful tool to reveal physiological changes at the cellular level, but this technique has not been applied to the study of rice plant response to SBPH. In our previous studies, a wild species, *Oryza officinalis* (acc. HY018, *2n* = 24, CC), was found to have high resistance to SBPH (Yan et al., 2004; Zhang et al., 2014). Three QTLs of resistance to SBPH have been identified in Pf9279-4, an introgression line derived from *O. officinalis* and *O. sativa* (cv. 02428) by using asymmetric somatic hybridization, and are located on chromosome 3, 7, and 12 (Zhang et al., 2014). In this study, we analyzed the protein expression profile in rice leaf sheaths in response to SBPH infestation, aiming to explore the response of rice to SBPH infestation at protein level. We also measured some physiological indexes relevant to the pest resistant pathway such as SOD, GSH, CAT, glutamine synthetase (GS), peroxidase (POD), and hydroxyl radical inhibition.

## Materials and methods

### Plant materials

Two rice lines, Pf9279-4 (a SBPH-resistant introgression line derived from the asymmetric somatic hybridization between 02428 and *O. officinalis*) and 02428 (a SBPH-susceptible rice line), were selected in this study. Seeds of two rice lines (Pf9279-4 and 02428) were allowed to germinate in constant temperature incubator at 28°C. The seedlings were grown in greenhouse with 25 ± 1°C, 12 h photoperiod, and relative humidity of 60–80%.

### Insect materials

The SBPHs used for infestation in this study were derived from rice fields in Jiangsu Province of China, and maintained on rice variety Wuyujing 3 plants in Zhejiang Academy of Agricultural Sciences.

### SBPH infestation for proteomic analysis

At the third leaf stage, each seedling was infected with 15–20 SBPHs. Samples were derived from the outmost leaf sheath, as described by Liu et al. (2010), at 8 time points (0, 6, 12, 24, 36, 48, 72, and 96 h) after SBPH infestation. Three biological replicates were performed at each time point. Comparative analyses of protein levels were performed between the SBPH-resistant line Pf9279-4 and the SBPH-susceptible line 2428 (serving as a control) at each time point of SBPH infestation to identify differentially expressed proteins (DEPs).

### Rice plants growing for SBPH resistant analysis

Germinated seeds of Pf9279-4 and 02428 were planted in a plastic box in the insect-proof greenhouse. Seedlings at 40–60 days old, after stripping the old leaf sheaths and cutting off part of leaf and root, were washed with running water and cultivated in a nutrient solution for 4 days to ensure the seedlings alive.

A cultivated seedling of Pf9279-4 or 02428 was transferred to a tube (1.5 cm in diameter and 17 cm in height) containing the 2–3 cm depth of rice nutrient solution (Supplementary Figure S1A). The tubes were sealed by cotton. For the antixenosis experiment, the cultivated seedlings of Pf9279-4 and 02428 were transferred to the colorless glass cups (7 cm in diameter and 17 cm in height) containing 2–3 cm depth of rice nutrient solution (Supplementary Figure S1B). The glass cups were sealed by plastic wrap.

### Life span of adult SBPH

Each adult male or female SBPH at the start of eclosion was placed in the test materials (Pf9279-4 and 02428). The death date of adult SBPH was recorded to determine the life span of adult SBPH. One hundred biological replicates were performed for each rice line.

### Nymphal duration

Twenty-five nymphs (just hatched within 24 h) were placed on each rice seedling (serve as one replicate). The test was performed with 100 replicates for each rice line in the insect-proof greenhouse at 25 ± 1°C. The date of eclosion of SBPH was recorded.

### Number of eggs

A couple of male and female adult SBPHs (within 24 h of eclosion) were placed in the tube. The test was performed with 40 replicates for each rice line in the insect-proof greenhouse at 25 ± 1°C. The number of SBPH nymphs was recorded. The rice plants were dissected to find unhatched eggs under the stereoscope until there were no nymphs for 3 days. The numbers of nymphs and unhatched eggs were used to calculate the number of eggs and hatching rate.

### Survival rate

Forty SBPH nymphs (1st instar) (Duan, 2008; Duan et al., 2008) were placed on each seedling for 9 days. There were 40 replicates for each rice line. The number of SBPHs on each seedling was counted each day, starting 24 h after infestation. The average of survival rate on each seedling was calculated and treated as the antibiosis value.

### Value of antixenosis

Forty insects of the different stage of SBPH were placed in the glass cup. There were 40 replicates for each rice line. The number of SBPHs on each seedling was counted each day for 9 days, starting 24 h after infestation, and then dispersed evenly among the seedlings after counting every day. The average value of antixenosis on each seedling was the ratio of number of SBPHs on each rice plant to total number of SBPHs after 9 days scoring.

Statistical significance of the data was determined using SPSS Statistics ver. 22.0. Significant differences of one-way ANOVA were determined by the LSD test at *P* = 0.05 or *P* = 0.01. Analysis of variance was carried out using Excel ver. 2007.

### Extraction of total proteins for two-dimensional fluorescence difference gel electrophoresis (2D-DIGE)

At the selected time point after SBPH infestation, three biological replicates of the outmost layer of leaf sheaths were sampled from 02428 and Pf9279-4 rice plants, respectively. About 1.5 g of leaf sheaths were used to extract the protein. The leaf sheath proteins were extracted using a modified phenol-methanol method as described previously (Deng et al., 2007). For each sample, rice leaf sheath tissues were ground into a powder with a pestle and mortar in liquid nitrogen. About 1.5 g of tissue powder was transferred to a 50-mL centrifuge tube and then mixed with 5 mL of SDS extraction buffer (1% β-mercaptoethanol, 10 mM EDTA, 100 mM Tris-HCl, 2% SDS, 5 mM EGTA and pH 8.0). After heating at 65°C for 15 min, the mixture was centrifuged at 20,000 g and 25°C for 25 min. The supernatant was transferred to a new 50-mL centrifuge tube, was mixed with 5 ml of ice-cold Tris-saturated phenol (Tris-buffered, pH 7.5–7.9) and stood at room temperature for about 10 min. The mixture was centrifuged at 20,000 g and 4°C for 15 min and the supernatant was removed. The phenol phase was extracted twice with 7 ml of 50 mM Tris-HCl (pH 8.0) for 10 min and then centrifuged at 20,000 g and 4°C for 15 min. The supernatant was removed and the under phase was mixed with 25 ml of cold 0.1 M ammonium acetate-methanol solution overnight at 20°C to precipitate proteins. After centrifugation at 20,000 g and 4°C for 15 min, the supernatant was discarded and the precipitation was washed once with 25 mL of 0.1 M ammonium acetate methanol solution. After standing at −20°C for about 20 min, the mixture was centrifuged at 20,000 g and 4°C for 15 min and the protein pellet was washed twice with 600 μL of ethanol. After centrifugation at 20,000 g and 4°C for 15 min, the protein pellet was dried at room temperature for about 10 min. The dried powder was dissolved in a lysis buffer solution (7 M urea, 65 mM Tris-HCl buffer pH 6.8, 2 M thiourea, 0.2% IPG buffer containing ampholyte pH 4-7, and 4% CHAPS) and heated at 30°C for 1 h. After centrifugation at 20,000 g and 4°C for 15 min, the supernatant was transferred to 1.5 mL centrifuge tube. The purification of proteins was performed using 2-D Clean-Up Kit (GE Healthcare, UK). The concentration of proteins was measured using a 2-D Quant kit (GE Healthcare, UK) according the manufacturer's instructions and then adjusted to 5 μg/μl.

### Protein cydye labeling and 2D-DIGE

Proteins were labeled with CyDyes DIGE Fluors based on manufacture's instructions (GE Healthcare) as shown in Supplementary Figure S2.

A 50 μg protein sample was mixed with 400 pmol CyDye (pH 8.5) and the mixture was incubated on ice for 30 min in the dark. The reaction was terminated by adding 1 μl of 10 mmol/L lysine and incubating on ice for 10 min. Protein samples labeled reciprocally with Cy3 and Cy5 as well as the Cy2-internal standard according to the manufacturer's instructions (GE Healthcare) were mixed together in one tube. The randomization was done to negate gel to gel variations as shown in Supplementary Figure S2. The volume was adjusted to 150 μL by adding 2 × sample buffer.

IPG strips were rehydrated at 20°C for 12 h. The samples were added into 24-cm IPG strips (GE Healthcare). Ettan IPGphor II (GE Healthcare) was used in IEF with the following setting: 500 V for 1 h, 1,000 V for 3 h, gradually increasing to 8000 V in 4 h, and maintaining at 8000 V until reaching the desired total V-h (70,000 for IPG strips pH 4–7). After IEF, IPG strips were equilibrated twice (15 min each) in equilibration buffer (30% glycerol, 6 M urea, 0.01% bromophenol blue, 2% SDS, 50 mM Tris-HCl buffer, pH 8.8) containing 2% DTT (the first equilibration) or 2.5% iodoacetamide (the second equilibration). The IPG strips were transferred to SDS-PAGE gels (12.5%) using the Ettan Dalt Twelve gel system (GE Healthcare) for the second dimension electrophoresis. SDS-PAGE was run until the bromphenol blue dye front reached the gel end.

### Image scan and data analysis

The gel was scanned using a Typhoon 8600 scanner (GE Healthcare). Cy2 image was scanned using a 480/30 nm laser and a 530/40 nm band-pass emission filter. Cy3 image was scanned using a 540/25 nm laser and a 595/25 nm band-pass emission filter. Cy5 image was scanned using a 635/30 nm laser and a 680/30 nm band-pass emission filter. The gel was scanned at 100 μm (pixel size) and the maximum pixel intensity was between 30,000 and 55,000. Image analysis was accomplished using DeCyder 2D 7.0 (GE Healthcare, UK). PDQuest software was used in Quantitative intensity analysis. The gel of 02428 samples was selected as a reference gel (control) and the gel of Pf9279-4 samples was compared with the reference gel at each time point (0, 6, and 12 h after infestation) to determine differential expression protein (DEP) spots. The data analysis included spot detection, background subtraction and normalization. Spot detection was performed by the DIA (differential in-gel analysis) module. After removing the artifact spots by manual editing, the image was analyzed by the DeCyder BVA (biological variation analysis) module. After automated detection and matching, manual editing was carried out. Every pairwise comparison was made within a single 2D DIGE gel. The limit was set to 1.5, 2.0, and 3.0 fold change, respectively by Student's *t*-test (*P* < 0.05). Then, the Boolean analysis sets were created between the statistic sets and the quantitative or qualitative sets. The spots were compared among three replicates. Only spot displaying reproducible change patterns was considered to be a DEP.

### Two-dimensional gel electrophoresis (2-DE), MS analysis and database searching

For 2-DE, 500 μg protein samples without CyDye were added into IPG strips for spot picking and second-dimension electrophoresis was performed as mentioned above. Gel was stained by a staining solution (20% (v/v) alcohol, 0.12% (v/v) coomassie brilliant blue (CBB) G-250, 10% (w/v) ammonium sulfate and 10% (v/v) phosphoric acid) and destained in double-distilled H_2_O (Wang X. et al., 2012). The 2-DE image was scanned by the UMAX Power Look 2100XL scanner (Maximum Tech, Taiwan, China) at 300 dpi. These images were compared with the 2D-DIGE images to identify spots of interest. Protein spots were manually excised from the gels and cut into small pieces.

Spots of interest were digested according to the method of Yan (Yan et al., 2005). The digested protein samples were subjected to analyses of MS and MS/MS data using a 4800 Plus MALDI TOF/TOF™ Analyzer (Applied Biosystems, CA, USA). Monoisotopic peak masses were automatically determined within the mass range 800–4,000 Da with a minimum signal-to-noise ratio (S/N) of 10 and a local noise window width of m/z 250. Five of the most intense ions with S/N 450 were selected as precursors for MS/MS acquisition, excluding common trypsin autolysis peaks and matrix ion signals. The MS together with MS/MS spectra were searched using the software GPS Explorer (version 3.6, Applied Biosystems) and MASCOT (version 2.1, Matrix Science) against the uniprot-Oryza-sativa database (released on Dec 12, 2016, 168 307 rice protein sequences) (Gu et al., 2016). The other parameters for searching were enzyme (trypsin cleavage, one missed cleavage allowed), fixed modifications [carbamidomethylation (Cys)], variable modifications [oxidation (Met)], peptide mass tolerance (100 ppm) and fragment tolerance (0.3 Da). The minimum ion score confidence interval (C.I.) for MS/MS data was set to 95%. Only the proteins with C.I. > 95% were considered to be positive identification.

### Protein classification analysis

The function of the identified proteins was identified based on UniProt database (http://www.ebi.uniprot.org).

### Quantification of gene expression by quantitative real-time PCR (qPCR)

Total RNA was extracted from the outmost layer of leaf sheath powder at 0, 6, and 12 h after SBPH infestation using Trizol based on the supplier's instructions. Residual DNA was removed by RNase-free DNase. Total RNA was reverse-transcribed by the method of reverse transcriptase and oligo (dT) (iScript cDNA synthesis Kit, BIO-RAD). Quantitative PCR was performed using Kangwei SYBR Green Supermix with Light cycler 480 (Roche). The PCR conditions were as follows: 45 cycles, 95°C for 30 s, 55°C for 45 s, and 72°C for 30 s. To minimize sample variation, the housekeeping gene Actin-122 was used as an internal control. Three technical repeats were performed for each sample. The comparative threshold cycle (Ct) was used for the quantification of mRNA (Livak and Schmittgen, 2001).

The primers were designed using Primer 5. All primers were listed in Supplementary Table S1. Intra-assay variation was evaluated by calculating SD errors of arithmetic means of three replicates.

### Physiological index

Rice materials were extracted from the outmost layer of rice leaf sheath at 8 time points (0, 6, 12, 24, 36, 48, 72, and 96 h after SBPH infestation) in 3-week-old rice seedlings. The activities of SOD, GSH, CAT, GS, POD, and hydroxyl radical inhibition were measured by using the method of Giannopolitis and Ries (1977a,b).

SPSS Statistics ver. 22.0 was used to determine the Statistical significance of the data. One-way ANOVA was performed and the LSD test at *P* = 0.05 or *P* = 0.01 was used to identify significant differences. Analysis of variance was carried out using Excel ver. 2007.

## Results

### Physiological responses induced by SBPH infestation in 02428 and Pf9279-4

**Figure 1:**
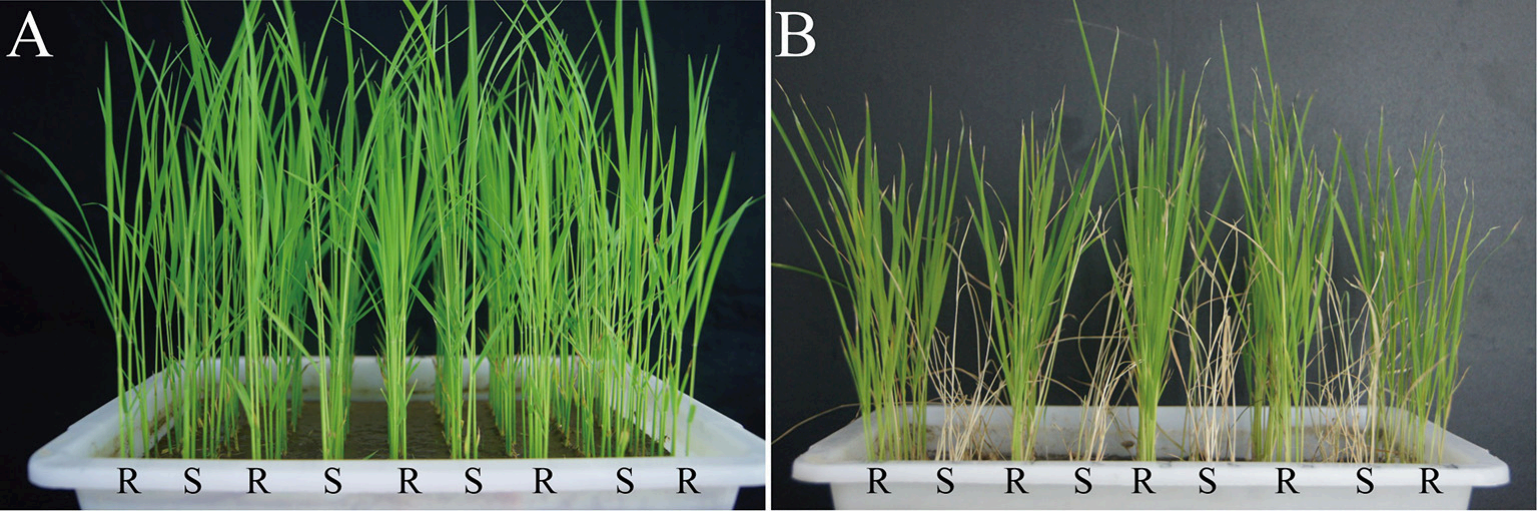
Changes in morphology of two rice lines (Pf9279-4 and 02428) after SBPH infestation. **(A)** The morphology of the two rice lines without SBPH infestation. **(B)** The morphology of the two rice lines at the eighth day after SBPH infestation. Each plant was treated with 20 SBPHs. “R” represents the SBPH-resistant line Pf9279-4 and “S” represents the SBPH-susceptible line 02428.

In our previous study one wild species (*O. officinalis*) showed high resistance (HR) to SBPH and 02428 was highly susceptible (HS) to SBPH. The resistance scores of *O. officinalis* and 02428 were 0.0 and 8.8, respectively (Supplementary Table S2; Zhang et al., 2014). Pf9279-4 is an introgression line derived from the asymmetric somatic hybridization between *O. officinalis* and 02428. Pf9279-4 had an average resistance score of 2.8 in the seedling bulk test (Supplementary Table S2; Zhang et al., 2014). Figure 1 shows morphology result between 02428 and Pf9279-4 at the eighth day after SBPH infestation. 02428 and Pf9279-4 were susceptible and resistant to SBPH at the seedling stage, respectively.

### Resistant type of Pf9279-4 and 02428 to SBPH

#### Life span of adult SBPH

**Figure 2:**
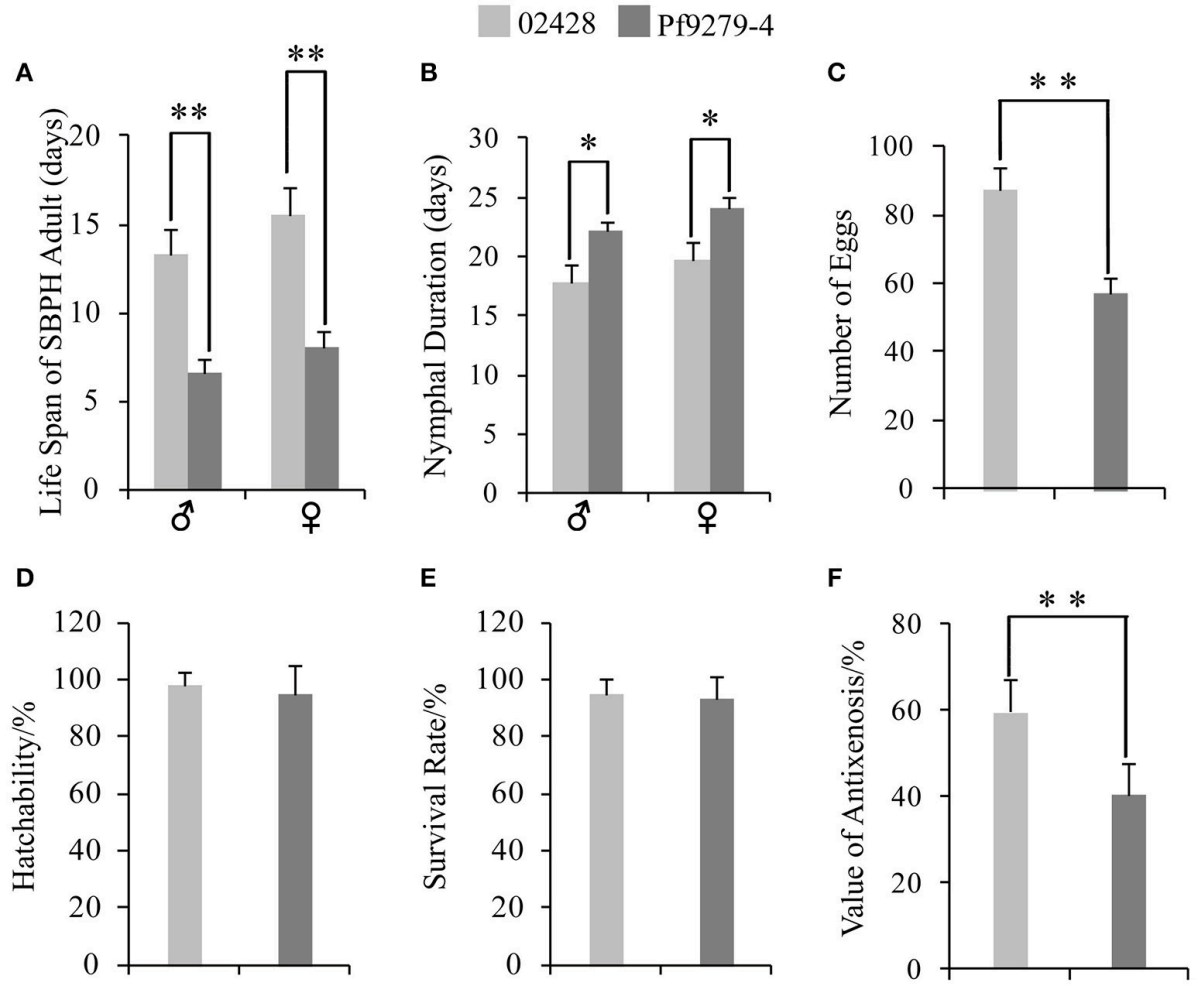
Resistant type of Pf9279-4 and 02428 to SBPH**. (A)** Life span of adult SBPH. **(B)** Nymphal duration. **(C)** Number of eggs. **(D)** Hatchability. **(E)** Survival rate. **(F)** Value of antixenosis. One-way ANOVA was used to determine statistical differences. ^*^P < 0.05, ^**^P < 0.01.

The life span of adult male SBPH settled on Pf9279-4 was 6.55 d, significantly shorter than settled on 02428 rice plants (13.30 d) (Figure 2A). Similarly, the life span of adult female SBPH settled on Pf9279-4 was shorter than on 02428 rice plants (Figure 2A).

#### Nymphal duration

The nymphal duration of male SBPH settled on Pf9279-4 was significantly longer than settled on 02428 rice plants (22.10 d for Pf9279-4 and 17.87 d for 02428; Figure 2B). The nymphal duration of female SBPH settled on Pf9279-4 was also longer than on 02428 (Figure 2B).

#### Number of eggs

The numbers of eggs of SBPH fed on Pf9279-4 and on 02428 were 57.07 and 87.05, respectively. SBPH fed on Pf9279-4 plants produced significantly less eggs than on 02428 (Figure 2C).

#### Hatchability

The egg hatchability of SBPH fed on both Pf9279-4 and 02428 was very high (94.95% and 97.84%, respectively). There was no significant difference between these two rice lines (Figure 2D).

#### Survival rate

The average values of SBPH survival rate after 15 days of infestation on Pf9279-4 and on 02428 were 92.92 and 95.19%, respectively. Although the survival rate on Pf9279-4 was lower than on the 02428, there was no significant difference between the two rice lines (Figure 2E). The result suggests that Pf9279-4 resistance to SBPH is not antibiosis.

#### Value of antixenosis

As shown in Figure 2F, the value of antixenosis on Pf9279-4 was significantly lower than on 02428 (40.42% for Pf9279-4 and 59.58% for 02428). Thus, we speculate that antixenosis contributes to Pf9279-4 resistance to SBPH.

### 2D-DIGE analysis of total proteins in rice leaf sheaths after SBPH infestation and mass spectrometry

To eliminate potential viral infection, the virus (RSV and RBSDV) detection was performed in all the samples. As shown in Supplementary Figure S7, all the samples were found to have no viruses.

To investigate the temporal changes of protein profiles at 0 h, 6, 12, 24, 36, 48, 72, and 96 h after SBPH infestation between Pf9279-4 and 02428, we carried out 2D-DIGE analysis of the total proteins of leaf sheaths from three biological replicates. The comparisons of DEPs were performed between susceptible and resistant rice lines before and after SBPH infestation. At each time point, 02428 was used as a control for Pf9279-4. For each sample, triplicate gels were performed, and they showed a high level of reproducibility.

Three cut-off values of 1.5-, 2.0-, and 3.0-fold change were independently adopted to ascertain the expression levels of proteins that were differentially altered between 02428 and Pf9279-4. The protein spot was considered as a really differentially expressed protein spot if the alteration of its expression was consistent in triplicate 2D-DIGE gels. The number of DEPs among the selected time points at the different cut-off value is shown in the Supplementary Figure S3. At the cut-off value of 1.5-fold, two peaks in term of the DEP number were at 6 h and 24 h. At the cut-off value of 2- and 3-fold, two peaks were at 6 h and 36 h. We chose protein spots at the time of either side of the peak and the peak point for research.

In the previous study, there was much work on rice plants during later SBPH infestation. However, the comparative proteomics between SBPH-resistant and SBPH-susceptible rice lines during early SBPH infestation is few, which will be very informative in studying rice-insect interaction. So DEPs at 0, 6, and 12 h were the focus in this study.

The 2D-DIGE images are shown Supplementary Figures S4–S6 with the triplicate gels at 0, 6, and 12 h after SBPH infestation. 2D-DIGE image analysis showed 166 protein spots with changes in their expression levels by more than 2.0-fold (*P* < 0.05) between 02428 and Pf9279-4 at three time points (0, 6, and 12 h). A total of 132 DEP spots was identified by MS based on uniprot_Oryza_sativa database (Additional file: Data sheets 1–4) at 0, 6, and 12 h after SBPH infestation. As shown in Data sheets 2–4, matched peptides were either the primary level—PMF (protein score C.I.%) or the secondary level—ion match peptides (total ion score C.I.%). The minimum ion score confidence interval (C.I.) for MS/MS data was set to 95%. Only the proteins with protein score C.I.% > 95% were considered to be positive identification. The proteins with their total ion score C.I. > 95% have been considered to be more positive identification. We selected the protein spots with ion score C.I. > 95%. Single peptide data has been marked with symbol “+” in Supplementary Table S3, and its spectra have been added in Supplementary Figure S8.

As shown in Supplementary Table S3, 132 DEP spots were annotated either as hypothetical proteins or as proteins with specific function in the database. The isoelectric point (*pI*) of the protein spots range from 4.76 to 9.64, and the molecular masses (Mr) range from 15.46 to 93.38 kDa. Some spots had same *pI* and Mr derived from different positions were the same name. But these spots may be the different products due to alternative splicing, post-translational modifications of protein, proteolytic cleavage or nucleotide polymorphisms (Schlüter et al., 2009).

**Figure 3:**
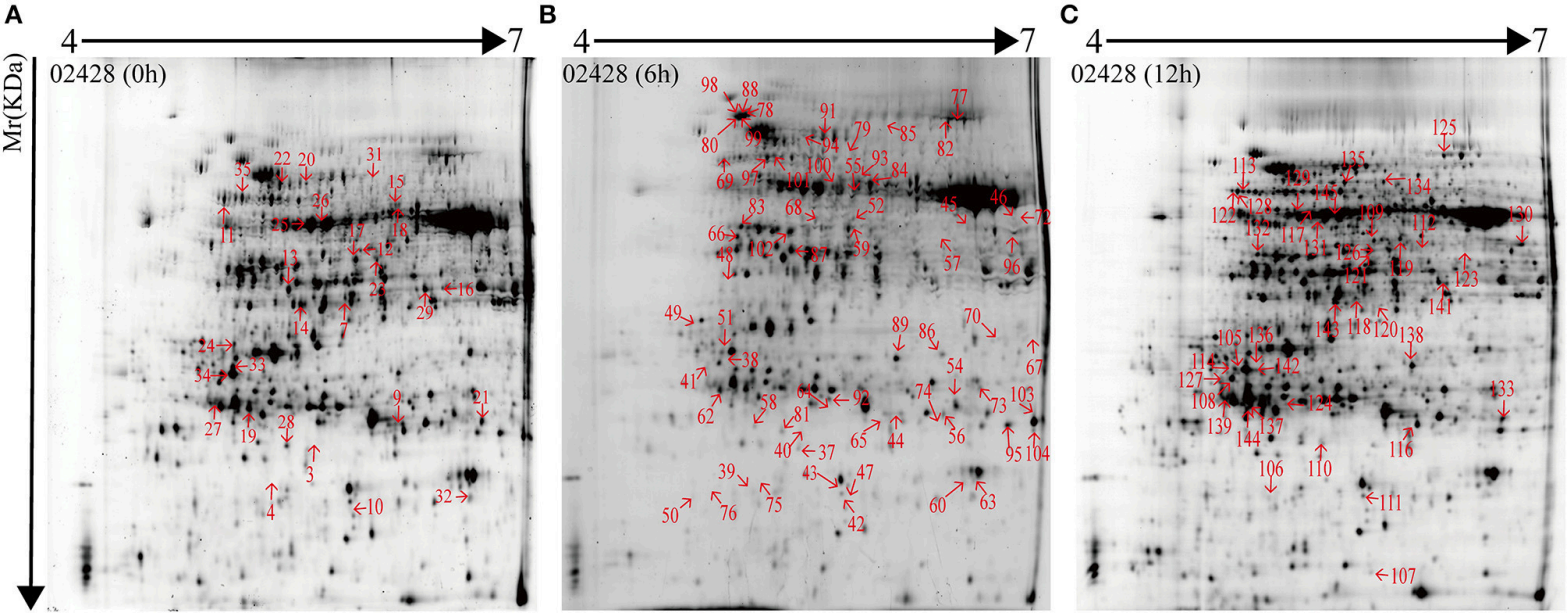
2-DE maps of proteins extracted from the leaf sheath proteins of 02428. Quantitative image analysis revealed that a total of 132 protein spots changed their intensities significantly (*P* < 0.05) by more than 2.0-fold at 0 h **(A)**, 6 h **(B)**, and 12 h **(C)** post SBPH infestation in 02428 in comparison with Pf9279-4.

**Figure 4:**
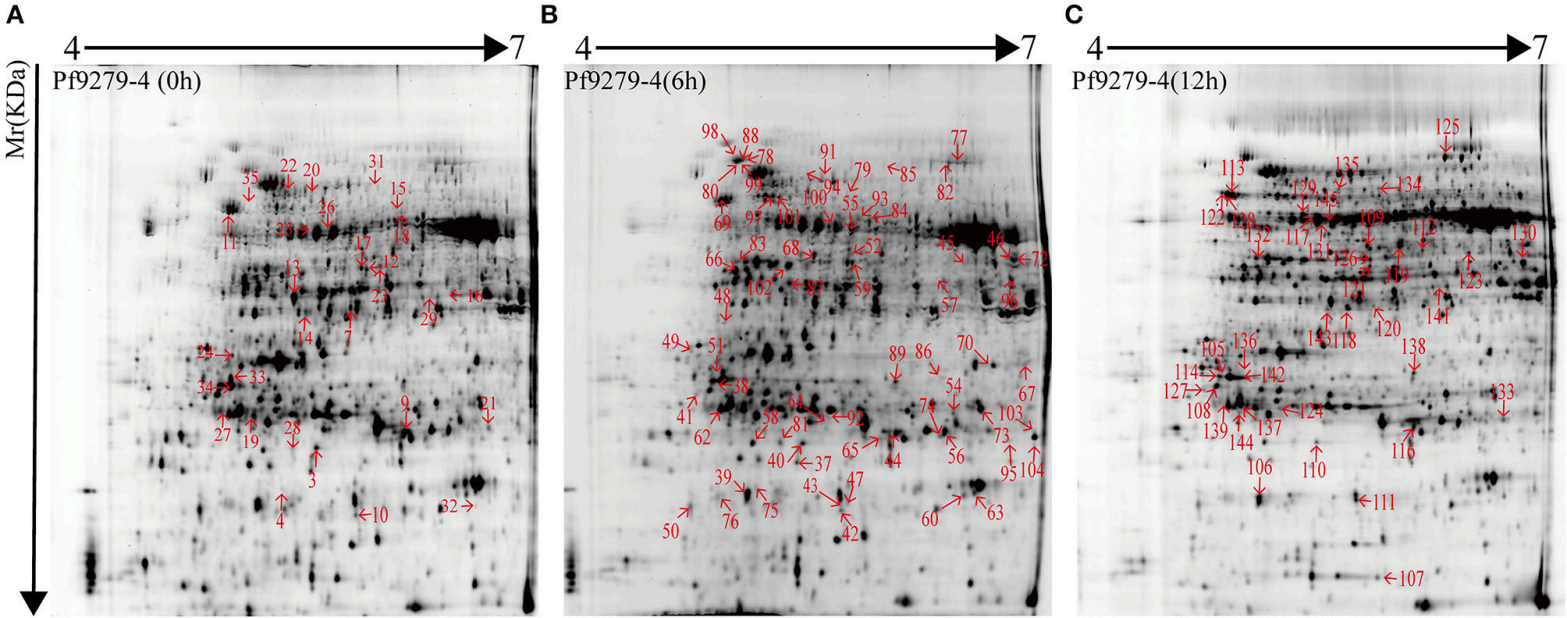
2-DE maps of proteins extracted from the leaf sheath proteins of Pf9279-4. Quantitative image analysis revealed that a total of 132 protein spots changed their intensities significantly (*P* < 0.05) by more than 2.0-fold at 0 h **(A)**, 6 h **(B)**, and 12 h **(C)** post SBPH infestation in Pf9279-4 in comparison with 02428.

132 DEP spots were both marked in gels of 02428 and Pf9279-4 (Figures 3, 4). The same differentially expressed protein spot in different gels was magnified and 3D view of DEP derived from resistant- and susceptible-lines illustrates differential expression (Supplementary Figure S9).

**Figure 5:**
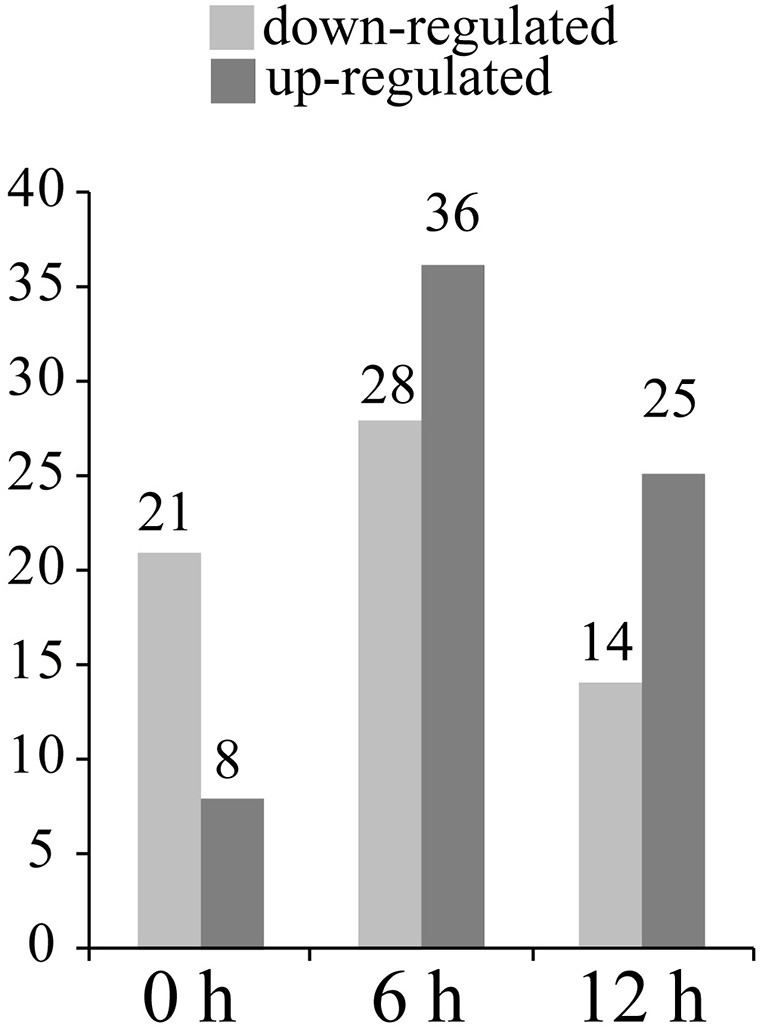
The number of differentially expressed protein spots at three time points. The number of the up- and down-regulated DEPs in Pf9279-4 at 0, 6, and 12 h post SBPH infestation (≥ 2-fold, *P* < 0.05) in comparison with 02428.

29 protein spots changed their intensities in Pf9279-4 compared with 02428 without SBPH infestation (i.e., at 0 h). 21 of them were down-regulated in Pf9279-4 and 8 of them were up-regulated. 64 DEP spots were found at 6 h after SBPH infestation with 28 of them being down-regulated and 36 of them up-regulated. 39 DEP spots were identified at 12 h after SBPH infestation with 15 of them being down-regulated and 24 of them up-regulated (Figure 5).

### Function classification of SBPH-responsive proteins

**Figure 6:**
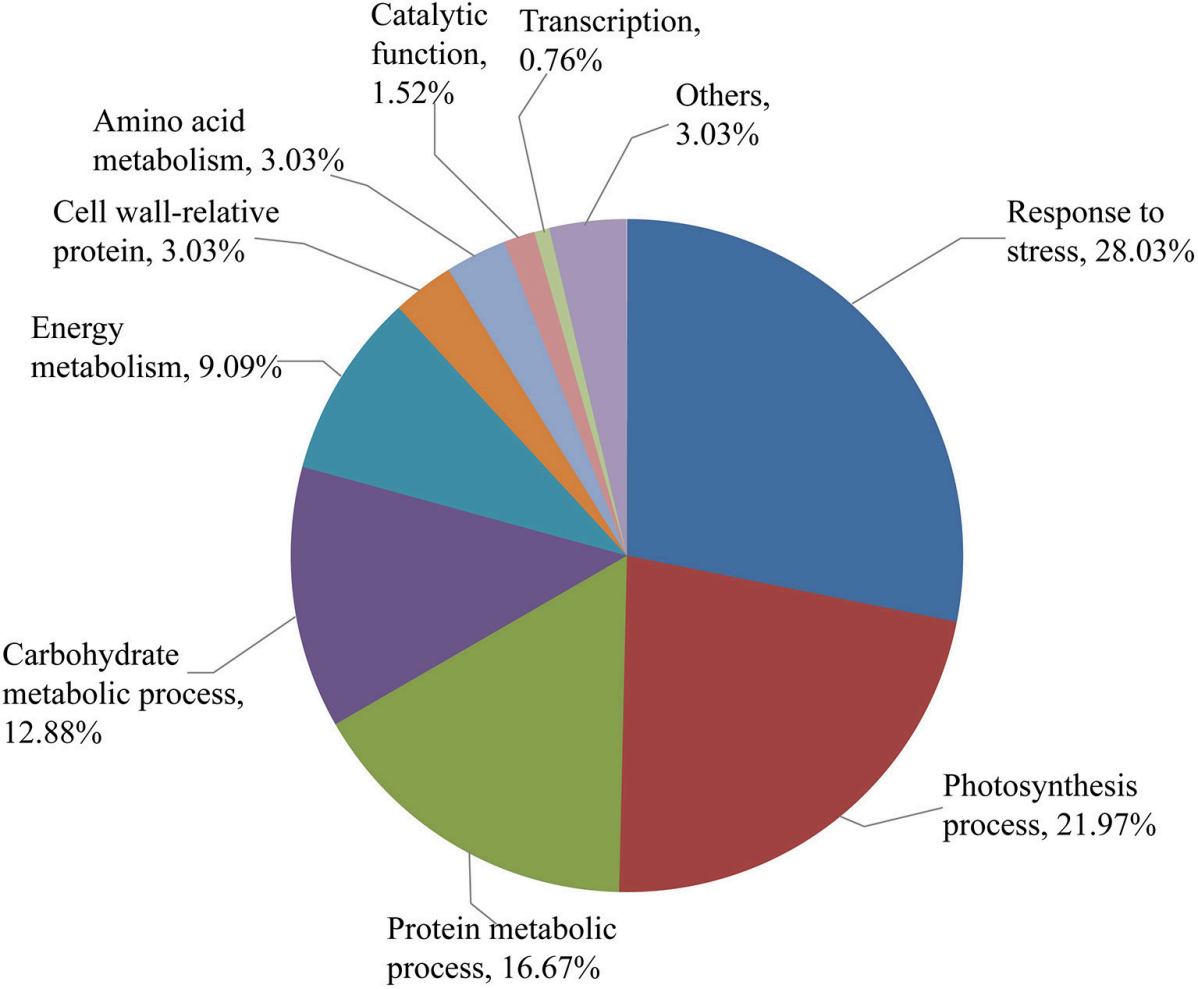
Functional categorization of the identified protein spots. Identified protein spots are grouped by their biological processes.

The 132 DEP spots were grouped according to their biological processes using the GO annotation. As shown in Figure 6, these DEPs were classified into stress response (28.03%), photosynthesis process (21.97%), protein metabolic process (16.67%), carbohydrate metabolic process (12.88%), energy metabolism (9.09%), cell wall-related protein (3.03%), amino acid metabolism (3.03%), catalytic function (1.52%), transcription (0.76%), and others (3.03%). The largest functional category was stress-responsive proteins, which were greatly affected by SBPH infestation.

### Correlation analysis between mRNA and protein expression by qPCR

**Figure 7:**
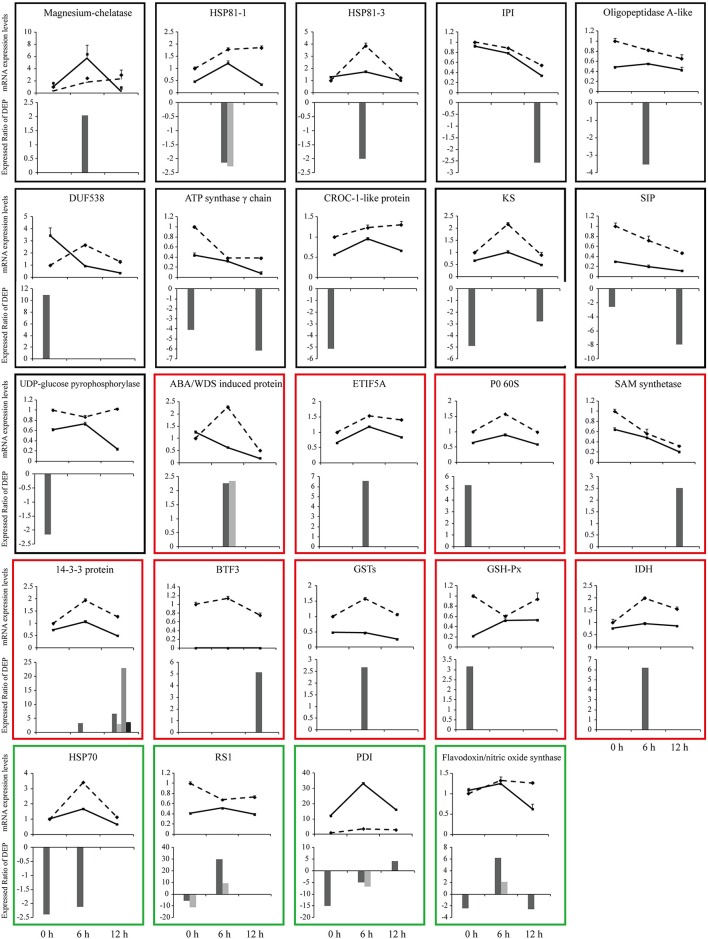
qPCR analysis of gene expression in leaf sheath at three time points (0, 6, and 12 h after SBPH infestation). Relative mRNA levels of some differentially expressed proteins (shown in Supplementary Table S3) were determined by qPCR analysis. Values are means ± standard error (*n* = 3). Solid line shows the relative expression levels of Pf9279-4 and dotted line shows the relative expression levels of 02428. Bar charts show expression ratio of DEPs at each time point. In some cases the same protein has more than two spots. The black frame shows that the mRNA levels are highly correlated with their protein levels. The red frame represents that the mRNA level shows a low correlation with their protein levels. The green frame shows that the mRNA levels have a either high or low correlation with their protein levels during the course of SBPH infestation.

To investigate the changes of DEPs at mRNA level, we performed qPCR analysis. The mRNA levels of 24 DEPs were analyzed at 0 h, 6 h and 12 h after SBPH infestation. Figure 7 shows an intuitive overview of the correlation between mRNA and protein levels. About 50% DEPs were highly correlated with the mRNA levels. The mRNA levels of 11 of the 24 DEPs [magnesium-chelatase (Spot 66), HSP81-1 (Spot 78 and 99), HSP81-3 (Spot 98), IPI (Spot 136), oligopeptidase A-like (Spot 94), DUF538 (Spot 3), ATP synthase γ chain (Spot 29 and 141), CROC-1-like protein (Spot 32), KS (Spot 31 and 134), SIP (Spot 24 and 142) and UDP-glucose pyrophosphorylase (Spot 17)] were highly correlated with their changes in their protein levels. The mRNA levels of 8 DEPs [ABA/WDS induced protein (Spot 60 and 63), ETIF5A (Spot 42), P0 60S (Spot 7), SAM synthetase (Spot 119), G-box binding factor, 14-3-3 protein (Spot 105, 108, 114, and 127), BTF3 (Spot 110), GSTs (Spot 54), GSH-Px (Spot 10) and IDH (Spot 45)] showed a low correlation with their protein levels. The mRNA levels of 4 DEPs [HSP70 (Spot 20 and 78), RS1 (Spots 33, 34, 38, 41), PDI (Spots 35, 97, 101, 113) and flavodoxin/nitric oxide synthase (Spots 21, 44, 74, and 113)] changed their abundance at least at two time points and were either high or low correlation with their protein levels.

### Physiological indexes analysis

**Figure 8:**
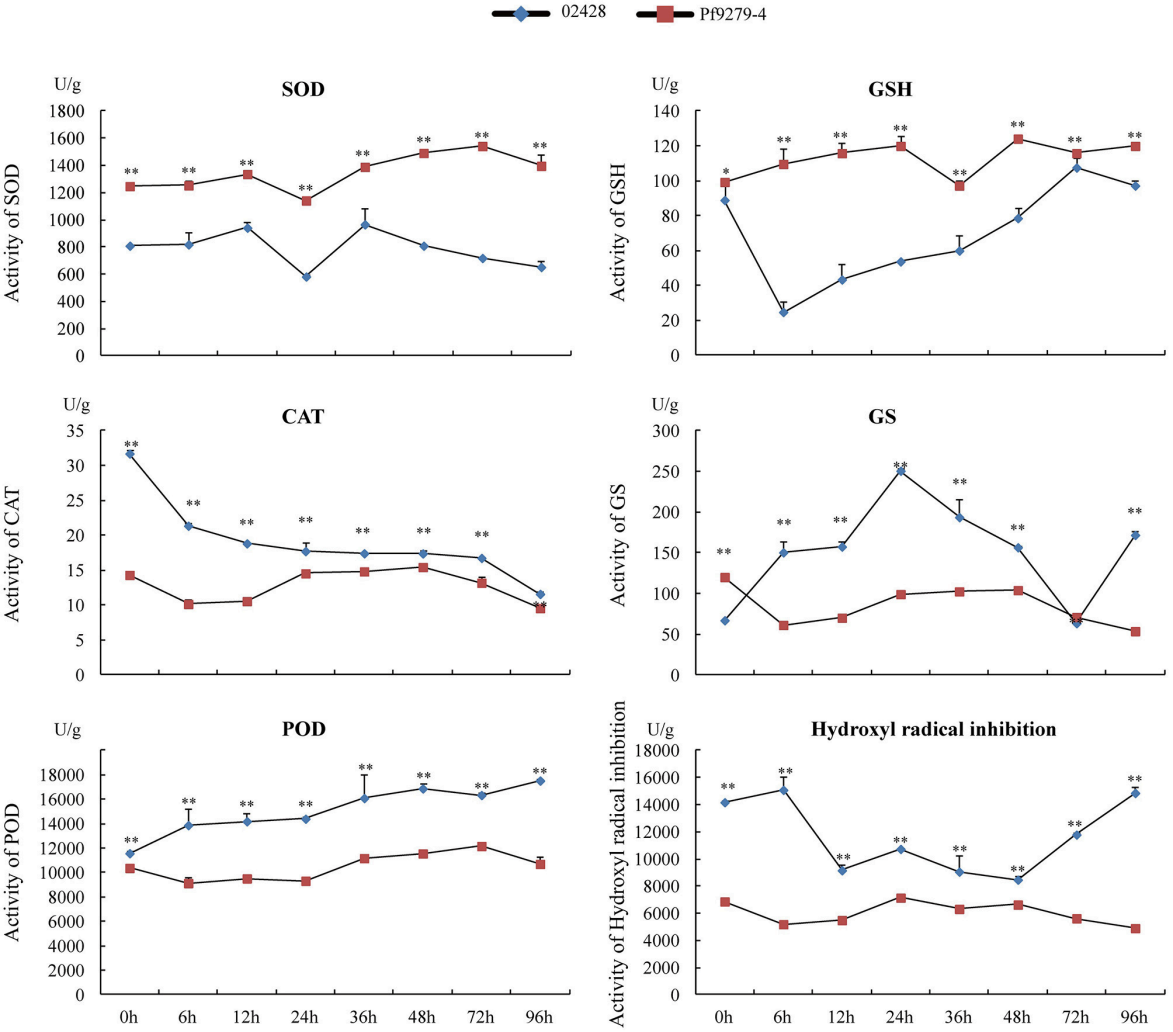
Activities of SOD, GSH, CAT, GS, POD, and hydroxyl radical inhibition in the leaf sheath of Pf9279-4 and 02428 before and after SBPH infestation. The leaf sheath of Pf9279-4 and 02428 were extracted at 0, 6, 12, 24, 36, 48, 72, and 96 h after SBPH infestation.

It is known that reactive oxygen species (ROS) produced under stress conditions can cause damage to cellular components. Plants can control the ROS level through scavenging them by antioxidants. The biochemical changes caused by SBPH infestation were investigated by measuring the activities of SOD, GSH, CAT, GS, POD, and hydroxyl radical inhibition (Figure 8) at 8 time points between resistant and susceptible rice lines.

Under the stress plants produce a large number of ·${\text{O}}_{2}^{-}$, which has extremely strong oxidation ability and is one of the major factors of ROS poisoning. Superoxide dismutase (SOD) can eliminate ·${\text{O}}_{2}^{-}$. SOD activity in Pf9279-4 was significantly higher than that in 02428 at each time point (*P* < 0.01). The activity of SOD slightly increased in Pf9279-4 after SBPH infestation, but not in 02428.

Glutathione (GSH) is one of the important antioxidants in plants and a free radical scavenger. GSH activity in Pf9279-4 was also significantly higher than that in 02428 at each time point (*P* < 0.05). The activity of GSH was slightly lower in 02428 at the 0 h time point than in Pf9279-4 and decreased markedly following 6-h SBPH infestation. In contrast, the activity of GSH slowly increased over time in Pf9279-4 after SBPH infestation.

Catalase (CAT) hydrolyzes hydrogen peroxide into water and oxygen. The CAT levels were markedly higher in 02428 than in Pf9279-4 at the 0 h time point and gradually decreased over time. In Pf9279-4 the CAT levels was relatively stable during SBPH infestation. The comparative analysis of the CAT activity between the resistant and susceptible lines showed the extremely significant difference (*P* < 0.01) at all the time points and the differences in the CAT activity between the resistant and susceptible rice lines were bigger during early SBPH infestation.

Peroxidase (POD) is associated with the processes of respiration, photosynthesis and the oxidation of auxin. POD is more active in aging tissues. POD is also involved in lignin synthesis, increasing the degree of lignification. The activity of POD slightly increased over time in 02428, but not in Pf9279-4. The comparison analysis of POD activity between the resistant and susceptible rice lines showed markedly significant differences (*P* < 0.01) at all the time points. The differences in POD activity of between the resistant and susceptible lines became bigger following SBPH infestation.

Glutamine synthetase (GS) converts glutamic acid and ammonium ion, which is harmful to tissue, into glutamine. The GS level in 02428 showed a peak at 24 h and a valley at 72 h post SBPH infestation. The activity of GS in Pf9279-4 had no obvious changes over time of SBPH infestation. The activity of GS in Pf9279-4 was higher than in 02428 at 0 h and 72 h after SBPH infestation. At 96 h, the activity of GS in 02428 was higher than in Pf9279-4.

Hydroxyl radical is a kind of ROS. ROS produced under stress conditions can function as a signaling molecule for stress responses. But ROS can also cause damage to cellular components. The hydroxyl radical inhibition levels in 02428 slightly increased at 6 h, but reduced afterwards and were recovered at 48 h after SBPH infestation. The levels of hydroxyl radical inhibition was stable in Pf9279-4 over time. The activity of hydroxyl radical inhibition in 02428 were higher than that in Pf9279-4 at all the time points (*P* < 0.01).

## Discussion

### Mechanism of antixenosis in resistant rice plant Pf9279-4 to SBPH

This study showed that the fecundity of female adult SBPH settled on the Pf9279-4 was much lower than on the 02428, which resulted in low numbers of SBPH at the whole growth period of Pf9279-4. The nymphal durations of SBPH on Pf9279-4 were longer than on 02428, resulting in less generations of SBPH. The life spans of adult males and females were shorter on Pf9279-4 than on 02428, which led to the less damage of Pf9279-4 rice plants. These data clearly validated the usefulness of Pf9279-4 in SBPH control. The resistant type of Pf9279-4 to SBPH was antixenosis, which reduced the SBPH settling on the rice plants and significantly suppressed the feeding.

### Regulatory and functional DEPS govern molecular response in SBPH-resistant and susceptible rice plants

The degree of tissue damage and the mode of feeding lead to different molecular responses of plants to herbivores (Du et al., 2009). Little tissue damage can be produced by sucking insects. It has now been recognized that plant immunity to piercing-sucking insects resembles that in resistance to pathogens (Cheng et al., 2013). However, as insects are more sophisticated than pathogens, the interaction between plant and insect is more intricate at protein level. The DEPs identified in this study can be classified into regulatory and functional proteins.

Rice plants might perceive SBPH infestation stress signals by some receptors and sensors and transmit them to the cellular machinery by signal transduction to regulate gene expression. Some regulatory DEPs that were higher in the SBPH-resistant rice plant after SBPH infestation than the susceptible one are potentially involved in signal transduction, i.e., mannose-binding lectin protein, MBL, Ricin B-related lectin domain containing protein, RBRL and 14-3-3 protein. MBL and RBRL are the host defense proteins and can specifically recognize all kinds of carbohydrates on the surface of pathogens, which subsequently activate intracellular signal pathways (Mandal et al., 2015). The 14-3-3 protein plays a role in modulating the biosynthesis of some metabolic enzymes, vesicle shuttle, cell cycle, apoptosis and especially cell signal transduction (Sehnke et al., 2000; Wang et al., 2015).

Gene expression can also be regulated at translational and post-translational levels. We found that a number of DEPs involved in these processes were up-regulated in Pf9279-4, such as P0 60S, 50S ribosomal protein L12, elongation factor Tu, PDI and ETIF5A. These proteins are involved in protein synthesis. Higher levels of these proteins in the SBPH-resistant line indicate that plant cells of Pf9279-4 may be more active in protein synthesis than those of 02428.

Changes in the abundance and activity of some important functional proteins during SBPH infestation might lead to the establishment of a new cellular homeostasis and a better resistance to the insect attack. Four major groups of functional proteins that were identified from DEPs between the SBPH-resistant and susceptible lines were antioxidants, stress response, glycolysis and energy metabolism.

ROS produced during stress conditions might act as a signaling molecule for stress responses. It can also cause damage to cellular components. Plants can maintain the ROS level through sophisticated mechanisms such as scavenging them by antioxidant defense proteins (Mahmood et al., 2006). A number of DEPs in this category that were higher in Pf9279-4 than in 02428 at least in one time point were GSH-Px, glutathione S-transferase (GSTs) and isoflavone reductase-like protein, IRLs. GSH-Px can protect the membrane system in plants under stress. Under the action of GSTs, GSH combines with toxic substances to protect tissues. Isoflavone reductase is the key enzyme to synthesize isoflavone, which plays an important role in removing ROS (Kim et al., 2010) and styrene acrylic pigment derivatives.

DEPs that belong to stress response include ABA/WDS induced protein, glycin-rich RNA binding protein (GR-RBP), mannose-binding lectin (MBL), chitinase III-like protein, salt stress root protein (RS1), ricin B-related lectin domain containing protein (RBRL) and T-complex protein. The expression levels of these proteins were up-regulated in Pf9279-4 than 02428 at 6 h and 12 h after SBPH infestation. These proteins generally have positive effects on stress tolerance (Liang et al., 2013; He et al., 2017). These data indicate that Pf9279-4 may have a better stress tolerance capacity.

DEPs that are involved in glycolysis were PFK, GAPDH and PGK. The levels of these protein were up-regulated in Pf9279-4 at 6 or 12 h post SBPH infestation than 02428. In glycolysis, PGK generates two ATPs and GAPDH produces NADH+H^+^. The high levels of these proteins in Pf9279-4 might produce more energy for defense processes.

DEPs involved in energy metabolism that were up-regulated in Pf9279-4 under SBPH infestation conditions were Nod factor binding lectin- phosphohydrolase, LNP and Apyrase. These proteins participate in oxidative phosphorylation, photophosphorylation or ATP synthesis under the impetus of the transmembrane proton power (Pradet and Raymond, 1983) and they play an important role in metabolism and photosynthesis.nucleotide.

### Global view of the host response post SBPH infestation

Pests can induce the accumulation of SA in plant (Reymond et al., 2000). The free SA can be transported in phloem, which is the site of SBPH sucking the rice plants (Basyouni et al., 1964). The SA carboxyl methyltransferase catalyzes the formation of MeSA, which can reduce planthoppers infestation, using free SA and S-adenosyl-L-methionine. S-adenosylmethionine synthetase (SAM synthetase) catalyzes the formation of S-adenosyl-L-methionine. In this study, the protein level of SAM synthetase was up-regulated in Pf9279-4 than in 02428 at 12 h after SBPH infestation.

SA can combine with CAT protein and inhibit CAT activity, which leads to the reduction of H_2_O_2_ decomposition. H_2_O_2_ has been shown to have anti-insect activity (Zhu-Salzman et al., 2004). The activity of CAT in Pf9279-4 was lower than in 02428 at all the time points during SBPH infestation, which may lead to the higher contents of H_2_O_2_ in Pf9279-4 than in 02428. Down-regulation of CAT has been observed in sorghum after greenbug infestation (Zhu-Salzman et al., 2004). Higher SOD activity in combination with lower CAT activity observed in Pf9279-4 may potentially lead to a higher level of H_2_O_2_, as SOD catalyzes the conversion of ${\text{O}}_{2}^{-}$ into H_2_O_2_. The higher concentration of H_2_O_2_ can be one of the potential factors that contributes to Pf9279-4 resistance to SBPH.

Higher levels of GSH, GSH-px, and IDH in Pf9279-4 after SBPH infestation may represent a better capacity of maintaining redox homeostasis during SBPH infestation. GSH is derived from Glutamine and GSSG under the action of cysteine synthase and GSH-Px. IDH plays a role in maintaining the redox balance between the cytoplasm and the mitochondria, i.e., IDH transfers the oxidative GSH from mitochondria to cytoplasm (Yang et al., 2002; Maeng et al., 2004). As SBPH infestation causes oxidative stress in plants, the better capacity of maintaining redox homeostasis could potentially improve plant tolerance to SBPH.

Polyamines have the direct action in insect resistance (Alcázar et al., 2010). S-adenosy-L-methionine derived from methionine (Met) under the action of SAM synthetase was an important substance for polyamine production. The level of SAM synthetase was higher in Pf9279-4 at 12 h after SBPH infestation than 02428.

**Figure 9:**
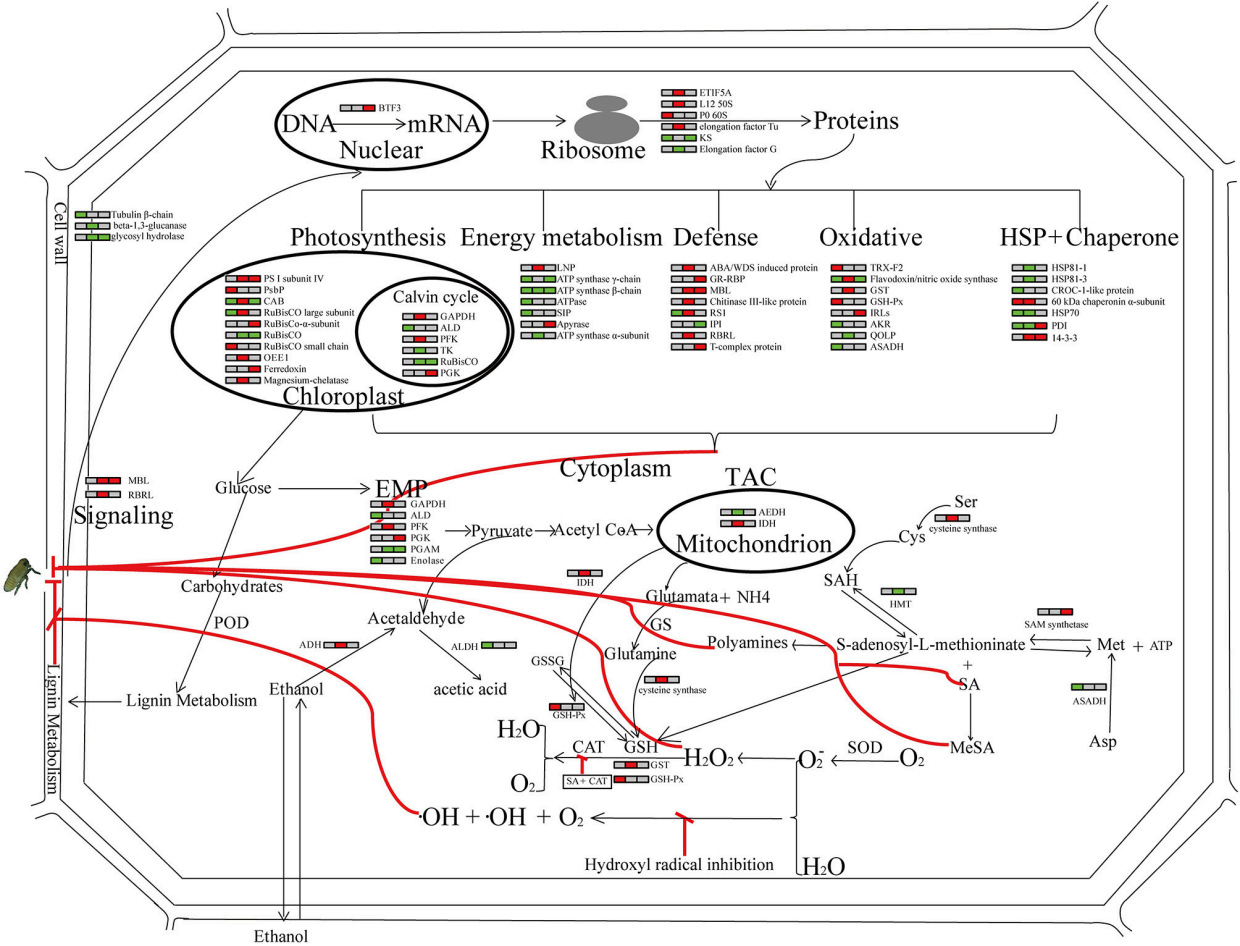
Global view of the host response post SBPH infestation. Relative abundance of proteins in the leaf sheath of the SBPH-resistant line Pf9279-4 at 0, 6, and 12 h post SBPH infestation in comparison with the SBPH-susceptible line 02428 are indicated by colors. Red represents that the expression levels of DEPs are up-regulated in Pf9279-4 rice plants at the indicated time point of SBPH infestation. Green represents that the expression levels of DEPs are down-regulated in Pf9279-4 rice plants. Gray represents no changes in protein abundance. Each horizontal row represents the DEPs. The left, middle and right of each horizontal row is the time point of 0, 6, and 12 h post SBPH infestation, respectively. All abbreviations for metabolites are defined in the text or table in which they first appear.

A concerted action of these proteins may contribute to Pf9279-4 resistance to SBPH. A simple model of SBPH infestation stress responses is outlined in Figure 9.

## Author contributions

YD, XC, and WZ designed the experiments; YD participated in data analysis and wrote the article; GX, YY, XC, XW, CLY, JZ, WF, and QM revised it critically for important intellectual content; CQY and JC approved the version to be published.

### Conflict of interest statement

The authors declare that the research was conducted in the absence of any commercial or financial relationships that could be construed as a potential conflict of interest.
