# Supplementary material for: Comparative Proteomic Analysis of Susceptible and Resistant Rice Plants during Early Infestation by Small Brown Planthopper
Source: Front Plant Sci. 2017 Oct 17;8:1744. doi: 10.3389/fpls.2017.01744 (PMC5651024; doi:10.3389/fpls.2017.01744)
Supplement: Supplementary file 1 [file Table1.PDF]

Supplementary Table S1. Primer sequences used in qPCR

| Protein Name | Forward primer | Reverse primer |
|---|---|---|
| Magnesium-chelatase | GCGTTCCGTGAGTCCTACTTGG | GTGTCCCTGC CTTTGAGTGC |
| 70 kDa heat shock protein, HSP70 | TCTACACCACCATCACCCGC | CCCGTTGAAGAAGTCCTGGAG |
| Heat shock protein 81-1, HSP81-1 | GGACTAAGGAGTTTATGGAGGCA | CGTGTGACAGTGAAGGACCC |
| Heat shock protein 81-3, HSP81-3 | TGACAAACGACTGGGAGGAACA | GGTCTTCAGAATCAACAATGCCC |
| Isopentenyl pyrophosphate:Dimethyllallyl pyrophosphate isomerase, IPI | GGGCTTTCAGTGTATTCCT | GTCAACTGGCACATCTTCA |
| ABA/WDS induced protein | CACCACCACCACCTGTTCCA | GTGCTGCTTCTCCTCCTTCTTGTA |
| Salt stress root protein RS1, RS1 | ACAAGGATGGAAAGAAGGC | CAGGACCGTTCTTCTTGATT |
| Eukaryotic translation initiation factor 5A, ETIF5A | GCCCTCCTCCCACAACT | GCAGACATCACGGTAACAATC |
| S-adenosylmethionine synthetase, SAM synthetase | GACCATCTTCCACCTCAACCC | GCTGGCGACGATGCTCTT |
| UDP-glucose pyrophosphorylase | GCGGAGCAGATCGAGTGGAG | CATTAGAAGCAAAGGGACATT |
| Glutathione S-transferase, GSTs | CCGCAAGAACAAACCAGA | CATCATCGGACGGATTAGGC |
| Glutathione peroxidase, GSH-Px | AGTGGCAAAGATGTGGACC | TTCAGAAACTTGTAGATGGGTG |
| Oligopeptidase A-like | CATTATGAAACAGGCGAACT | GGAAGAGGAGCAAGAACC |
| 14-3-3 | GGCTGAAAGGTATGAGGAGA | GCAGTAGATGAGGGCACAAG |
| Flavodoxin/nitric oxide synthase | CGGTGAAGGTCTATGTCGT | TTCCATCAGCCTCAGTAAGT |
| NADP-isocitrate dehydrogenase, IDH | AACCAGCACAAACAGCAT | TTGAAGACCCGTGAATAA |
| ATP synthase γ chain | CTACTCGCTGTTCGTGTCCG | CCTTCCTTGGTGGTGAGCC |
| Protein of unknown function DUF538, DUF538 | GTGGTACGCGGGCGAGGTGA | CGGAGGGGCTGAGGACGAT |
| 60S acidic ribosomal protein P0, P0 60S | CCTGCTCGTGTTGGTCTTG | CCCACCTTGTCACCCTTCT |
| CROC-1-like protein | ATCCATCGGCAGTCGTCG | GTCTGGGTAGTCCTTGTCGC |
| Asparaginyl-tRNA synthetase, KS | CCCAAGACCAAGCTCACGC | ATCTCACCAGCACCCTCAC |
| Chloroplast inorganic pyrophosphatase, SIP | GGGGTGTTCCACTTCGTC | TCTTGGTGTCCTGCTTGAT |
| Protein disulfide-isomerase, PDI | TTGTGGTCGCTGATAACG | GACGAAGTACAGGGTGGG |
| Transcription factor BTF3, BTF3 | CCTGATAACCTGGACAACC | CTCTTCCACCTCCGCTTC |
| Actin-122 | GAGTATGATGAGTCGGGTCCAG | ACACCAACAATCCCAAACAGAG |
