# Supplementary material for: Comparative Proteomic Analysis of Susceptible and Resistant Rice Plants during Early Infestation by Small Brown Planthopper
Source: Front Plant Sci. 2017 Oct 17;8:1744. doi: 10.3389/fpls.2017.01744 (PMC5651024; doi:10.3389/fpls.2017.01744)
Supplement: Supplementary file 2 [file Table2.PDF]

Supplementary Table S2. The scores of SBPH resistance in tested varieties

Variety	Number of seedings tested	Resistance score	Evaluation for resistance
Oryza officinalis	16	0	HR
Pf9279-4	16	2.8	R
02428	16	8.8	HS
