# Supplementary material for: Comparative Proteomic Analysis of Susceptible and Resistant Rice Plants during Early Infestation by Small Brown Planthopper
Source: Front Plant Sci. 2017 Oct 17;8:1744. doi: 10.3389/fpls.2017.01744 (PMC5651024; doi:10.3389/fpls.2017.01744)
Supplement: Supplementary file 3 [file Table3.PDF]

Supplementary Table S3 Differentially expressed protein spots identified by 4800 Plus MALDI TOF/TOF™ at three time points in rice cultivars Pf9279-4

NO.[a]	Accession NO.[b]	Protein Name	Accession NO.[c]	Time[d] (h)	Ratio[e]	*P*-value (t-test)	Mr/pI[f]	NP[g]	Expected value	SC%[h]	Protein Score	Total Ion C.I.%
**Response to stress**												
4[+]	tr|A2WY84|	Thioredoxin F-type 2(TRX-F2)	Os01g0913000	0	+10.20	0.031	19.73/8.81	7	0.00088	38%	83	99.797
16	sp|Q7XT99|	Probable aldo-keto reductase 2, AKR	Os04g0338000	0	-2.11	0.042	38.21/6.03	20	2.1e-040	45%	449	100
10	tr|Q6ESJ0|	glutathione peroxidase, GSH-Px	Os02g0664000	0	+3.16	0.048	25.83/9.42	10	1.7e-012	46%	170	100
23	tr|B8AK88|	Aspartate-semialdehyde dehydrogenase, ASADH	Os03g0760700	0	-2.50	0.031	40.15/6.73	13	4.2e-030	42%	346	100
33[+]	sp|A2WMG6|	salt stress root protein RS1, RS1	Os01g0233000	0	-5.48	0.039	21.79/4.92	9	1.7e-035	64%	400	100
34[+]	sp|A2WMG6|	salt stress root protein RS1, RS1	Os01g0233000	0	-11.2	0.031	21.79/4.92	5	2.7e-020	33%	248	100
38	sp|Q0JPA6|	salt stress root protein RS1, RS1	Os01g0233000	6	+29.72	0.005	21.79/4.92	10	2.1e-036	46%	409	100
41	sp|A2WMG6|	Salt stress root protein RS1, RS1	Os01g0233000	6	+9.14	0.023	21.79/4.92	7	2.7e-019	42%	238	100
32	tr|A2ZMH6|	CROC-1-like protein	Os12g0605400	0	-5.12	0.046	16.65/6.42	16	3.4e-019	93%	237	100
20	tr|Q6Z7L1|	Heat shock 70 kDa protein, HSP70	Os02g0774300	0	-2.38	0.037	72.85/5.49	18	1.7e-048	34%	530	100
78[+]	tr|Q943K7|	70 kDa heat shock protein, HSP70	Os03g0276500	6	-2.12	0.026	70.91/5.10	6	0.033	16%	67	99.232
98	tr|Q0J0U8|	Heat shock protein 81-3, HSP81-3	Os09g0482400	6	-2.00	0.027	80.13/4.98	28	1.7e-059	44%	640	100
80	sp|Q0J4P2|	Heat shock protein 81-1, HSP81-1	Os08g0500700	6	-2.13	0.015	80.14/5.00	27	1.7e-060	41%	650	100
99	sp|Q0J4P2|	Heat shock protein 81-1, HSP81-1	Os08g0500700	6	-2.27	0.028	80.14/5.00	32	2.7e-086	51%	908	100
67	tr|Q9FTY4|	Ricin B-related lectin domain containing protein, RBRL	Os01g0104400	6	+2.03	0.028	30.13/6.27	12	6.7e-050	44%	544	100
87	tr|Q7EYM8|	Quinone oxidoreductase-like protein, QOLP	Os08g0379400	6	-2.38	0.026	39.56/7.63	15	8.4e-050	53%	543	100
18	tr|Q9LLR2|	Aldehyde dehydrogenase, ALDH	Os09g0440300	0	-2.20	0.033	59.28/6.33	17	2.7e-023	32%	278	100
57[+]	tr|B8B9C5|	Alcohol dehydrogenase, ADH	Os08g0545200	6	+2.64	0.024	39.20/6.15	3	4.2e-009	14%	136	100
11	tr|A2ZJH7|	60 kDa chaperonin α-subunit	Os12g0277500	0	+2.30	0.032	61.09/5.12	17	6.7e-044	37%	484	100
69	tr|A2ZJH7|	60 kDa chaperonin α-subunit	Os12g0277500	6	+2.01	0.038	61.09/5.12	28	5.3e-072	63%	765	100
60[+]	tr|Q53JF7|	ABA/WDS induced protein	Os11g0167800	6	+2.34	0.028	15.46/6.20	4	3.4e-015	50%	197	100

NO.[a]	Accession NO.[b]	Protein Name	Accession NO.[c]	Time[d] (h)	Ratio[e]	*P*-value (t-test)	Mr/pI[f]	NP[g]	Expected value	SC%[h]	Protein Score	Total Ion C.I.%
63	tr|Q53JF7|	ABA/WDS induced protein	Os11g0167800	6	+2.27	0.033	15.46/6.20	10	2.7e-063	82%	678	100
95	tr|Q69RN2|	Chitinase III-like protein	Os07g0500300	6	-4.23	0.018	18.91/6.51	6	3.4e-044	61%	487	100
103	tr|Q69RN2|	Chitinase III-like protein	Os07g0500300	6	-3.09	0.029	18.91/6.51	8	1.1e-064	65%	692	100
104	tr|Q69RN2|	Chitinase III-like protein	Os07g0500300	6	-4.04	0.005	18.91/6.51	4	2.1e-017	51%	219	100
54[+]	tr|Q0JJ25|	glutathione S-transferase, GSTs	Os01g0764000	6	+2.67	0.017	23.96/5.77	8	4.2e-012	44%	166	100
21	tr|Q8LQN2|	Flavodoxin/nitric oxide synthase	Os01g0784800	0	-2.41	0.033	21.69/6.06	6	8.4e-019	43%	233	100
44	tr|Q8LQN2|	Flavodoxin/nitric oxide synthase	Os01g0784800	6	+6.25	0.013	21.69/6.06	6	2.7e-040	52%	448	100
74	tr|Q8LQN2|	Flavodoxin/nitric oxide synthase	Os01g0784800	6	+2.12	0.035	21.69/6.06	5	4.2e-050	48%	546	100
133	tr|Q8LQN2|	Flavodoxin/nitric oxide synthase	Os01g0784800	12	-2.56	0.013	21.69/6.06	5	1.1e-018	48%	232	100
75	tr|A0A0N7KSH4|	Mannose-binding lectin, MBL	Os11g0165700	6	+3.65	0.028	19.91/5.16	11	1.1e-076	63%	812	100
39	tr|A2ZBS9|	Mannose-binding lectin, MBL	Os11g0165700	6	+17.56	0.013	17.53/5.13	10	8.4e-089	66%	933	100
76	tr|A2ZBS9|	Mannose-binding lectin, MBL	Os11g0165700	6	+7.17	0.014	17.53/5.13	7	2.1e-039	64%	439	100
106	tr|A2ZBS9|	Mannose-binding lectin, MBL	Os11g0165700	12	+13.25	0.019	17.53/5.13	12	8.4e-084	72%	883	100
136[+]	tr|Q8GVZ0|	Isopentenyl pyrophosphate: dimethyllallyl pyrophosphate isomerase, IPI	Os07g0546000	12	-2.56	0.033	27.32/4.90	11	3.4e-011	63%	157	100
120	tr|A2WJQ7|	Isoflavone reductase-like protein, IRLs	Os01g0106400	12	+2.39	0.036	33.48/5.69	15	1.3e-048	63%	531	100
107	tr|B7SDF0|	Glycin-rich RNA binding protein, GR-RBP	Os12g0632000	12	+9.04	0.032	16.03/6.32	19	6.7e-059	96%	634	100
**Photosynthesis**												
9	tr|B0FFP0|	23 kDa polypeptide of photosystem II PsbP	Os07g0141400	0	+3.99	0.031	20.02/5.56	9	1.3e-021	46%	261	100
116	tr|B0FFP0|	Chloroplast 23 kDa polypeptide of photosystem II	Os07g0141400	12	+3.70	0.032	20.02/5.56	9	4.2e-016	15%	175	100
37	tr|B8A8L8|	Photosystem II oxygen-evolving complex protein 1, OEE1	Os01g0501800	6	+2.11	0.015	34.87/6.10	10	1.1e-030	44%	352	100
40	tr|B8A8L8|	Photosystem II oxygen-evolving complex protein 1, OEE1	Os01g0501800	6	+11.72	0.010	34.87/6.10	13	1.7e-044	47%	490	100

NO.[a]	Accession NO.[b]	Protein Name	Accession NO.[c]	Time[d] (h)	Ratio[e]	*P*-value (t-test)	Mr/pI[f]	NP[g]	Expected value	SC%[h]	Protein Score	Total Ion C.I.%
43	tr|Q6Z3V7|	Putative Photosystem I reaction center subunit IV, PS I subunit IV	Os05g0560000	6	+5.00	0.014	15.54/9.64	6	3.4e-042	61%	467	100
111	tr|Q6Z3V7|	Photosystem I reaction center subunit IV, PS I subunit IV	Os07g0435300	12	+4.90	0.020	15.54/9.64	7	1.7e-038	63%	430	100
51	tr|H2KW47|	chlorophyll A-B binding protein, CAB	Os01g0600900	6	+3.18	0.019	23.92/4.73	7	5.3e-008	38%	125	100
58	tr|Q5ZA98|	chlorophyll A-B binding protein, CAB	Os01g0600900	6	+2.55	0.046	26.17/5.75	4	3.4e-011	13%	157	100
64[+]	tr|A2X218|	chlorophyll A-B binding protein, CAB	Os02g0197600	6	+2.22	0.019	29.19/6.92	10	1.1e-008	47%	132	100
62	tr|A2YMN1|	chlorophyll A-B binding protein, CAB	Os07g0562700	6	+2.28	0.033	28.77/5.82	10	5.3e-037	40%	415	100
137[+]	tr|A2YMN1|	chlorophyll A-B binding protein, CAB	Os07g0562700	12	-2.80	0.036	28.77/5.82	9	3.4e-008	34%	127	100
144	tr|A2YMN1|	chlorophyll A-B binding protein, CAB	Os07g0562700	12	-2.02	0.036	28.77/5.82	10	2.7e-040	36%	448	100
27	tr|Q53N83|	chlorophyll A-B binding protein, CAB	Os01g0600900	0	-3.38	0.039	30.26/5.50	13	1.7e-037	58%	420	100
139	tr|Q53N83|	chlorophyll A-B binding protein, CAB	Os01g0600900	12	-4.96	0.022	30.26/5.50	12	2.7e-044	60%	488	100
66	sp|Q53RM0|	Magnesium-chelatase	Os03g0563300	6	+2.04	0.048	44.84/5.51	21	1.1e-055	54%	602	100
13	tr|G8CTM8|	Ribulose bisphosphate carboxylase activase small isoform, RuBisCO small chain	Os11g0707000	0	+2.08	0.037	47.90/5.85	27	5.3e-067	60%	715	100
47	tr|A0A140H518|	ribulose bisphosphate carboxylase large chain, RuBisCO large chain	Os10g0356000	6	+3.66	0.036	20.27/5.76	7	5.3e-020	54%	245	100
28	tr|A0A140H518|	ribulose bisphosphate carboxylase large chain, RuBisCO large chain	Os10g0356000	0	-3.86	0.031	20.27/5.76	10	1.7e-058	55%	630	100
73	tr|A0A0P0V969|	ribulose-1,5-bisphosphate carboxylase/ oxygenase large subunit, RuBisCO large chain	Os01g0791033	6	+2.34	0.031	29.71/6.11	13	5.3e-055	49%	595	100
70	tr|A0A0P0Y838|	ribulose-1,5-bisphosphate carboxylase/ oxygenase large subunit, RuBisCO large chain	Os12g0207600	6	+3.27	0.015	53.13/6.23	10	8.4e-012	24%	163	100

NO.[a]	Accession NO.[b]	Protein Name	Accession NO.[c]	Time[d] (h)	Ratio[e]	*P*-value (t-test)	Mr/pI[f]	NP[g]	Expected value	SC%[h]	Protein Score	Total Ion C.I.%
46	tr|E9KIP7|	ribulose bisphosphate carboxylase large chain, RuBisCO large chain	Os10g0356000	6	+4.54	0.014	53.67/6.33	25	8.4e-045	55%	493	100
72	tr|A0A088BVM4|	ribulose bisphosphate carboxylase large chain, RuBisCO large chain	Os10g0356000	6	+3.58	0.013	48.37/6.34	12	8.4e-031	36%	353	100
83	tr|A0A0P0Y677|	Ribulose bisphosphate carboxylase/ oxygenase activase, RuBisCO	Os11g0707000	6	-2.35	0.028	42.38/5.05	21	1.3e-085	65%	901	100
132	tr|H2KVX3|	Ribulose bisphosphate carboxylase/ oxygenase activase, RuBisCO	Os11g0707000	12	-2.18	0.039	39.62/6.66	19	3.4e-029	55%	337	100
122	tr|A2ZJH7|	RuBisCO subunit binding-protein alpha subunit, RuBisCo-α-subunit	Os12g0277500	12	+2.17	0.019	61.09/5.12	13	3.4e-044	30%	487	100
128	tr|A2ZJH7|	RuBisCO subunit binding-protein alpha subunit, RuBisCo-α-subunit	Os12g0277500	12	+2.26	0.021	61.09/5.12	16	6.7e-048	49%	524	100
91	tr|Q5VNW1|	transketolase, TK	Os06g0133800	6	-2.74	0.033	68.84/5.43	16	2.1e-008	27%	129	100
118	tr|Q6ZFJ3|	ferredoxin-NADP reductase, Ferredoxin	Os02g0103800	12	+2.89	0.036	40.64/7.98	10	1.1e-009	37%	142	100
143	tr|Q69Y18|	Alpha/beta hydrolase fold-3 domain containing protein, NYC3	Os06g0214800	12	-2.04	0.020	34.87/5.38	8	8.4e-012	39%	163	100
Protein metabolic process												
12	tr|Q9ZR35|	Reversibly glycosylated polypeptide	Os03g0599800	0	+2.19	0.031	41.35/5.82	13	6.7e-020	36%	244	100
7	tr|Q2QY46|	60S acidic ribosomal protein P0, P0 60S	Os12g0133050	0	+5.25	0.032	34.45/5.38	10	1.7e-032	35%	370	100
50	sp|O22386|	50S ribosomal protein L12	Os01g0662200	6	+3.27	0.033	18.58/5.36	4	6.7e-031	26%	354	100
42	tr|Q9AUW3|	eukaryotic translation initiation factor 5A, ETIF5A	Os03g0758800	6	+6.59	0.014	17.52/5.71	8	3.4e-013	55%	177	100
59[+]	tr|B8ADM7|	acyltransferase	Os01g0361500	6	+2.44	0.017	46.14/5.64	12	1.3e-016	51%	211	100
68	tr|B8AEQ9|	elongation factor Tu	Os02g0595700	6	+2.02	0.030	55.47/5.68	20	1.7e-091	46%	960	100

NO.[a]	Accession NO.[b]	Protein Name	Accession NO.[c]	Time[d] (h)	Ratio[e]	*P*-value (t-test)	Mr/pI[f]	NP[g]	Expected value	SC%[h]	Protein Score	Total Ion C.I.%
88+	tr|A2XVY3|	elongation factor G	Os04g0538100	6	-2.38	0.017	84.86/5.43	11	2.1e-009	24%	139	100
77+	tr|H2KWU8|	5-methyltetrahydropteroyltriglutamate-homocystein e methyltransferase	Os12g0624000	6	-2.04	0.045	84.58/5.93	5	1.3e-009	9%	141	100
82+	tr|H2KWU8|	5-methyltetrahydropteroyltriglutamate-homocystein e methyltransferase	Os12g0624000	6	-2.25	0.027	84.58/5.93	4	5.3e-006	10%	105	100
94	tr|Q6K9T1|	Oligopeptidase A-like	Os02g0830100	6	-3.53	0.038	86.16/5.76	20	2.7e-016	32%	208	100
35	tr|K4FHN8|	Protein disulfide-isomerase, PDI	Os11g0199200	0	-14.96	0.039	56.89/4.97	30	4.2e-055	58%	596	100
101	tr|K4FHN8|	Protein disulfide isomerase-like 1-1, PDI	Os11g0199200	6	-4.90	0.035	56.89/4.97	20	5.3e-039	45%	435	100
97	sp|Q53LQ0|	Protein disulfide isomerase-like 1-1,PDI	Os11g0199200	6	-6.77	0.020	56.82/5.01	24	1.1e-041	49%	462	100
113	sp|Q53LQ0|	Protein disulfide isomerase-like 1-1, PDI	Os11g0199200	12	+4.14	0.009	56.82/5.01	33	1.3e-050	57%	551	100
49	tr|Q0JCM0|	G-box binding factor, 14-3-3	Os04g0462500	6	+3.35	0.037	29.85/4.76	13	1.3e-032	53%	371	100
108	tr|Q0J5J5|	G-box binding factor, 14-3-3 protein	Os08g0430500	12	+6.73	0.019	28.81/4.78	4	3.4e-011	21%	157	100
127	sp|Q6ZKC0|	G-box binding factor, 14-3-3 protein	Os08g0430500	12	+3.00	0.022	28.81/4.78	19	1.3e-040	63%	451	100
105	sp|Q2R2W2|	14-3-3-like protein GF14-D	Os04g0462500	12	+22.97	0.013	29.24/4.83	6	6.7e-017	37%	214	100
114	tr|A2XL95|	14-3-3-like protein	Os03g0710800	12	+3.72	0.009	29.16/4.81	16	1.3e-062	76%	671	100
123+	tr|A2YYZ3|	acetyl-CoA acetyltransferase	Os09g0252100	12	+2.14	0.021	41.00/6.15	15	2.1e-019	66%	239	100
31	tr|Q93WM3|	Asparaginyl-tRNA synthetase, KS	Os01g0372700	0	-4.87	0.031	62.55/5.68	20	2.7e-032	37%	368	100
134+	tr|Q93WM3|	Asparaginyl-tRNA synthetase, KS	Os01g0372700	12	-2.77	0.013	62.55/5.68	16	8.4e-008	30%	123	100
Carbohydrate metabolic process												
26	tr|A1YQJ3|	enolase	Os10g0167300	0	-3.08	0.031	47.94/5.41	17	3.4e-027	54%	317	100
14	tr|A2ZBX1|	Fructose-bisphosphate aldolase, ALD	Os11g0171300	0	-2.03	0.031	41.98/6.38	20	1.3e-086	48%	911	100
17	sp|Q8H8T0|	UDP-arabinopyranose mutase 1	Os03t0599800	0	-2.15	0.031	41.32/5.82	27	8.4e-051	60%	553	100
45+	tr|Q7F280|	NADP-isocitrate dehydrogenase, IDH	Os01g0654500	6	+6.18	0.020	46.01/6.34	12	4.2e-006	37%	106	99.994

NO.[a]	Accession NO.[b]	Protein Name	Accession NO.[c]	Time[d] (h)	Ratio[e]	*P*-value (t-test)	Mr/pI[f]	NP[g]	Expected value	SC%[h]	Protein Score	Total Ion C.I.%
48[+]	tr|A0A0P0XB45|	Fructokinase, pfkB family, PFK	Os08g0113100	6	+3.65	0.028	29.74/5.34	8	1.1e-016	40%	212	100
56[+]	tr|A0A0P0XBB4|	glyceraldehyde-3-phosphate dehydrogenase, GAPDH	Os08g0126300	6	+2.65	0.014	15.46/6.20	1	1.7e-006	27%	110	100
65	tr|Q01IJ6|	glyceraldehyde-3-phosphate dehydrogenase, GAPDH	Os04g0459500	6	+2.11	0.028	42.69/7.62	7	5.3e-025	20%	295	100
79	tr|Q5QMK7|	2,3-bisphosphoglycerate-independent phosphoglycerate mutase, PGAM	Os01g0817700	6	-2.13	0.034	60.75/5.42	33	1.1e-080	71%	852	100
135	tr|Q5QMK7|	2,3-bisphosphoglycerate-independent phosphoglycerate mutase, PGAM	Os01g0817700	12	-2.74	0.013	60.75/5.42	14	4.2e-017	31%	216	100
92[+]	sp|Q7XDC8|	Malate dehydrogenase, AEDH	Os10g0478200	6	-2.83	0.023	35.55/5.75	3	2.1e-006	19%	109	100
112	sp|Q2R1V8|	GDP-mannose 3,5-epimerase 2	Os01g0367100	12	+4.57	0.030	42.10/5.75	15	5.3e-033	48%	375	100
117	tr|A2YMB7|	beta-amylase	Os07g0543300	12	+3.27	0.024	55.12/5.30	27	1.1e-084	56%	892	100
129	tr|A2YMB7|	beta-amylase	Os07g0543300	12	+2.60	0.045	55.12/5.30	25	2.1e-045	58%	499	100
130	tr|A2YMB7|	beta-amylase	Os07g0543300	12	+3.32	0.010	55.12/5.30	13	3.4e-007	37%	117	100
125	tr|A2XHR1|	sucrose synthase	Os03g0401300	12	+2.12	0.036	92.87/5.94	9	2.7e-016	12%	208	100
126[+]	tr|B8AIH2|	phosphoglycerate kinase protein, PGK	Os02g0169300	12	+2.11	0.024	42.08/5.64	5	8.4e-006	20%	103	100
145	tr|A4KC21|	UDP-glucose pyrophosphorylase	Os01g0264100	12	-3.98	0.024	51.64/5.59	24	8.4e-049	63%	533	100
Energy metabolism												
24	tr|A2XA10|	Chloroplast inorganic pyrophosphatase, SIP	Os02g0768600	0	-2.57	0.040	31.76/5.80	17	8.4e-052	40%	563	100
142	tr|Q0DX85|	Soluble inorganic pyrophosphatase, SIP	Os02g0768600	12	-7.97	0.031	32.98/5.59	15	2.7e-018	41%	228	100
22	tr|B8B107|	Vacuolar-type H+-ATPase subunit A1, ATPase	Os06g0662000	0	-2.46	0.040	68.43/5.20	30	1.7e-096	59%	1,010	100
52	tr|A2XZY2|	Nod factor binding lectin-nucleotide phosphohydrolase, LNP	Os11g0439600	6	+2.95	0.043	50.19/5.96	3	6.7e-015	13%	194	100
84	sp|P0C521|	ATP synthase α-subunit	Os01g0222500	6	-2.35	0.043	55.34/5.85	20	6.7e-050	48%	544	100

NO.[a]	Accession NO.[b]	Protein Name	Accession NO.[c]	Time[d] (h)	Ratio[e]	*P*-value (t-test)	Mr/pI[f]	NP[g]	Expected value	SC%[h]	Protein Score	Total Ion C.I.%
25	sp|P0C2Z8|	ATP synthase β chain	Os10g0355800	0	-2.70	0.031	53.92/5.38	26	1.7e-106	62%	1,110	100
93	sp|P0C2Z8|	ATP synthase β chain	Os10g0355800	6	-3.09	0.027	53.92/5.38	19	1.3e-079	54%	841	100
100	sp|P0C2Z8|	ATP synthase β chain	Os10g0355800	6	-2.95	0.035	53.92/5.38	30	1.7e-154	81%	1590	100
131	sp|P0C2Z8|	ATP synthase β chain	Os10g0355800	12	-2.06	0.019	53.92/5.38	20	2.1e-030	52%	349	100
29	tr|Q84NW1|	ATP synthase γ chain	Os07g0513000	0	-4.10	0.031	39.68/8.60	21	1.7e-067	46%	720	100
141[+]	tr|Q84NW1|	ATP synthase γ chain	Os07g0513000	12	-6.18	0.013	39.68/8.60	9	4.2e-011	28%	156	100
109	tr|Q53KM3|	Apyrase	Os03t0328400	12	+5.27	0.047	50.29/5.83	16	4.2e-044	38%	486	100
**Cell wall relatived protein**												
15	tr|A2YG29|	Tubulin β-chain	Os06g0671900	0	-2.07	0.034	50.09/4.73	19	2.7e-022	43%	268	100
86	tr|Q75K72|	putative beta-1,3-glucanase	Os05g0375400	6	-2.37	0.015	34.68/5.92	8	4.2e-052	41%	566	100
89	tr|A2Y2C8|	glycosyl hydrolase	Os05g0247100	6	-2.60	0.023	32.53/6.08	17	2.7e-064	48%	688	100
138	tr|A2Y2C4|	glycosyl hydrolase	Os05g0247100	12	-2.82	0.009	32.51/5.88	16	6.7e-060	41%	644	100
**Amino acide metabolism**												
81	tr|Q5JNB0|	cysteine synthase	Os01g0978100	6	-2.24	0.029	41.76/6.28	13	2.7e-010	38%	148	100
102	tr|Q5JNB0|	cysteine synthase	Os01g0978100	6	-2.21	0.036	41.76/6.28	23	1.7e-119	66%	1240	100
119	tr|A0A0R7VIP9|	S-adenosylmethionine synthetase, SAM synthetase	Os01g0323600	12	+2.52	0.034	42.87/5.68	19	8.4e-064	62	683	100
121	sp|Q6YZE2|	Glutamate-1-semialdehyde 2,1-aminomutase	Os08g0532200	12	+2.29	0.019	50.21/6.48	12	1.7e-025	35%	300	100
**Catalytic function**												
19	tr|Q940D3|	RNase S-like protein	Os09g0537700	0	-2.24	0.046	28.38/5.25	8	2.1e-032	37%	369	100
85[+]	tr|A0A0P0UZH3|	phospholipase D	Os01g0172400	6	-2.36	0.042	85.15/5.83	23	8.4e-020	29%	243	100
**Transcription**												
110[+]	tr|A0A0P0VS15|	transcription factor BTF3, BTF3	Os03g0109600	12	+5.15	0.019	16.34/4.48	5	0.00041	33%	86	99.985
**Others**												
3	tr|Q5Z6T5|	Protein of unknown function DUF538, DUF538	Os06g0538900	0	+10.97	0.031	17.61/5.36	6	1.3e-012	48%	171	100

NO.[a]	Accession NO.[b]	Protein Name	Accession NO.[c]	Time[d] (h)	Ratio[e]	*P*-value (t-test)	Mr/pI[f]	NP[g]	Expected value	SC%[h]	Protein Score	Total Ion C.I.%
55	tr|A4KC21|	UDP-glucose pyrophosphorylase	Os09g0553200	6	+2.67	0.014	51.64/5.59	27	3.4e-074	73%	787	100
96	tr|Q0DC43|	Formate dehydrogenase, FDH	Os06g0486800	6	-4.12	0.013	41.32/6.68	18	5.3e-041	63%	455	100
124[+]	tr|Q9LHY3|	Esterase, SGNH hydrolase-type	Os01g0214200	12	+2.13	0.036	26.46/5.12	9	0.00049	42%	85	98.663

[a]NO. corresponds to protein spot on gels shown in Figures 3, 4. In first column, the protein with "+" was the single peptide at the secondary level—ion match peptides and its spectra has been added in Supplementary Figure S8

[b]Accession NO. predicted protein in uniprot_Oryza_sativa database.

[c]Accession NO. was the ID in RAP-DB database

[d]Time was the time point after SBPH infestation

[e]Ratio was the expressed ratio of protein spot, "+ratio" show the expression level of the protein spot was up-regulated, "-ratio" show the expression level of the protein spot was down-regulated

[f]Mr/pI: Mr of molecular mass of predicted protein/pI of predicted protein.g[f]NP: Number of total matched peptides at PMF level and the secondary level—ion match peptidesh

[g]SC%: Sequence Coverage
