# Supplementary material for: Comparative Proteomic Analysis of Susceptible and Resistant Rice Plants during Early Infestation by Small Brown Planthopper
Source: Front Plant Sci. 2017 Oct 17;8:1744. doi: 10.3389/fpls.2017.01744 (PMC5651024; doi:10.3389/fpls.2017.01744)
Supplement: Supplementary file 8 [file Image1.PDF]

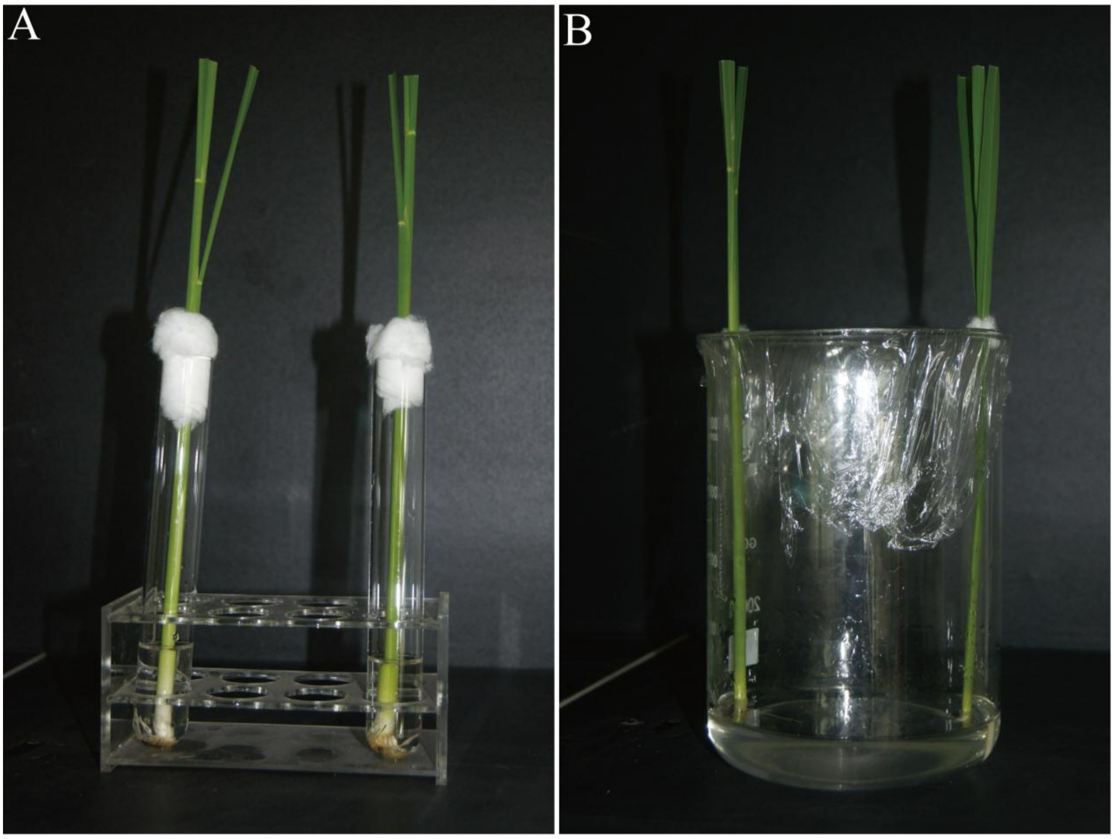

Supplementary Figure S1.| Experimental method of resistance analysis of Pf9279-4 and 02428 to SBPH. A was for the experiments of Life Span of SBPH Adult (days), Nymphal Duration (days), Number of Eggs, Survival Rate/% and Hatchability/%. B was for Value of Antixenosis/%. The left hand side was 02428 and the right hang side was Pf9279-4 in A and B, respectively.
