# Supplementary material for: Comparative Proteomic Analysis of Susceptible and Resistant Rice Plants during Early Infestation by Small Brown Planthopper
Source: Front Plant Sci. 2017 Oct 17;8:1744. doi: 10.3389/fpls.2017.01744 (PMC5651024; doi:10.3389/fpls.2017.01744)
Supplement: Supplementary file 9 [file Image2.PDF]

A

Repeat	Cy3-labeled	Cy5-labeled	Cy2-labeled
Gel1	50 μg (C1)	50 μg (T1)	50 μg internal standard
Gel2	50 μg (T2)	50 μg (C2)	50 μg internal standard
Gel3	50 μg (C3)	50 μg (T3)	50 μg internal standard

B

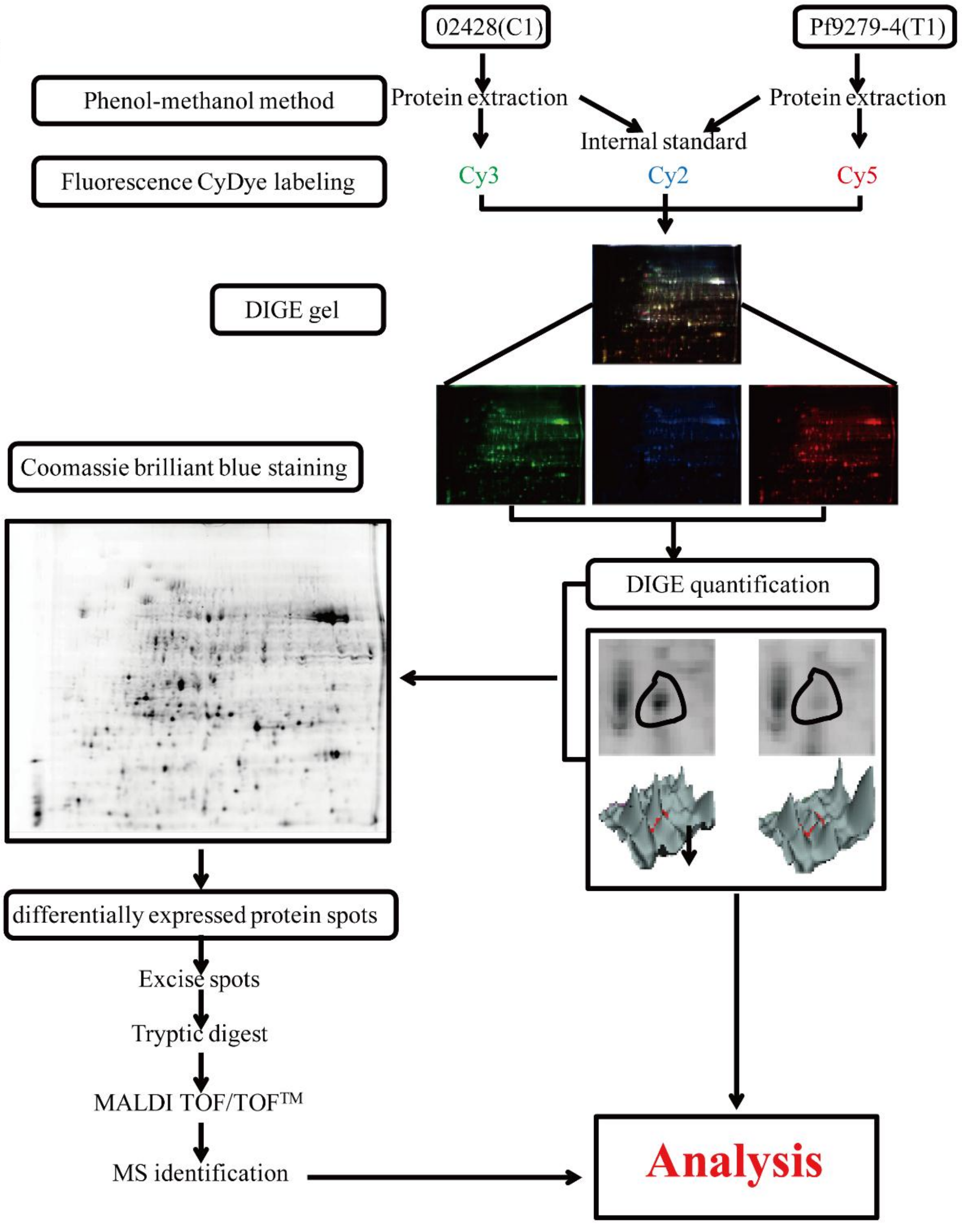

Supplementary Figure S2. | Experimental setup for fluorescent dye labeled and DIGE quantification. A, at each time point, three replicates derived from 02428 and Pf9279-4, resepectively. Cy3- and Cy5-labeled protein samples as well as the Cy2-internal standard were mixed together in one tube. B, schematic of DIGE labeling for a single gel1
