# Supplementary material for: Comparative Proteomic Analysis of Susceptible and Resistant Rice Plants during Early Infestation by Small Brown Planthopper
Source: Front Plant Sci. 2017 Oct 17;8:1744. doi: 10.3389/fpls.2017.01744 (PMC5651024; doi:10.3389/fpls.2017.01744)
Supplement: Supplementary file 10 [file Image3.PDF]

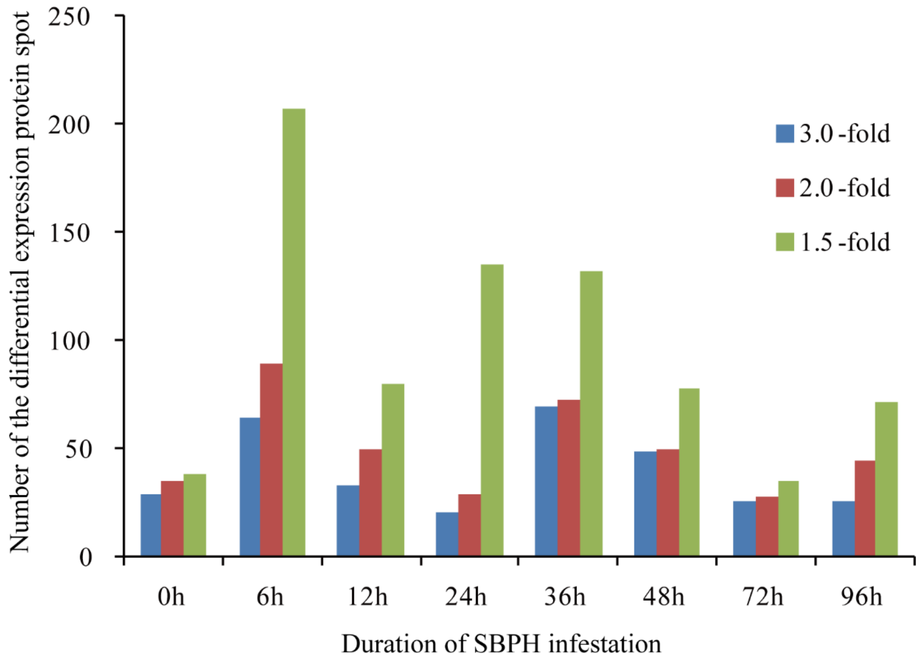

Supplementary Figure S3. | The number of differential expression protein spots. A cut-off value of 1.5-fold, 2.0-fold, 3.0-fold change was independently adopted to discriminate expression of proteins that were differentially altered. Rice plants had been infected with SBPH at 0 h, 6 h, 12 h, 24 h, 36 h, 48 h, 72 h and 96 h, respectively.
