# Supplementary material for: Comparative Proteomic Analysis of Susceptible and Resistant Rice Plants during Early Infestation by Small Brown Planthopper
Source: Front Plant Sci. 2017 Oct 17;8:1744. doi: 10.3389/fpls.2017.01744 (PMC5651024; doi:10.3389/fpls.2017.01744)
Supplement: Supplementary file 12 [file Image5.PDF]

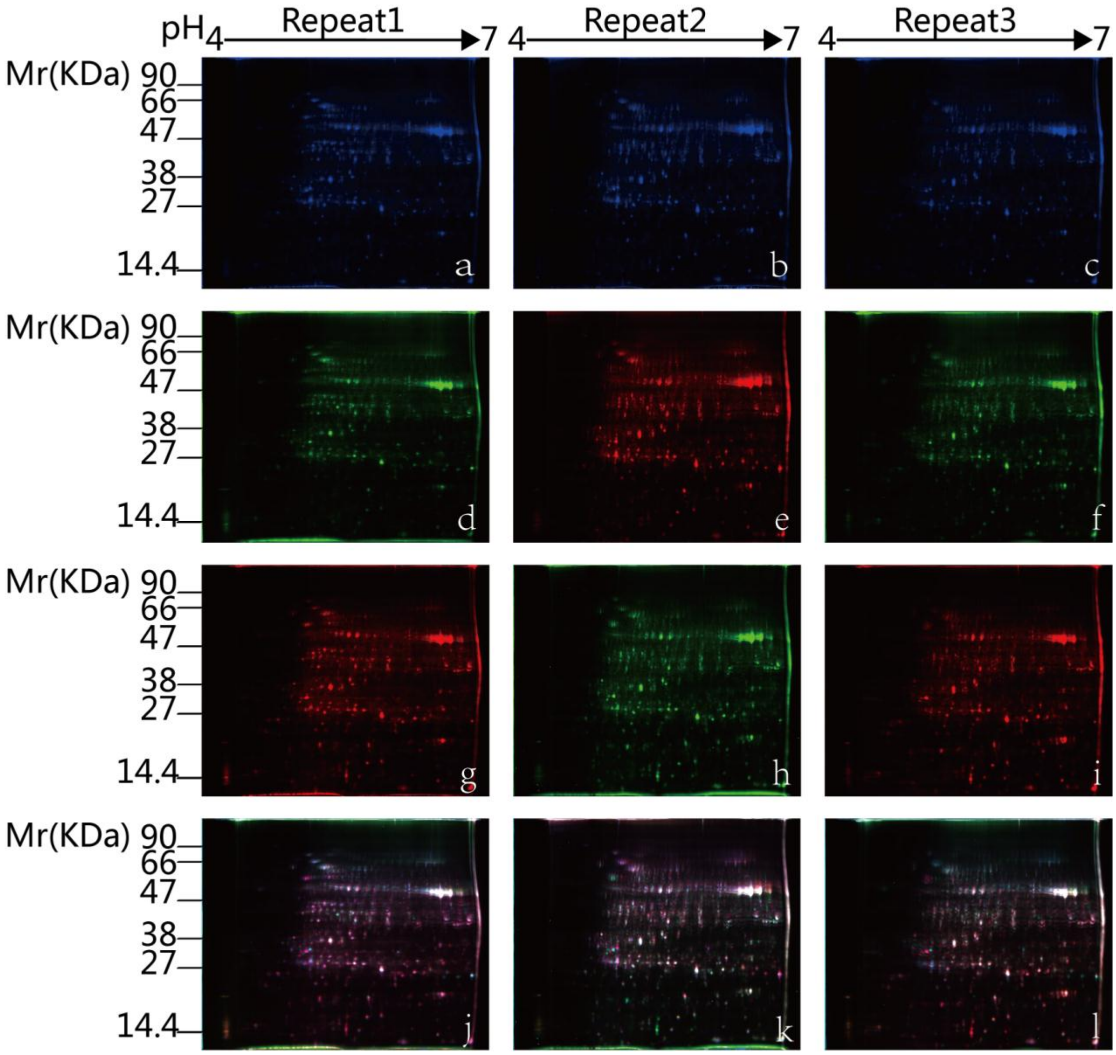

Supplementary Figure S5. | Three biological replicates and three triplicate gels at 6 h. Blue represents the proteins labeled by Cy2. Green represents the proteins labeled by Cy3 Red represents the proteins labeled by Cy5**.** a, b, c were the gels of the same amount of proteins from 02428 and Pf9279-4**.** d, e, f were the gels of proteins from 02428**.** g, h, I was the gels of proteins from Pf9279-4**.** j, k, l was superposition of triplicate gels, respectively.
