# Supplementary material for: Comparative Proteomic Analysis of Susceptible and Resistant Rice Plants during Early Infestation by Small Brown Planthopper
Source: Front Plant Sci. 2017 Oct 17;8:1744. doi: 10.3389/fpls.2017.01744 (PMC5651024; doi:10.3389/fpls.2017.01744)
Supplement: Supplementary file 13 [file Image6.PDF]

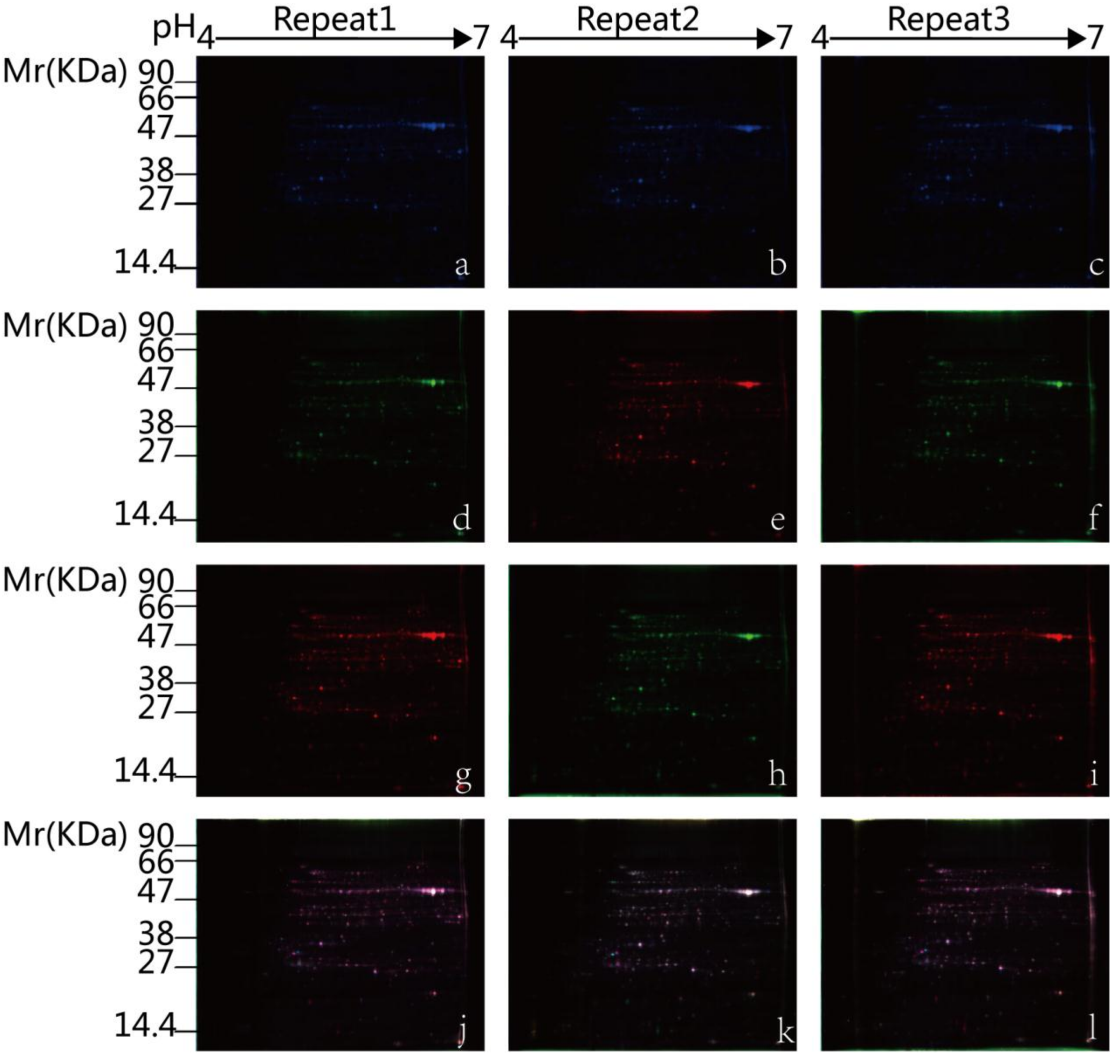

Supplementary Figure S6. | Three biological replicates and three triplicate gels at 12 h. Blue represents the proteins labeled by Cy2. Green represents the proteins labeled by Cy3 Red represents the proteins labeled by Cy5**.** a, b, c were the gels of the same amount of proteins from 02428 and Pf9279-4**.** d, e, f were the gels of proteins from 02428**.** g, h, I was the gels of proteins from Pf9279-4**.** j, k, l was superposition of triplicate gels, respectively.
