# Supplementary material for: Comparative Proteomic Analysis of Susceptible and Resistant Rice Plants during Early Infestation by Small Brown Planthopper
Source: Front Plant Sci. 2017 Oct 17;8:1744. doi: 10.3389/fpls.2017.01744 (PMC5651024; doi:10.3389/fpls.2017.01744)
Supplement: Supplementary file 14 [file Image7.PDF]

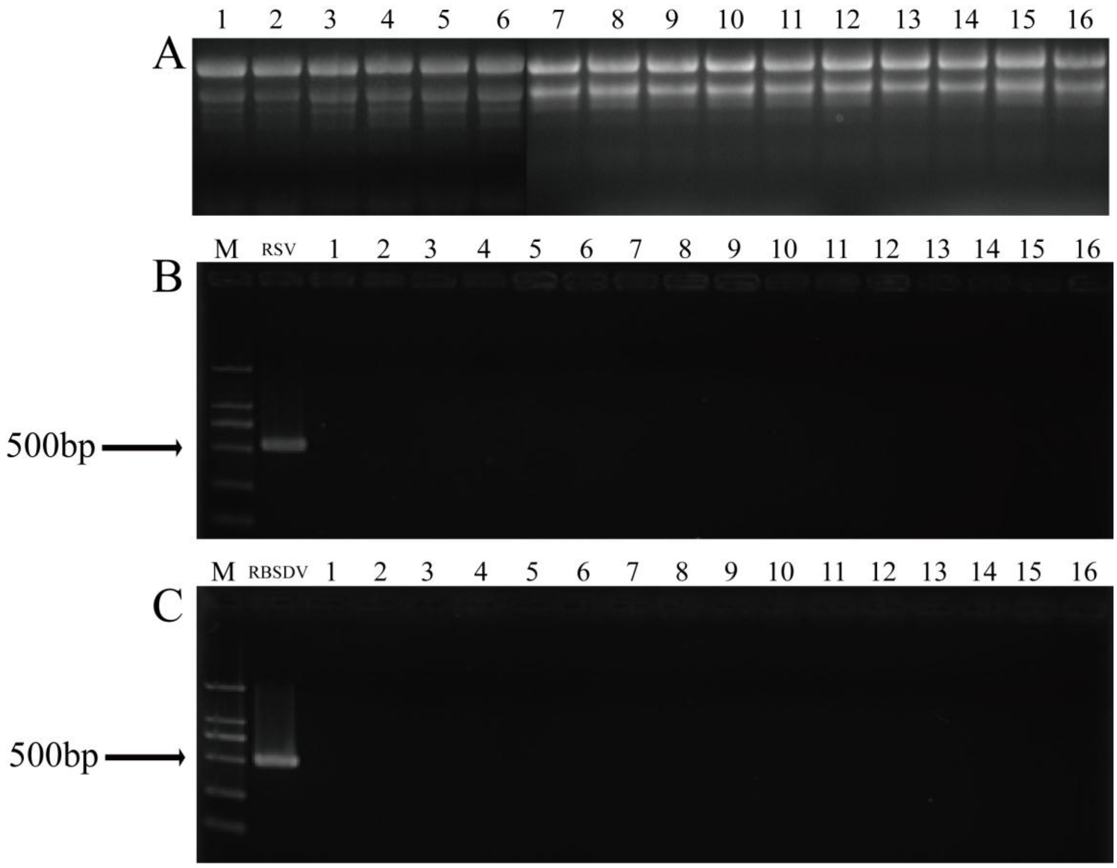

Supplementary Figure S7. | Reverse transcription polymerase chain reaction (RT-PCR) assay for detection of RSV and RBSDV in Pf9279-4 and 02428 after SBPH infestation. A, the RNA samples detection. B, the primers of RSV-CP to amply RSV. The column of RSV is positive control. C, the primers of RBSDV-P10 to amply RBSDV. The column of RBSDV is positive control. 1-16 were the 02428-0h, Pf9279-4-0h, 02428-6h, Pf9279-4-6h, 02428-12h, Pf9279-4-12h, 02428-24h, Pf9279-4-14h, 02428-36h, Pf9279-4-36h, 02428-48h, Pf9279-4-48h, 02428-72h, Pf9279-4-72h, 02428-96h and Pf9279-4-96h, respectively. The samples were fount to have no virues.
