# Supplementary material for: Comparative Proteomic Analysis of Susceptible and Resistant Rice Plants during Early Infestation by Small Brown Planthopper
Source: Front Plant Sci. 2017 Oct 17;8:1744. doi: 10.3389/fpls.2017.01744 (PMC5651024; doi:10.3389/fpls.2017.01744)
Supplement: Supplementary file 15 [file Image8.PDF]

Spot 4

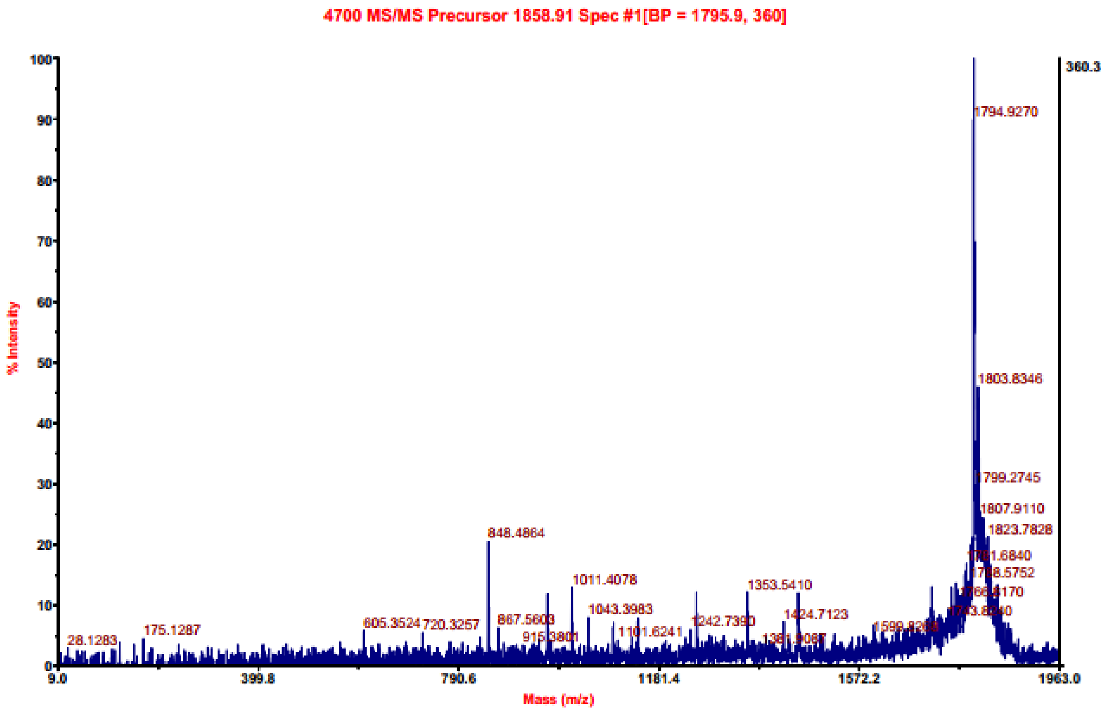

Spot 33

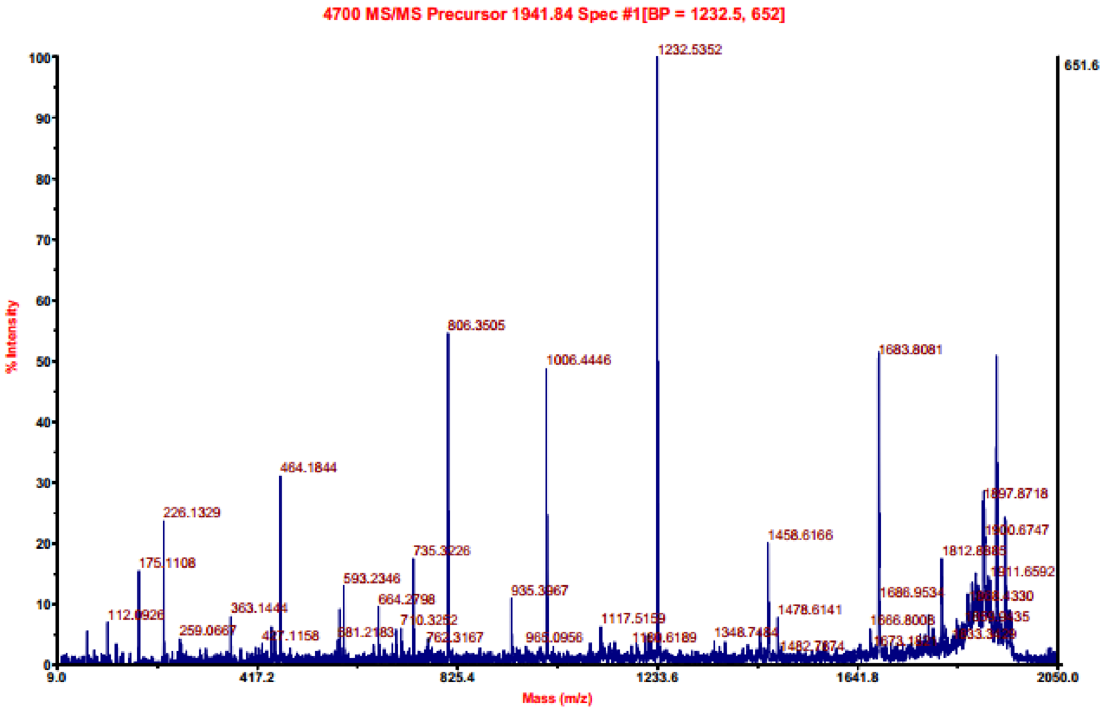

Spot 34

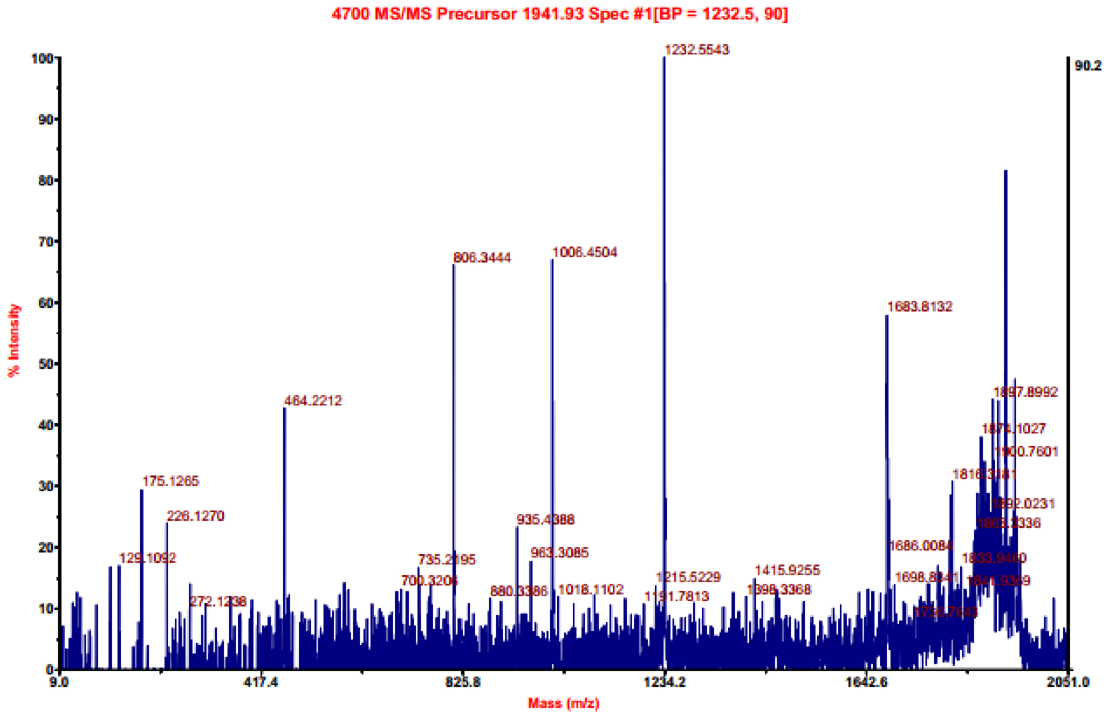

Spot 45

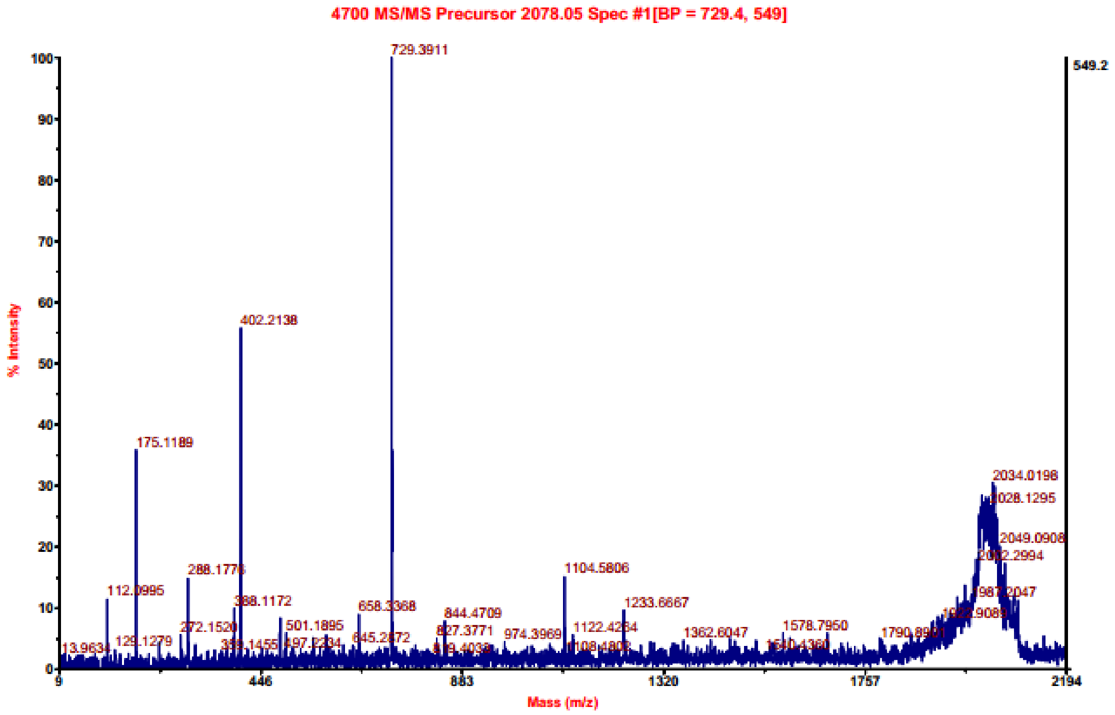

Spot 48

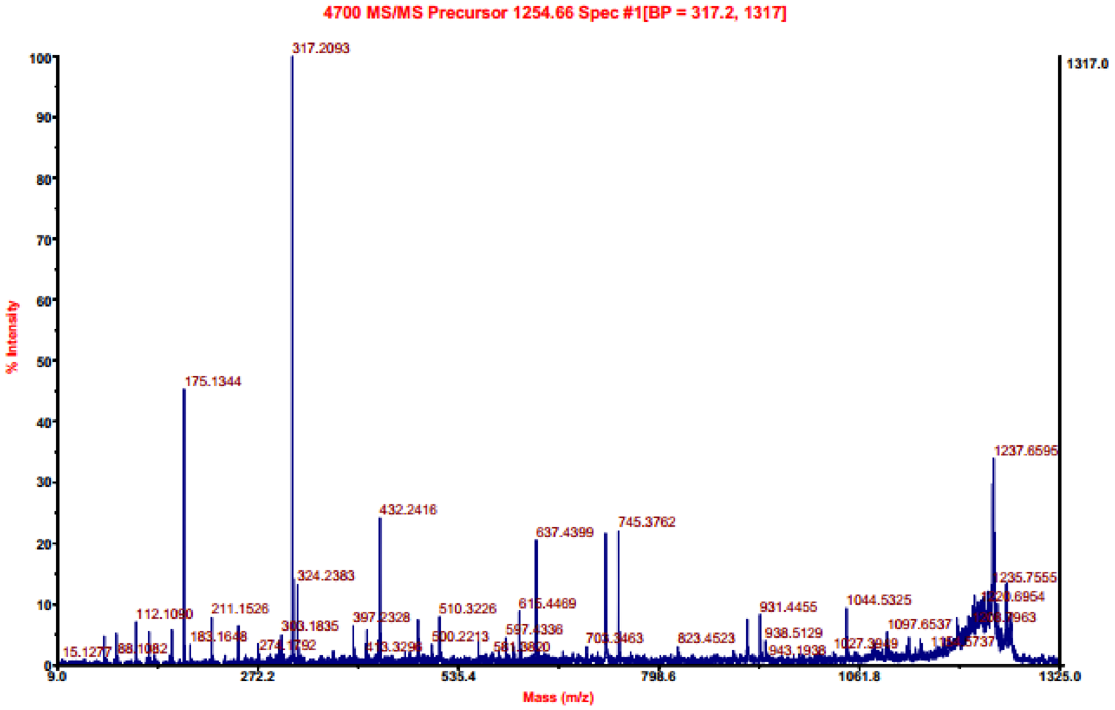

Spot 54

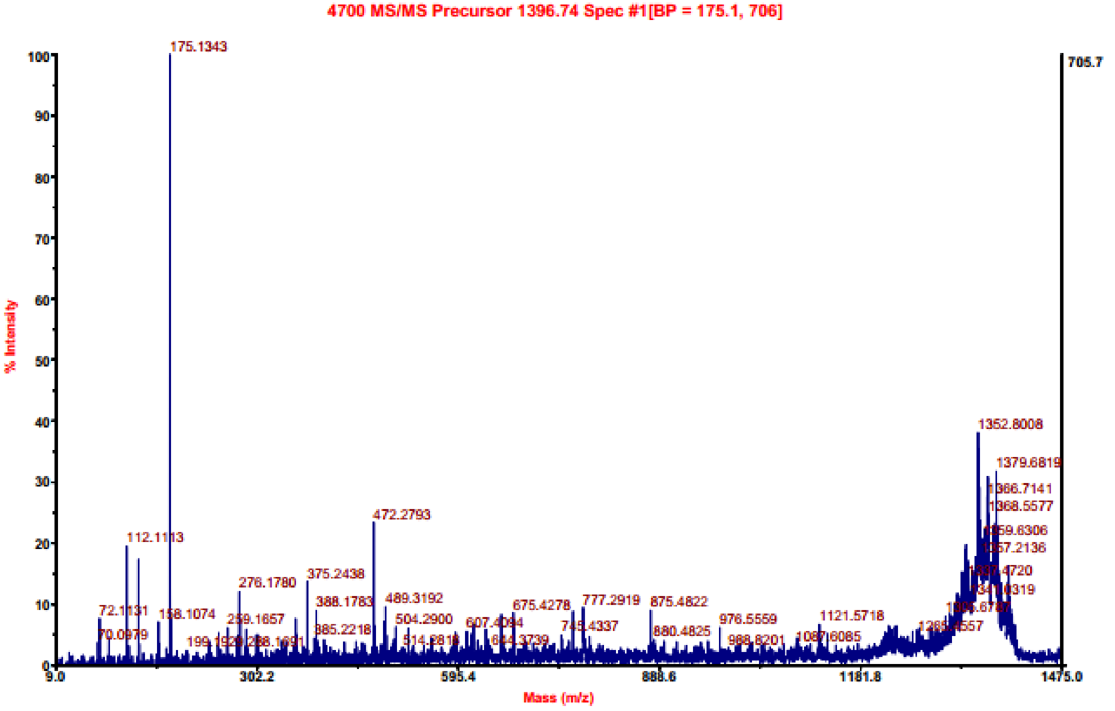

Spot 56

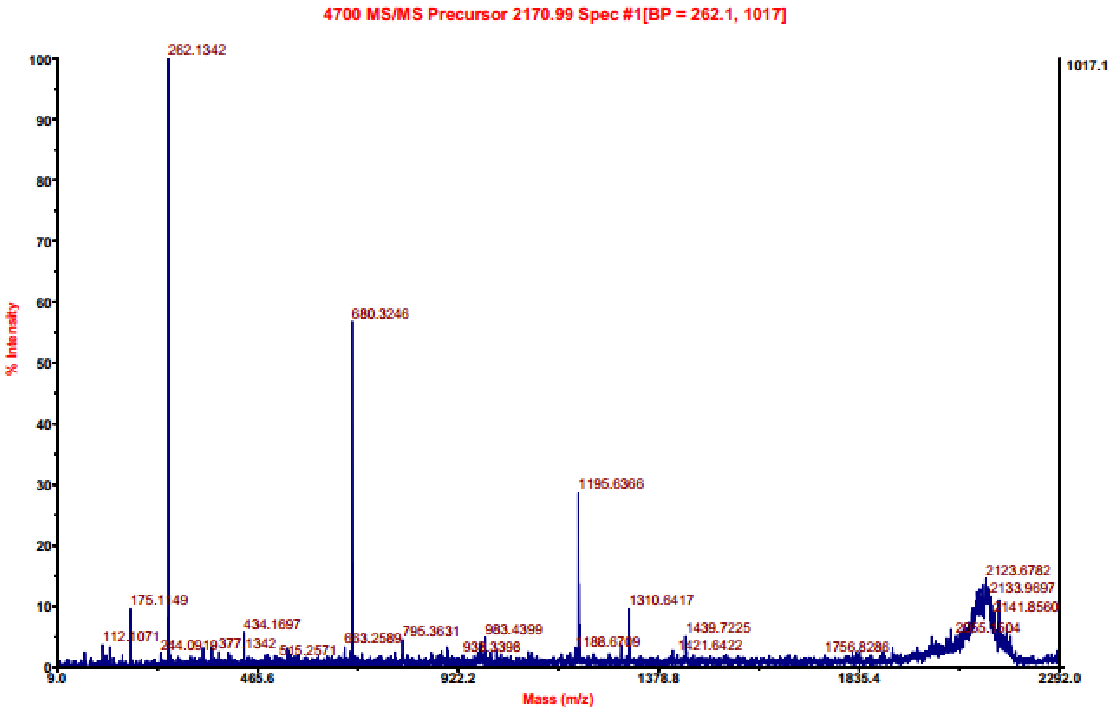

Spot 57

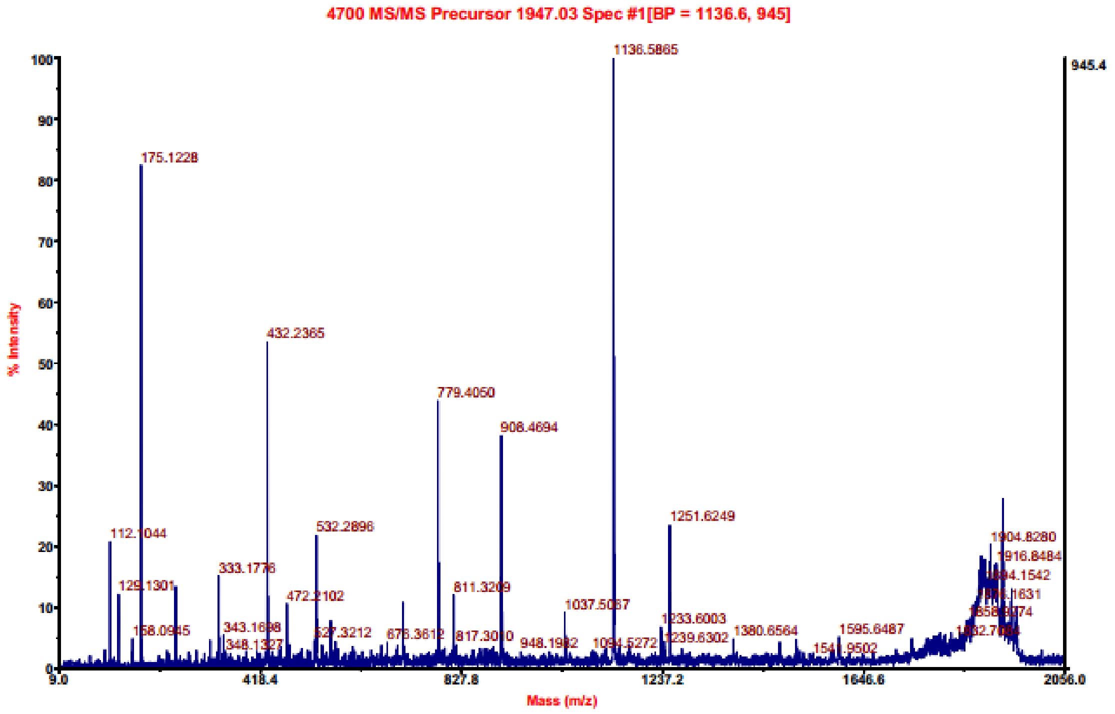

Spot 59

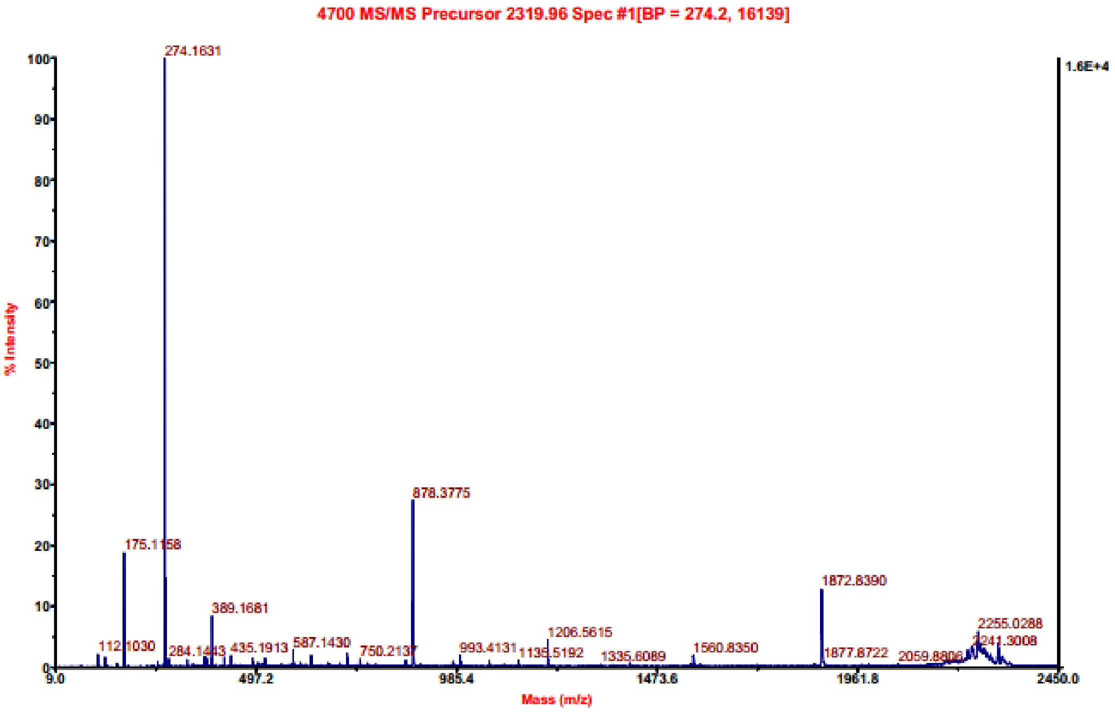

Spot 60

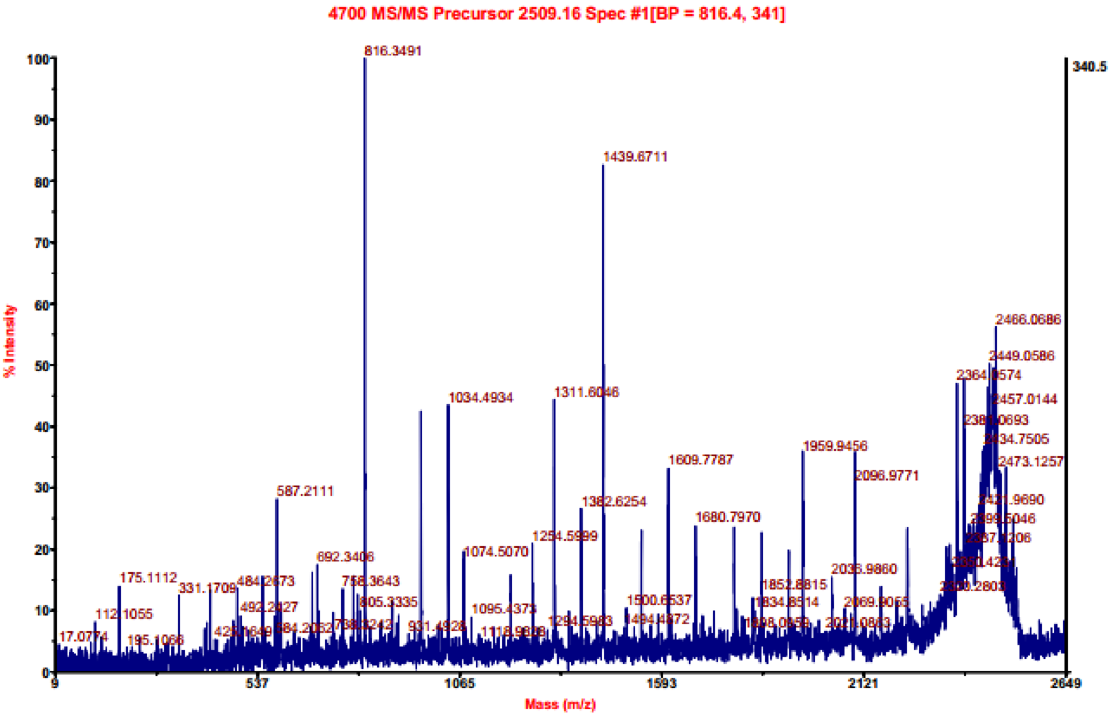

Spot 64

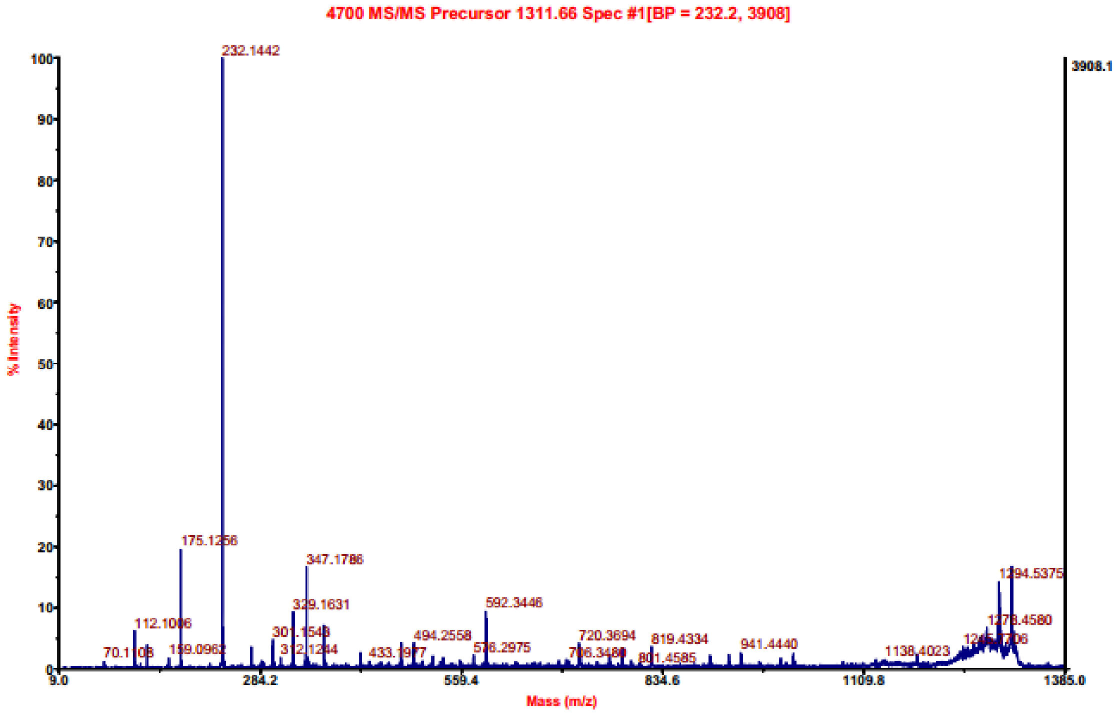

Spot 77

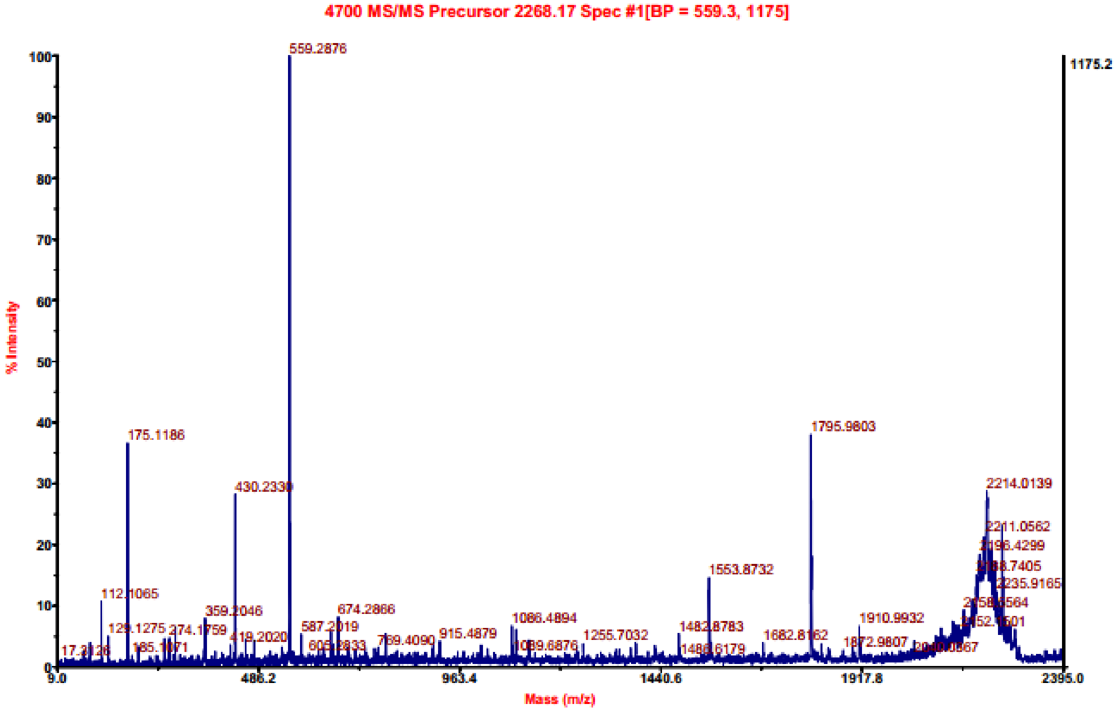

Spot 78

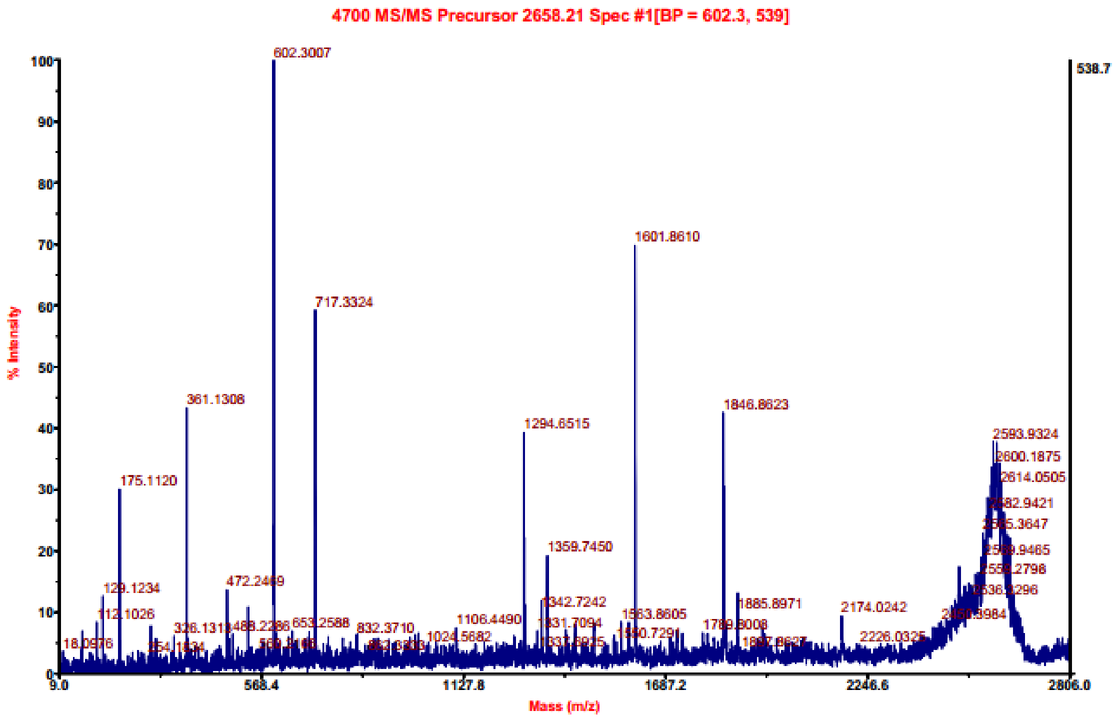

Spot 82

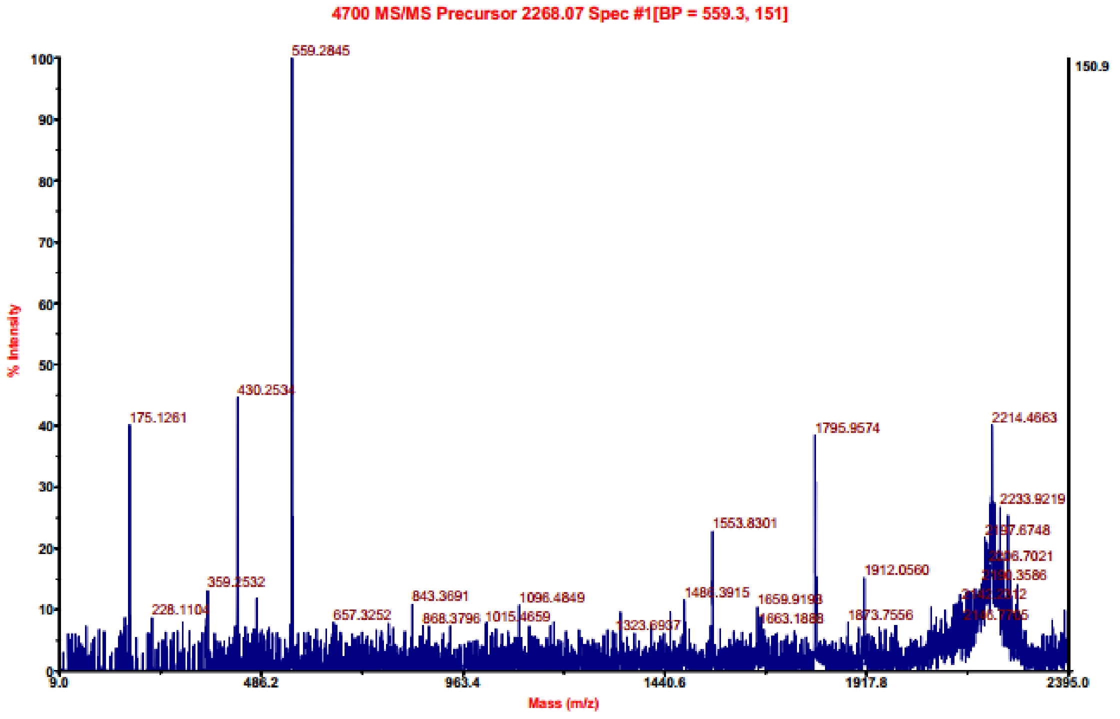

Spot 85

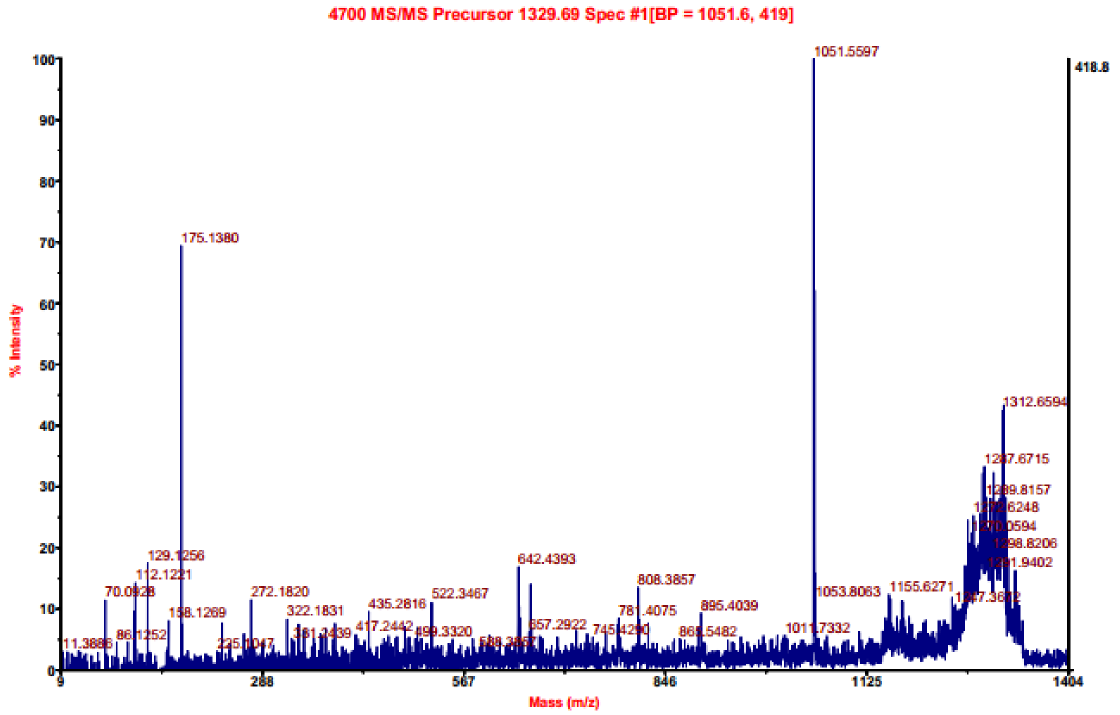

Spot 88

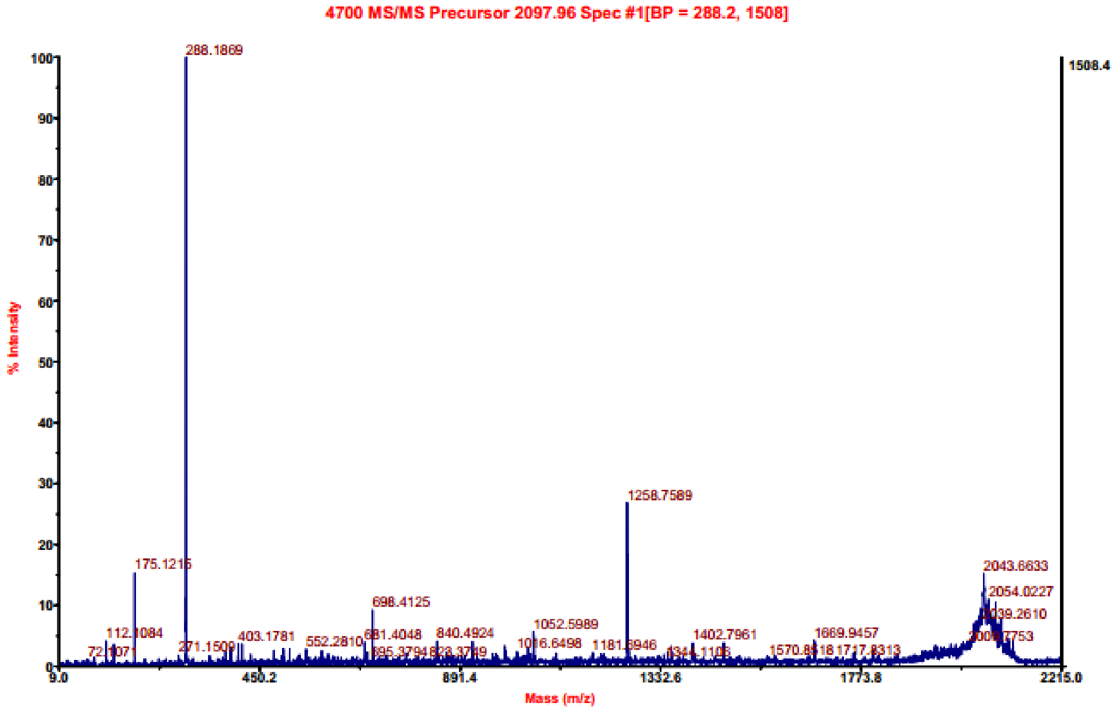

Spot 92

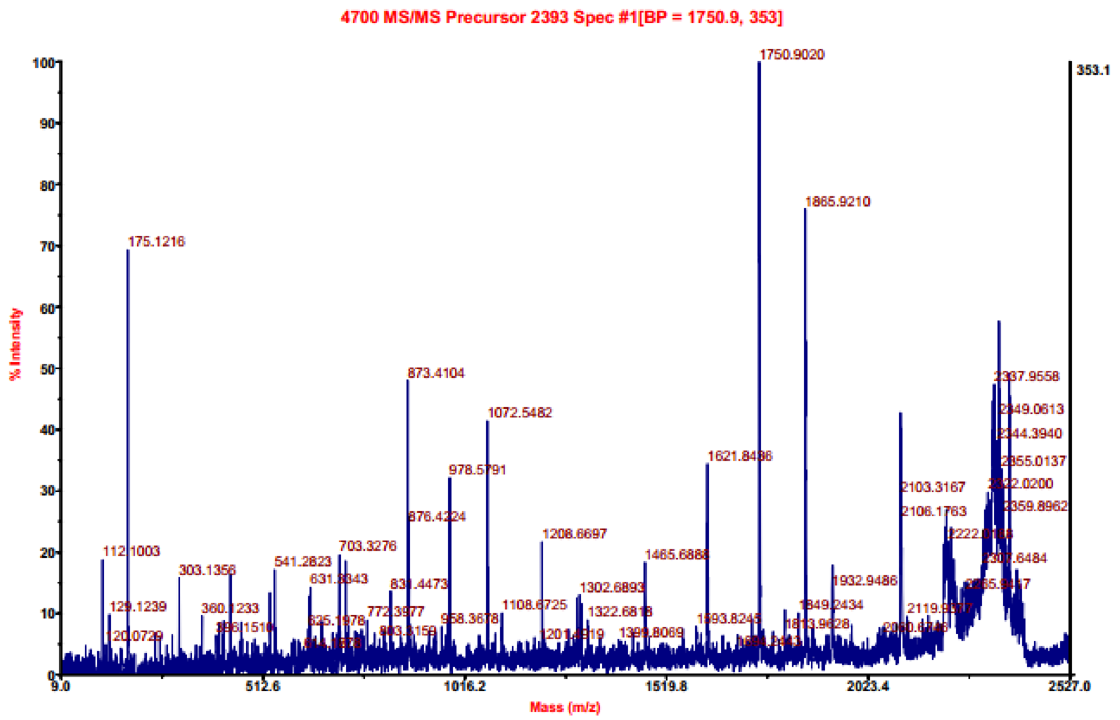

Spot 110

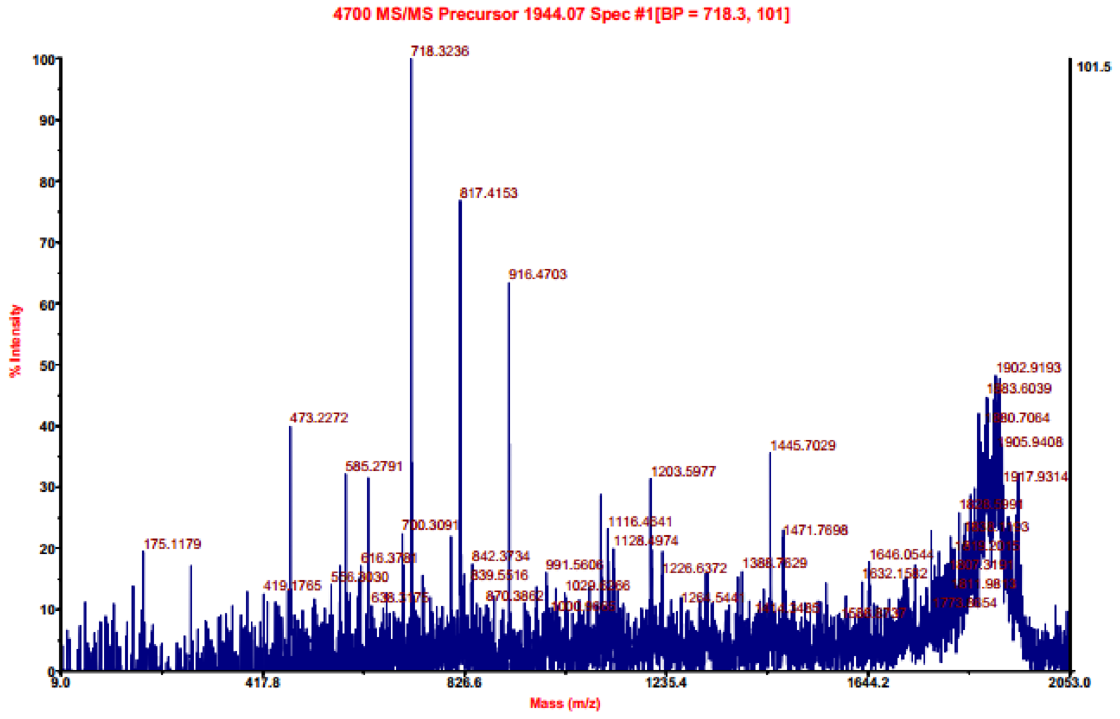

Spot 123

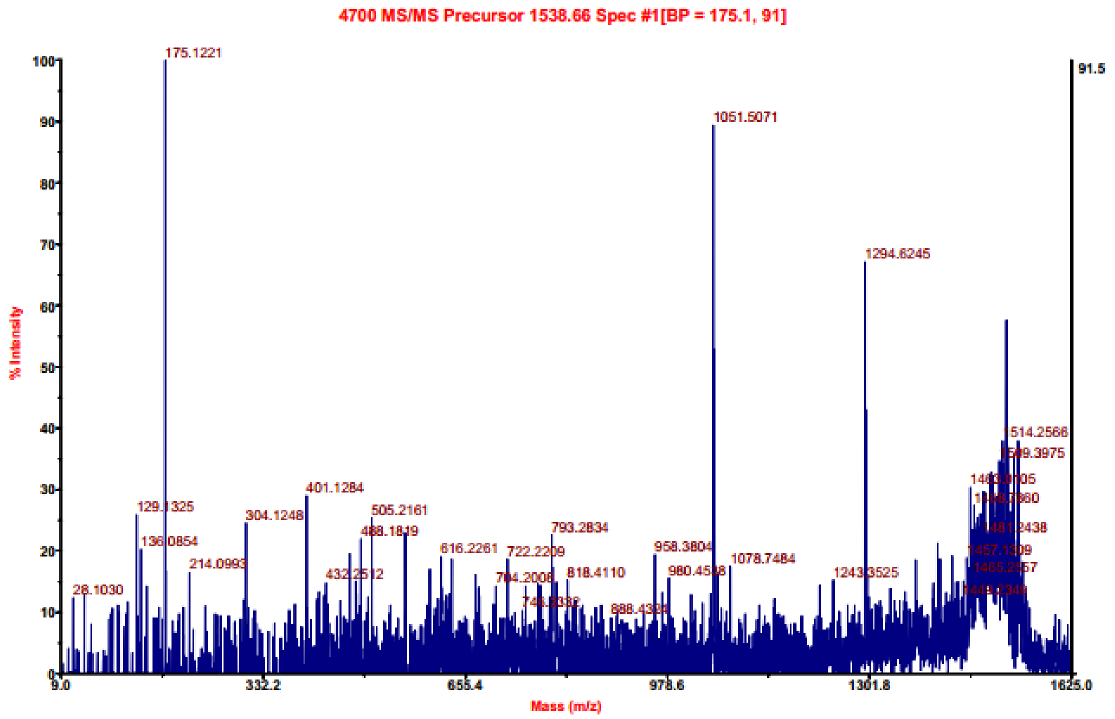

Spot 124

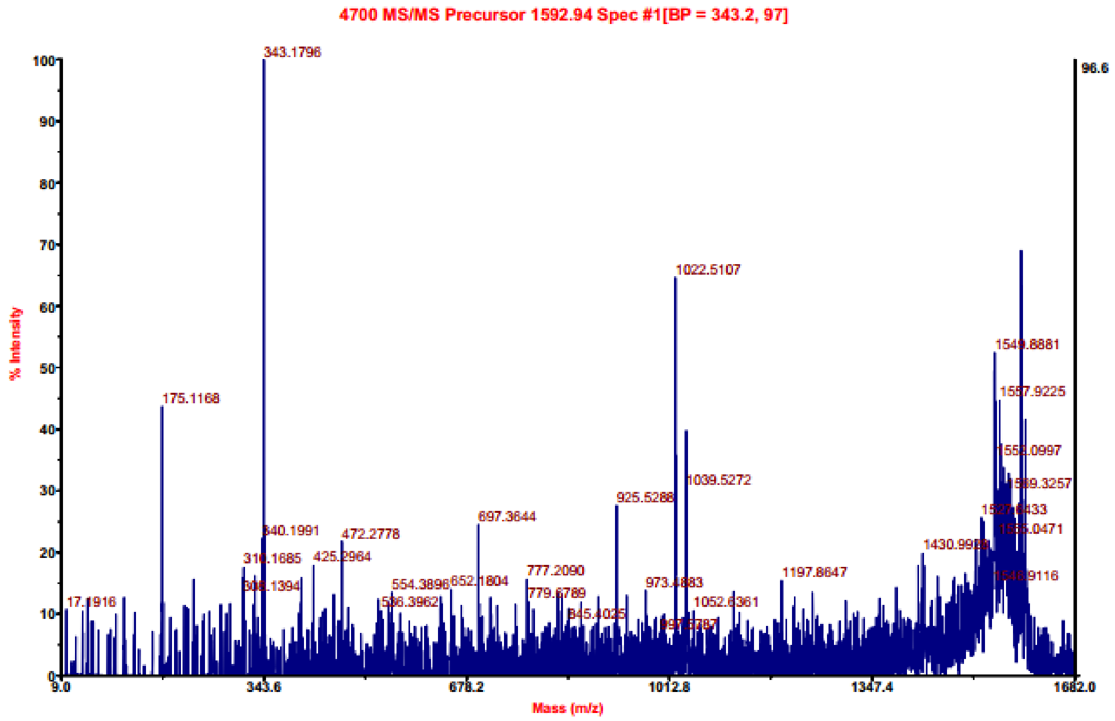

Spot 126

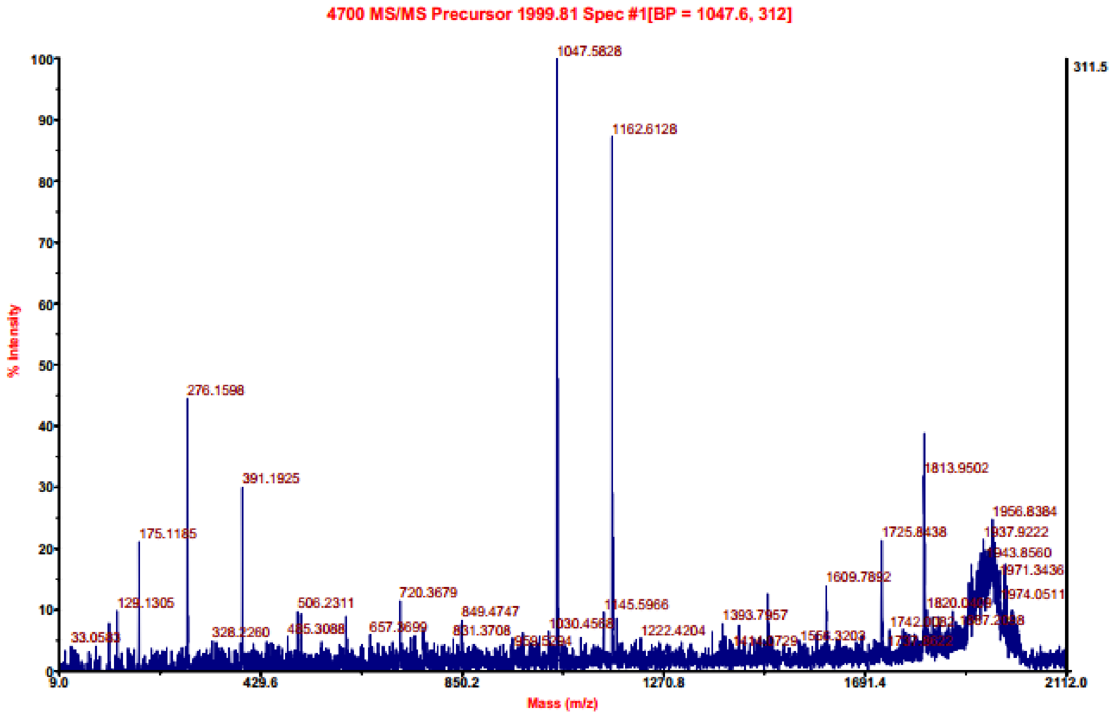

Spot 134

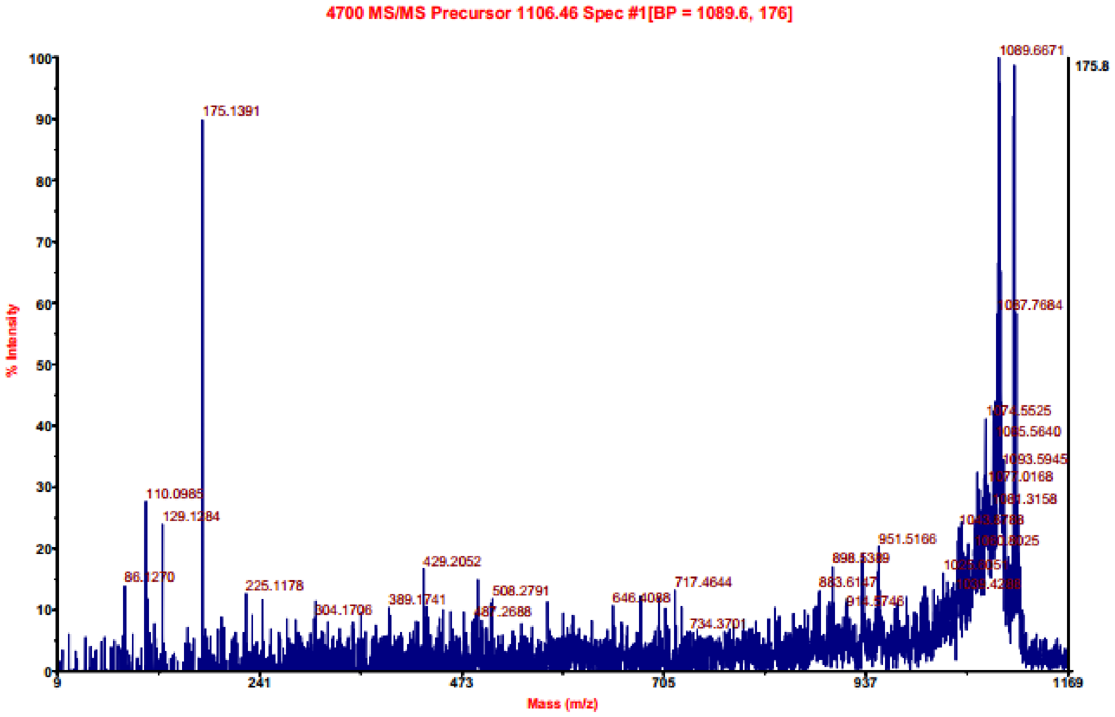

Spot 136

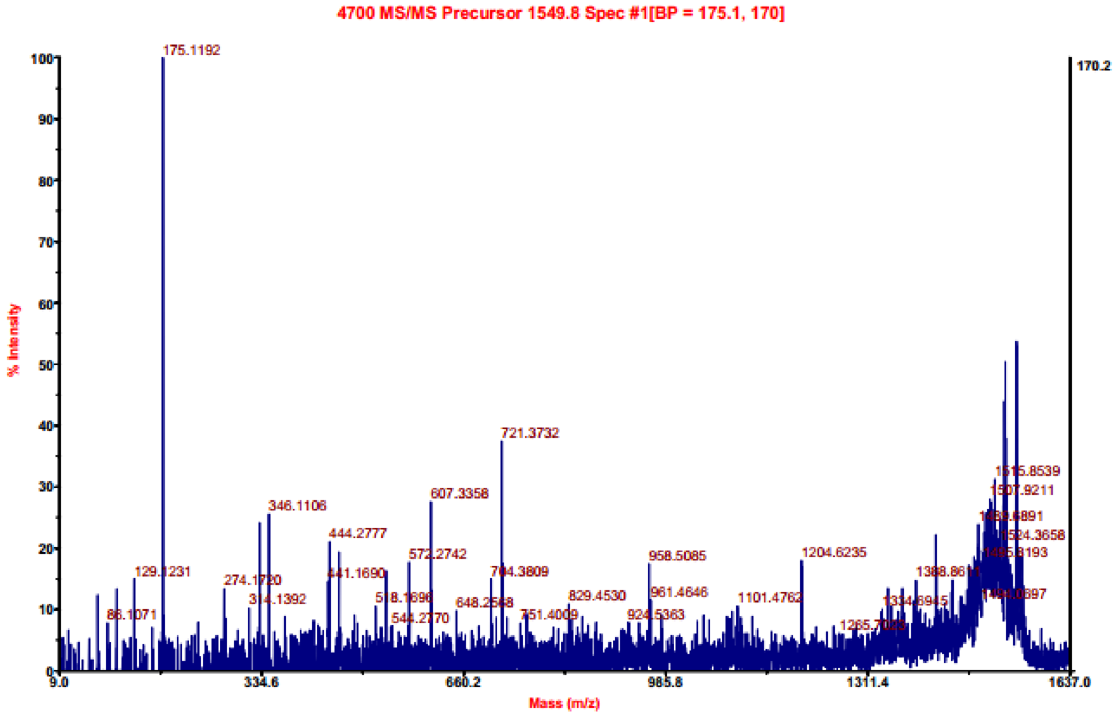

Spot 137

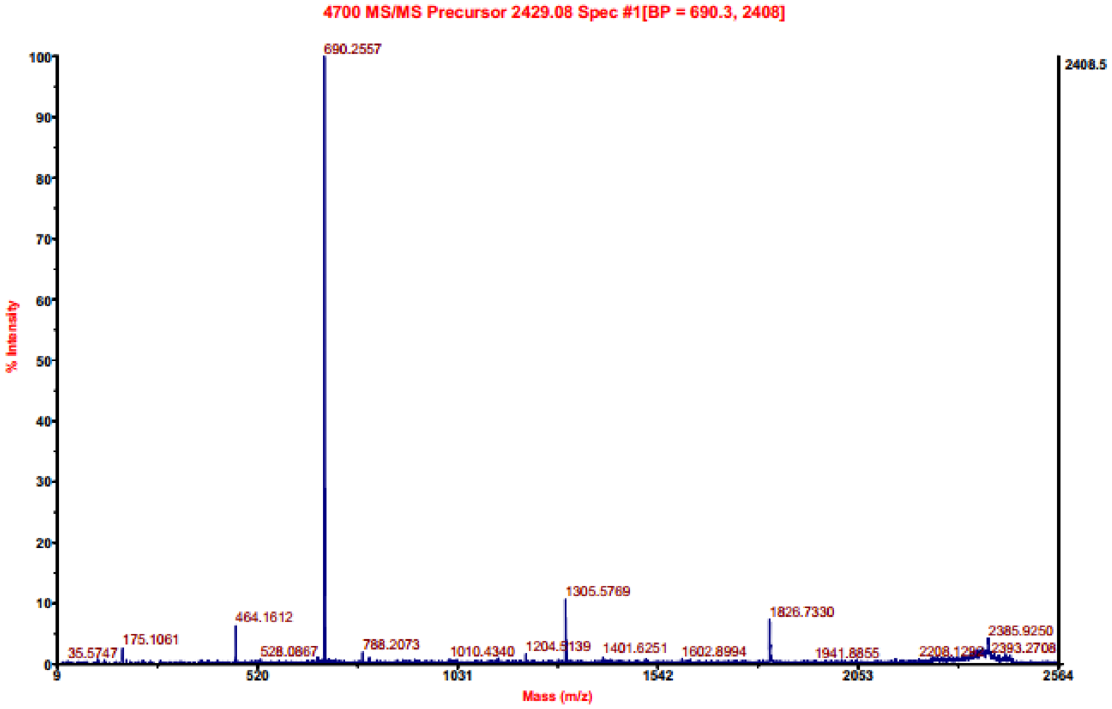

Spot 141

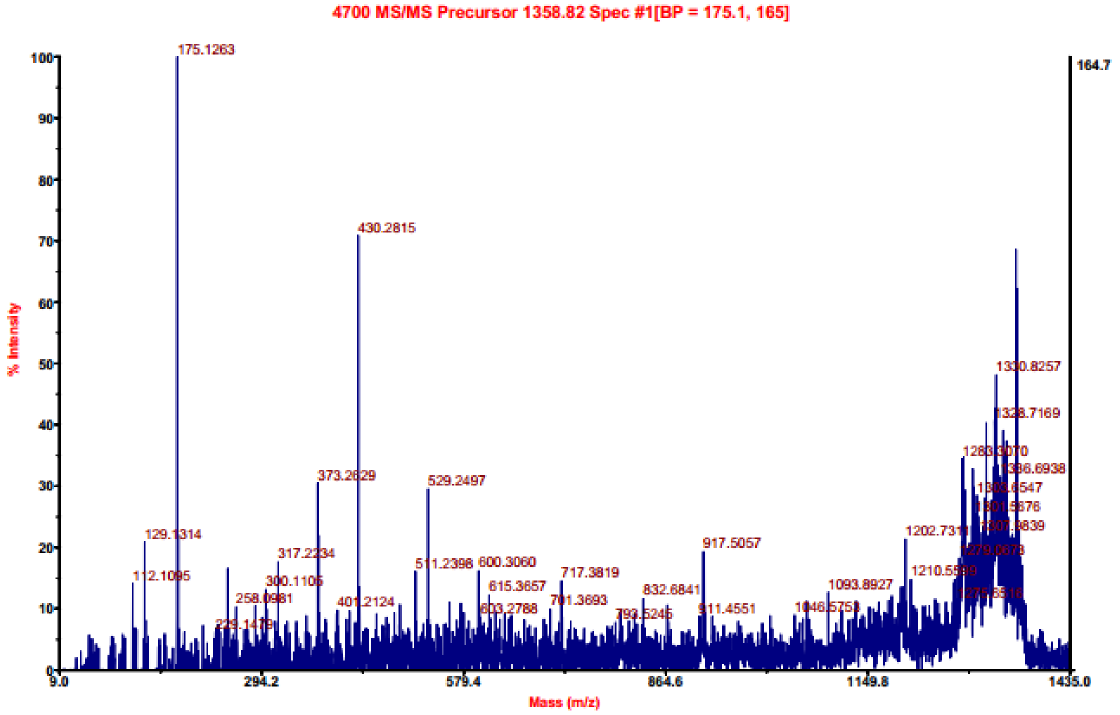

Supplementary Figure S8. | Spectra of single peptide.
