# Supplementary material for: Comparative Proteomic Analysis of Susceptible and Resistant Rice Plants during Early Infestation by Small Brown Planthopper
Source: Front Plant Sci. 2017 Oct 17;8:1744. doi: 10.3389/fpls.2017.01744 (PMC5651024; doi:10.3389/fpls.2017.01744)
Supplement: Supplementary file 16 [file Image9.PDF]

Spot 3(Protein of unknown function DUF538)

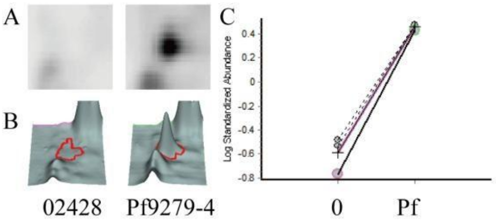

Spot 7(60S acidic ribosomal protein P0, P0 60S)

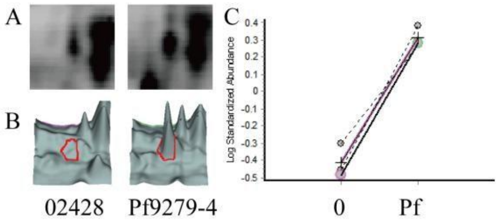

Spot 9(23 kDa polypeptide of photosystem II PsbP)

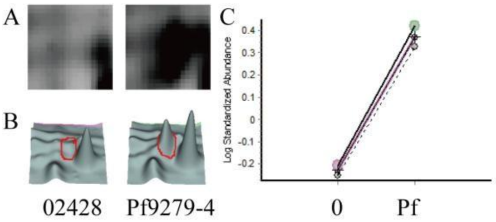

Spot 10(glutathione peroxidase, GSH-Px)

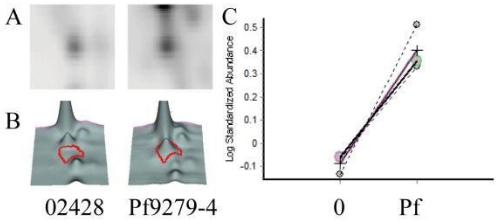

Spot 11(60 kDa chaperonin alpha subunit)

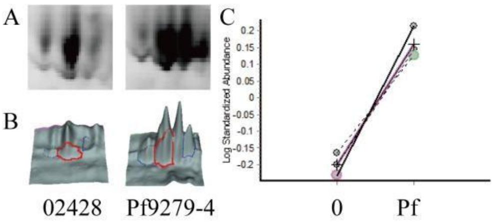

Spot 13(Ribulose bisphosphate carboxylase activase)

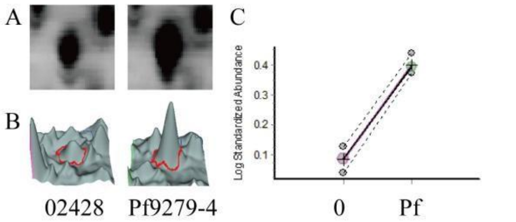

Spot 14(fructose-bisphospate aldolase, ALD)

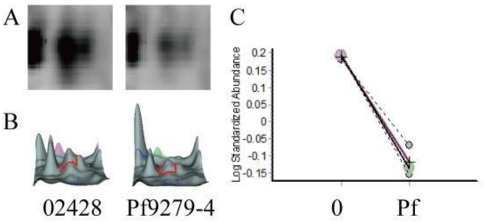

Spot 15(Tubulin beta chain)

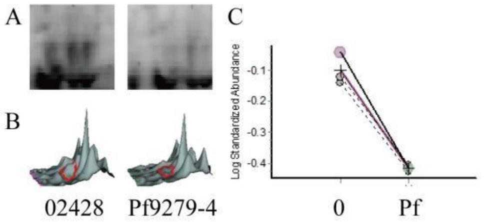

Spot 16(Probable aldo-keto reductase 2, AKR)

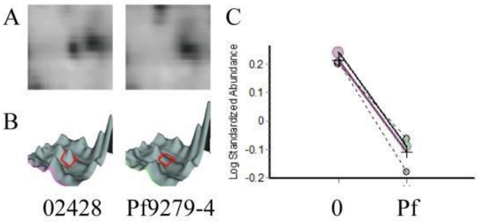

Spot 17(UDP-arabinopyranose mutase 1)

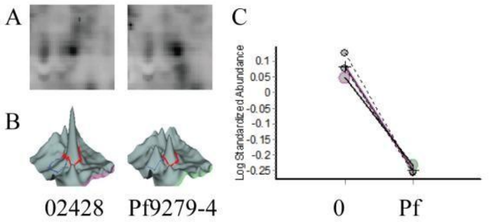

Spot 18(Aldehyde dehydrogenase, ALDH)

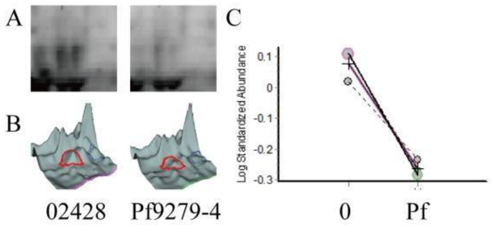

Spot 19(RNase S-like protein)

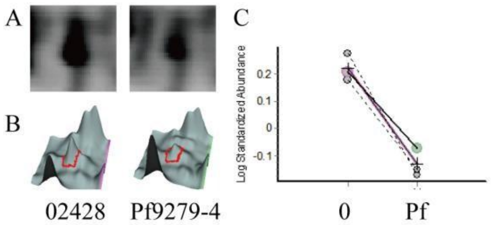

Spot 20(Heat shock 70 kDa protein, HSP70)

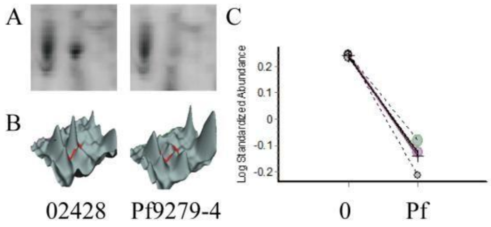

Spot 21(Flavodoxin/nitric oxide synthase)

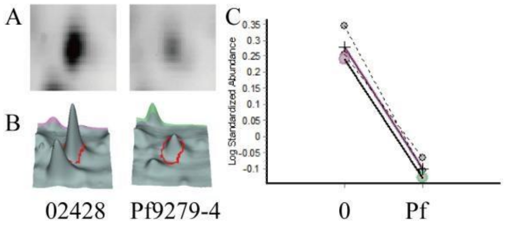

Spot 22(Vacuolar-type H+-ATPase subunit A1)

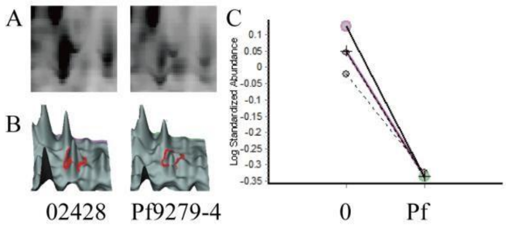

Spot 23(Aspartate-semialdehyde dehydrogenase)

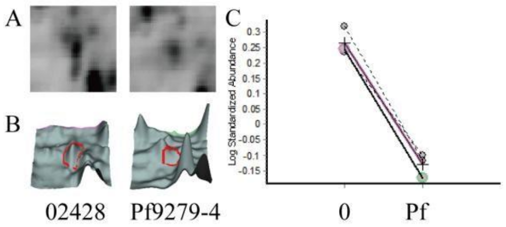

Spot 24(Chloroplast inorganic pyrophosphatase, SIP)

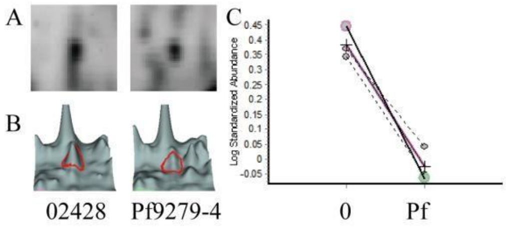

Spot 25(ATP synthase β chain)

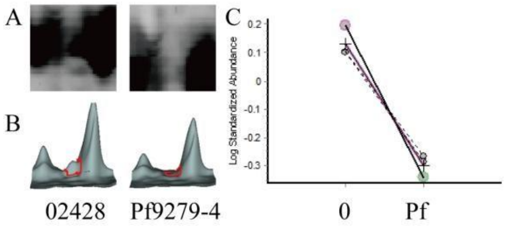

Spot 26(enolase)

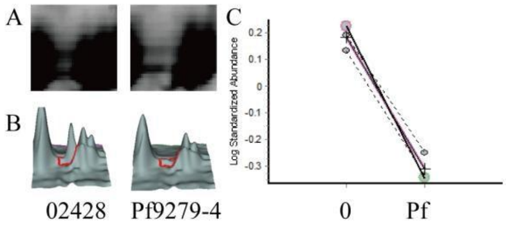

Spot 27(chlorophyll A-B binding protein, CAB)

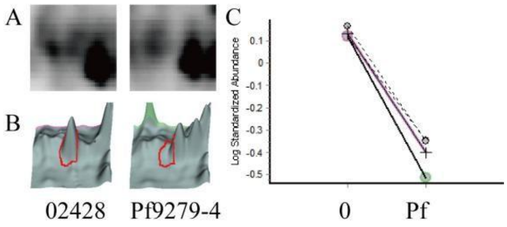

Spot 28(ribulose bisphosphate carboxylase large chain, RuBisCo)

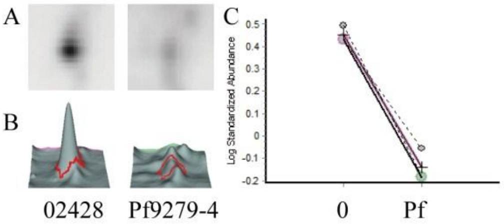

Spot 29(ATP synthase γ chain)

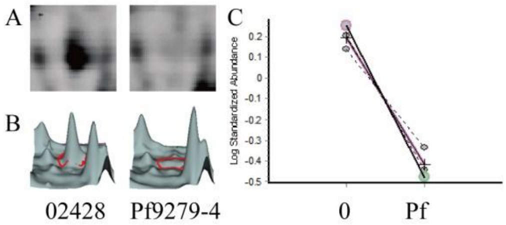

Spot 31(Asparaginyl-tRNA synthetase, KS)

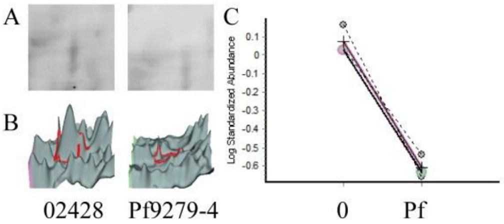

Spot 32(CROC-1-like protein)

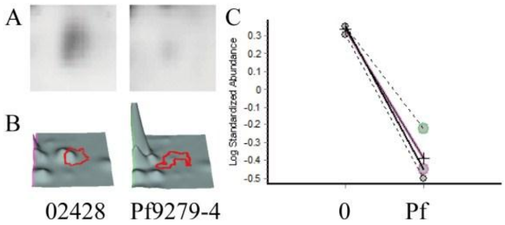

Spot 33(salt stress root protein RS1, RS1)

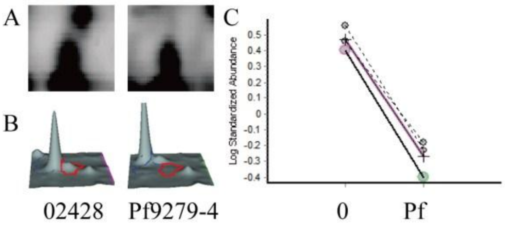

Spot 34(salt stress root protein RS1, RS1)

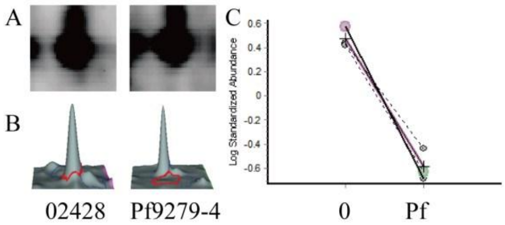

Spot 35(Protein disulfide-isomerase, PDI)

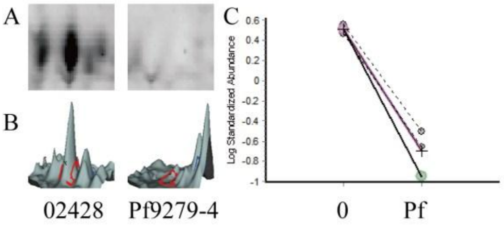

Spot 4(Thioredoxin F-type 2(TRX-F2))

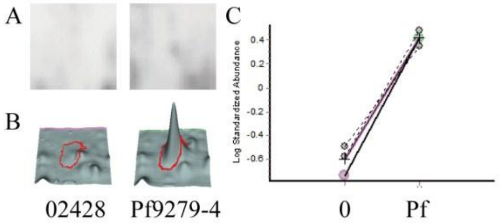

Spot 12(Reversibly glycosylated polypeptide)

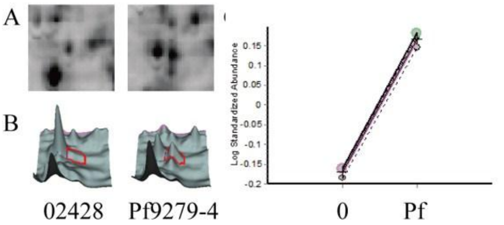

Spot 145(UTP--glucose-1-phosphate uridylyltransferase)

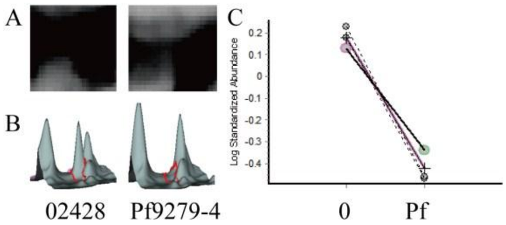

Spot 102(cysteine synthase)

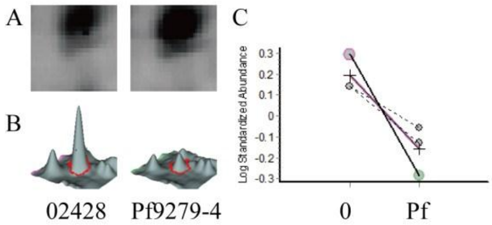

Spot 37(Photosystem II oxygen-evolving complex protein 1, OEE1)

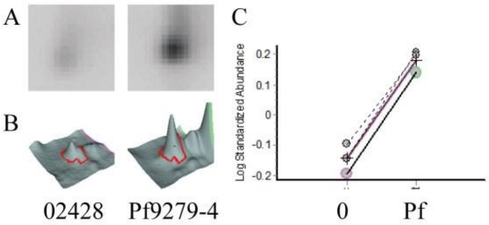

Spot 38(salt stress root protein RS1, RS1)

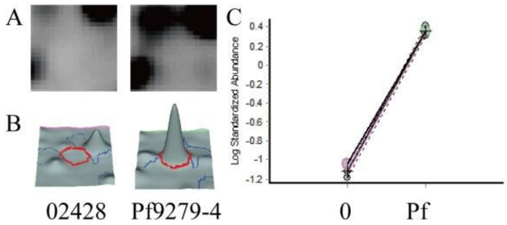

Spot 39(Mannose-binding lectin)

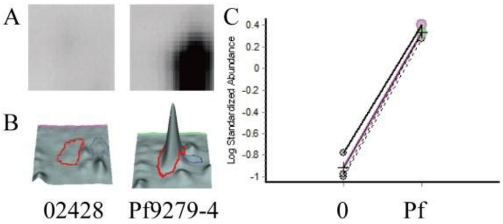

Spot 40(Photosystem II oxygen-evolving complex protein 1, OEE1)

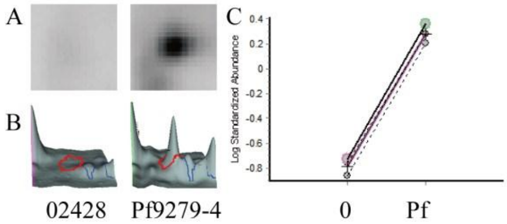

Spot 41(salt stress root protein RS1, RS1)

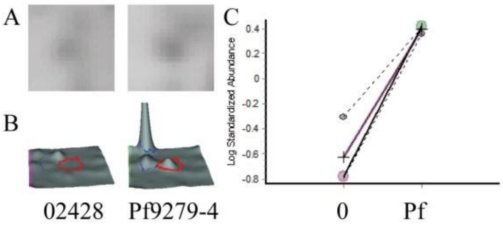

Spot 42(eukaryotic translation initiation factor 5A, ETIF5A)

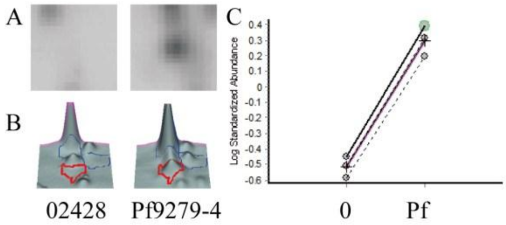

Spot 43(Putative Photosystem I reaction center subunit IV, PS I subunit IV)

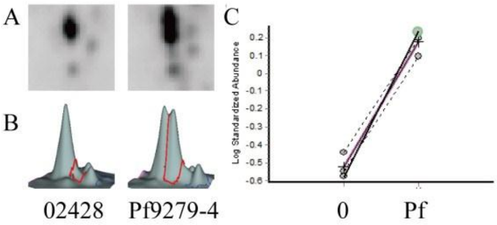

Spot 44(Flavodoxin/nitric oxide synthase)

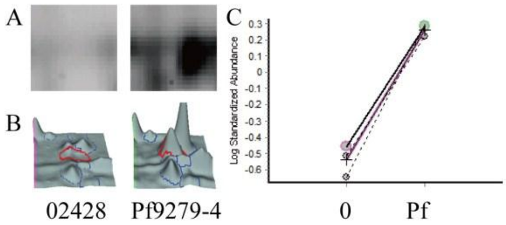

Spot 45(NADP-isocitrate dehydrogenase,IDH)

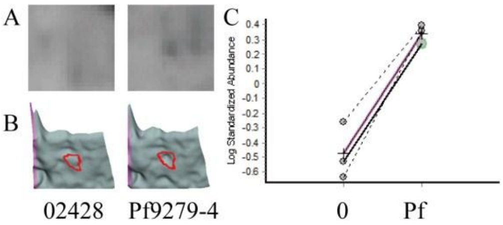

Spot46(ribulose bisphosphate carboxylase large chain, RuBisCo)

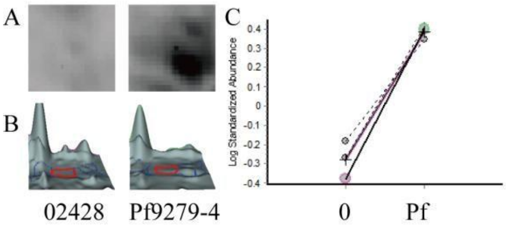

Spot 47(ribulose bisphosphate carboxylase large chain, RuBisCo)

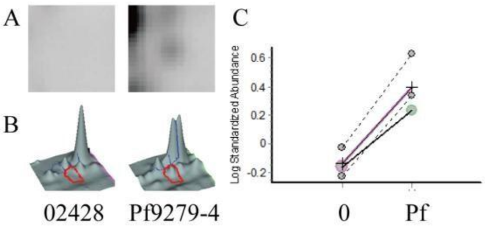

Spot 48(Fructokinase, pfkB family, PFK)

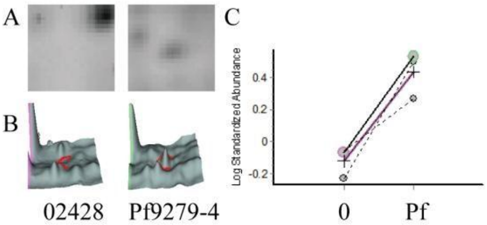

Spot 49(G-box binding factor,14-3-3)

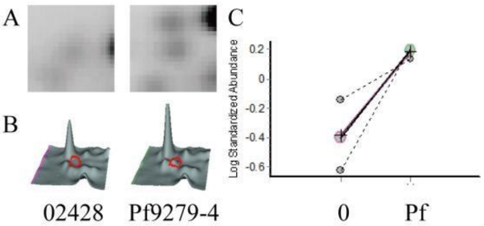

Spot 50(50S ribosomal protein L12)

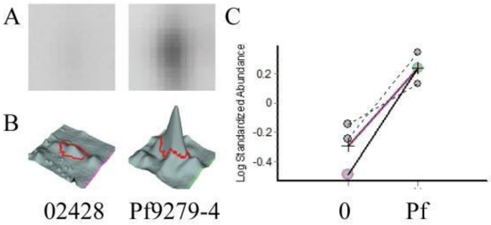

Spot 51(chlorophyll A-B binding protein, CAB)

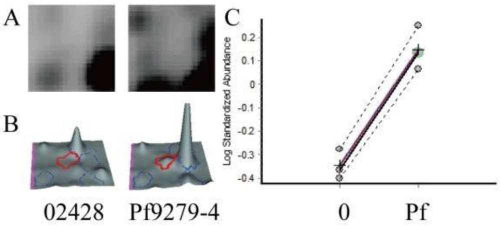

Spot 52(Nod factor binding lectin-nucleotide phosphohydrolase)

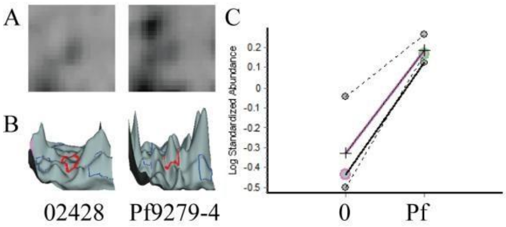

Spot 103(Chitinase III-like protein)

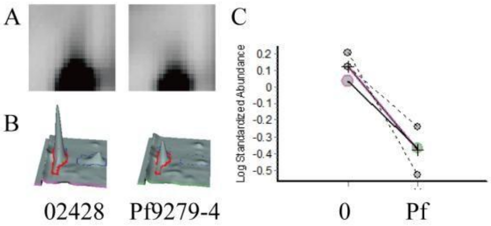

Spot 54(glutathione S-transferase, GSTs)

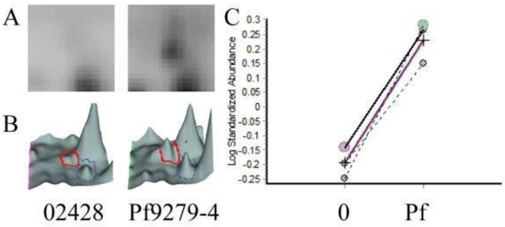

Spot 55(UDP-glucose pyrophosphorylase)

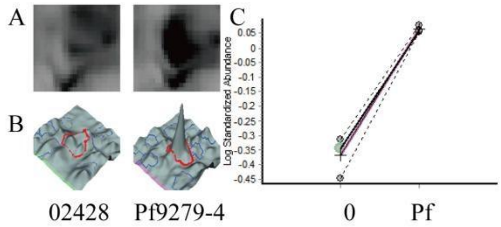

Spot 56(glyceraldehyde-3-phosphate dehydrogenase, GAPDH)

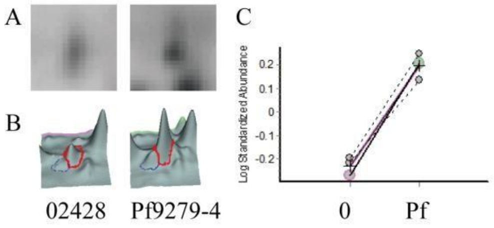

Spot 57(Alcohol dehydrogenase, ADH)

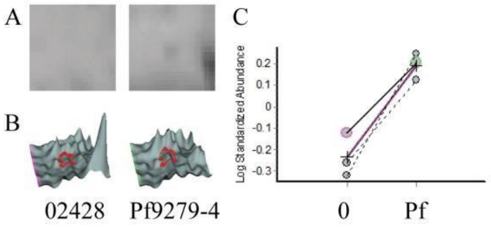

Spot 58(chlorophyll A-B binding protein, CAB)

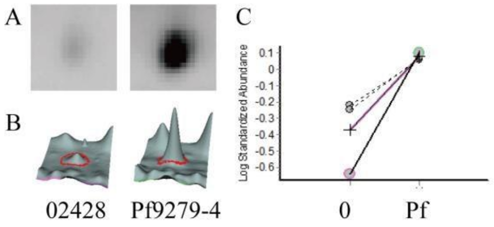

Spot 59(acyltransferase)

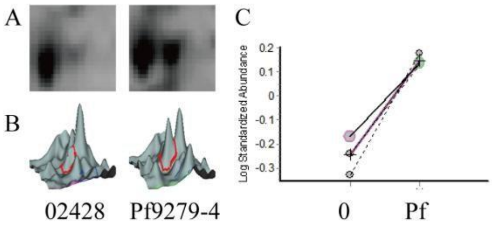

Spot 60(ABA/WDS induced protein)

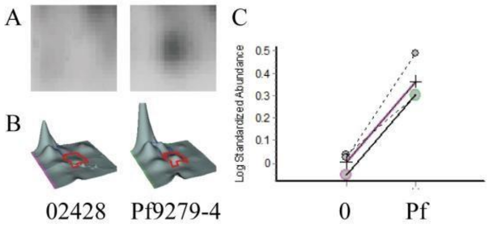

Spot 104(Chitinase III-like protein)

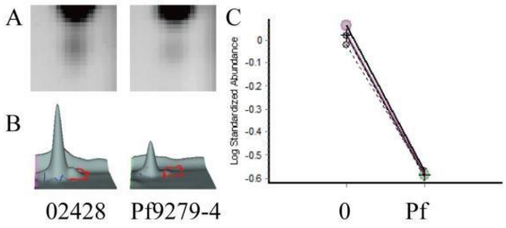

Spot 62(chlorophyll A-B binding protein, CAB)

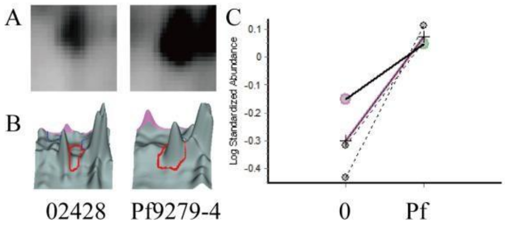

Spot 63(ABA/WDS induced protein)

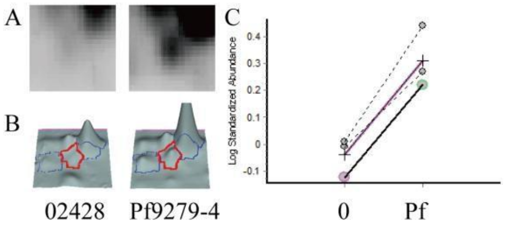

Spot 64(chlorophyll A-B binding protein, CAB)

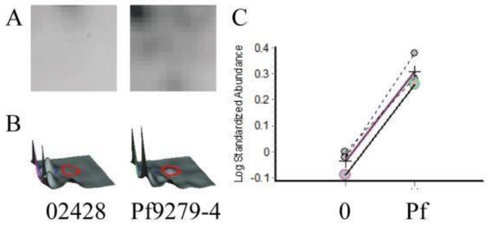

Spot 65(glyceraldehyde-3-phosphate dehydrogenase,GAPDH)

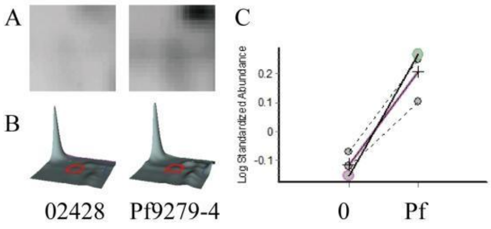

Spot 66(Magnesium-chelatase subunit ChlI)

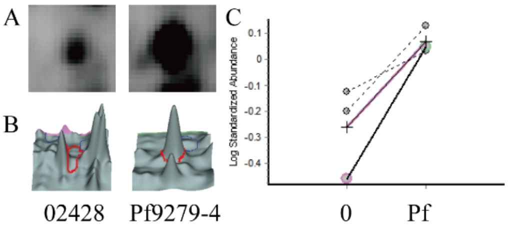

Spot 67(Ricin B-related lectin domain containing protein)

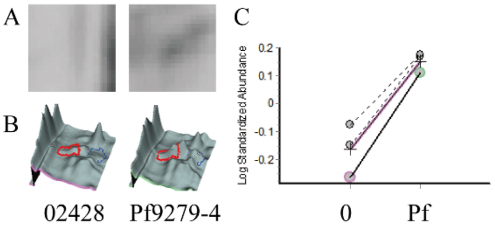

Spot 68(elongation factor Tu)

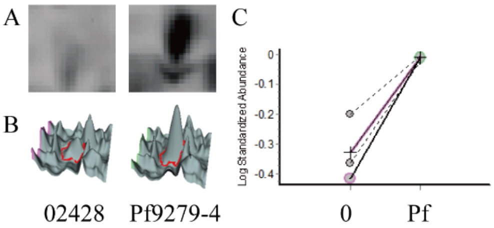

Spot 69(60 kDa chaperonin alpha subunit)

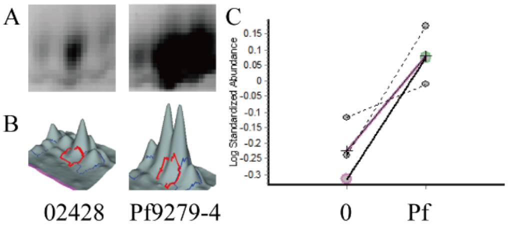

Spot 70(ribulose-1,5-bisphosphate carboxylase/ oxygenase large subunit)

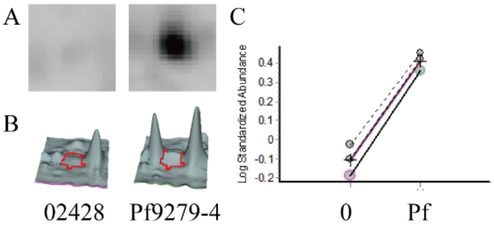

Spot 43(Putative Photosystem I reaction center subunit IV, PS I subunit IV)

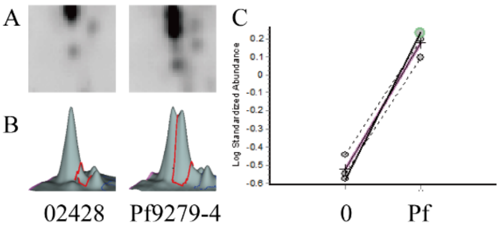

Spot 72(ribulose bisphosphate carboxylase large chain, RuBisCo)

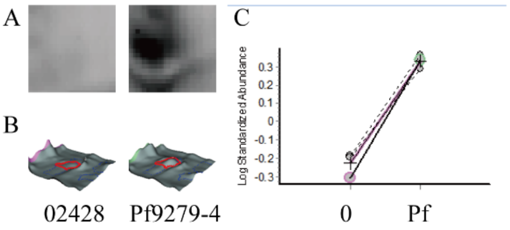

Spot 73(ribulose-1,5-bisphosphate carboxylase/ oxygenase large subunit)

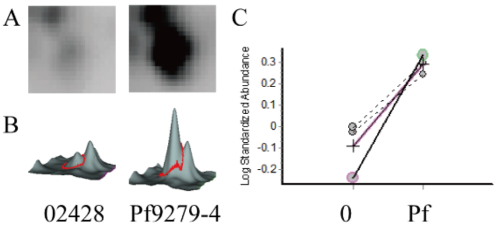

Spot 74(Flavodoxin/nitric oxide synthase)

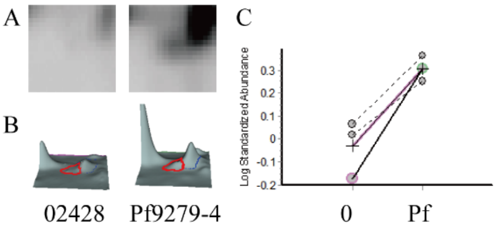

Spot 75(Mannose-binding lectin domain containing protein, MBL)

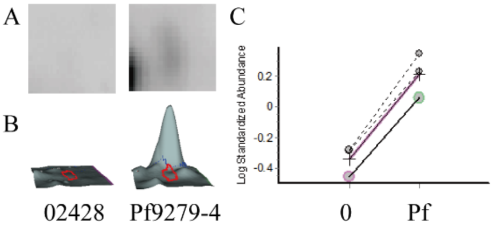

Spot 76(Mannose-binding lectin domain containing protein, MBL)

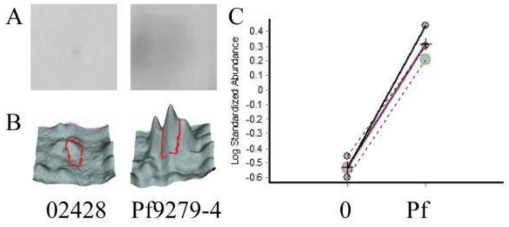

Spot 77(5-methyltetrahydropteroyltriglutamate-homocysteine methyltransferase)

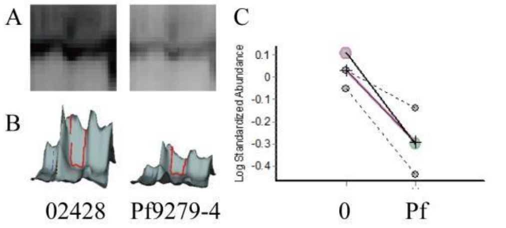

Spot 78(70 kDa heat shock protein, HSP70)

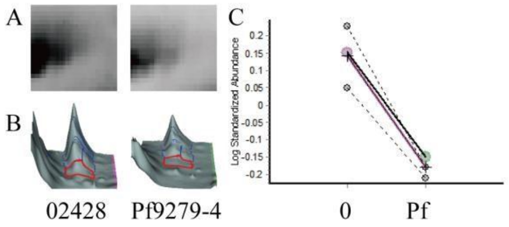

Spot 79(2,3-bisphosphoglycerate-independent phosphoglycerate mutase, PGAM)

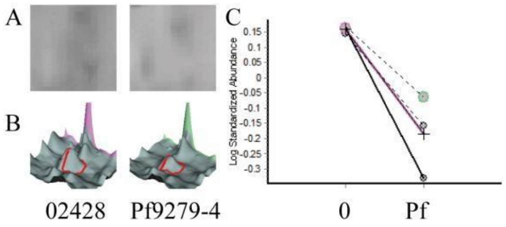

Spot 80(heat shock protein 81-1, HSP81-1)

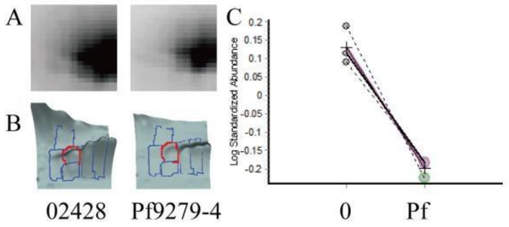

Spot 81(cysteine synthase)

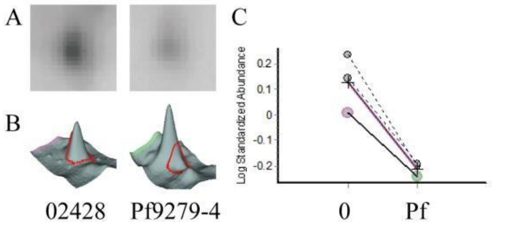

Spot 82(5-methyltetrahydropteroyltriglutamate-homocysteine methyltransferase)

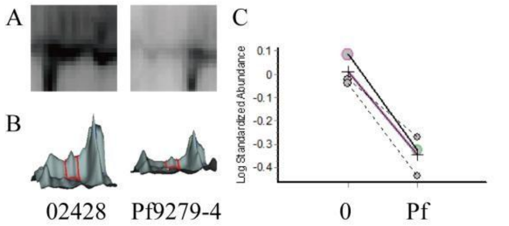

Spot 83(Ribulose bisphosphate carboxylase/ oxygenase activase, RuBisCO)

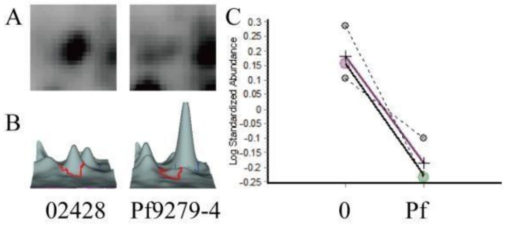

Spot 84(ATP synthase subunit alpha)

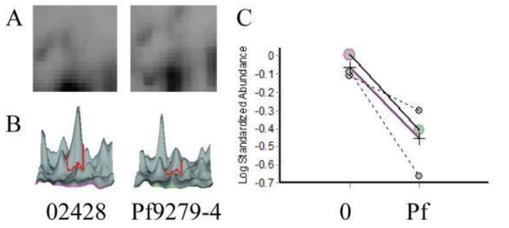

Spot 85(phospholipase D)

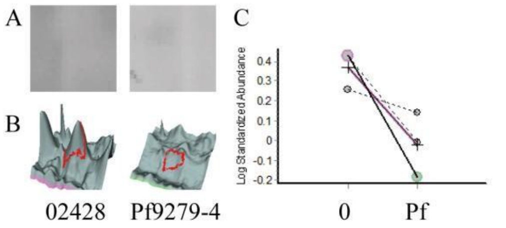

Spot 86(putative beta-1,3-glucanase)

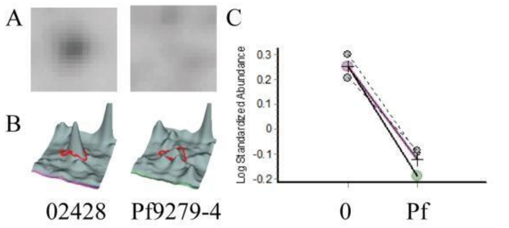

Spot 87(Quinone oxidoreductase-like protein)

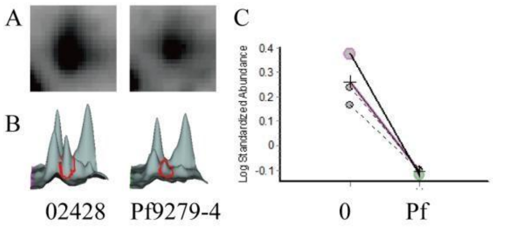

Spot 88(Elongation factor G)

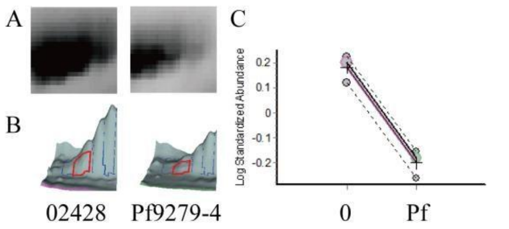

Spot 89(glycosyl hydrolase)

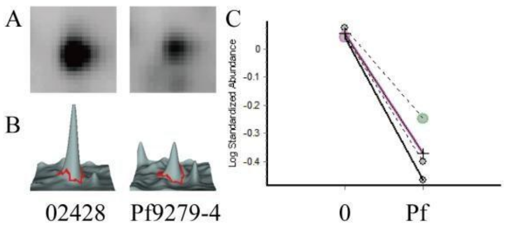

Spot 91(transketolase, TK)

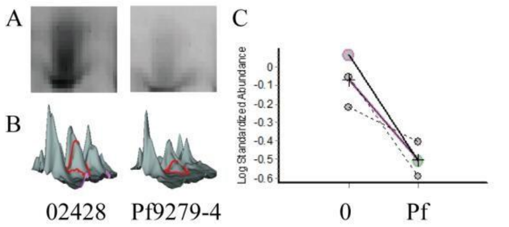

Spot 92(malate dehydrogenase, AEDH)

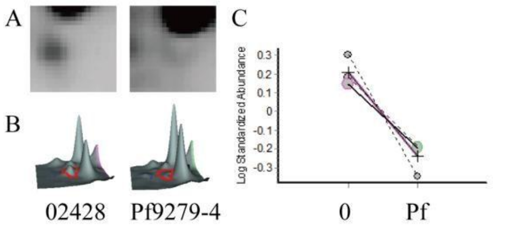

Spot 93(ATP synthase β chain)

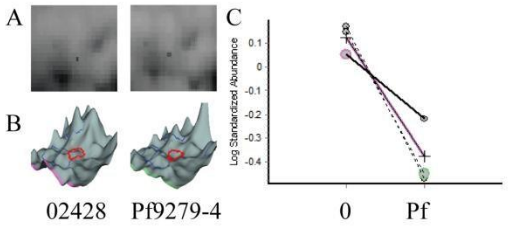

Spot 94(Oligopeptidase A-like)

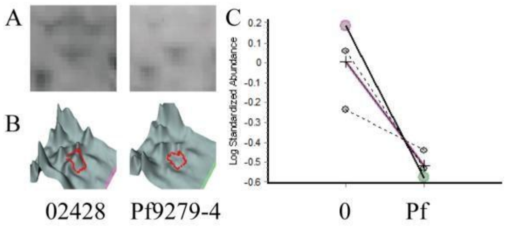

Spot 95(Chitinase III-like protein)

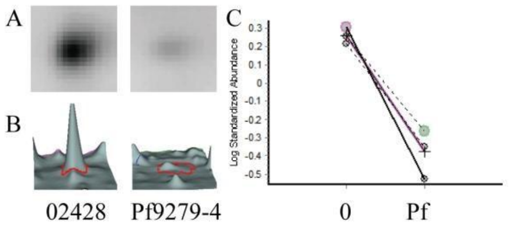

Spot 96(Formate dehydrogenase, FDH)

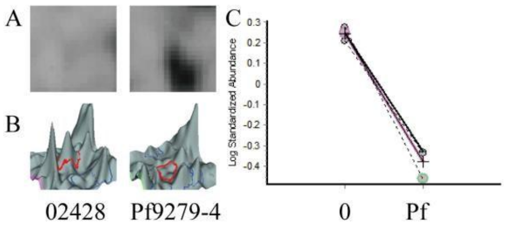

Spot 97(Protein disulfide isomerase-like 1-1, PDI)

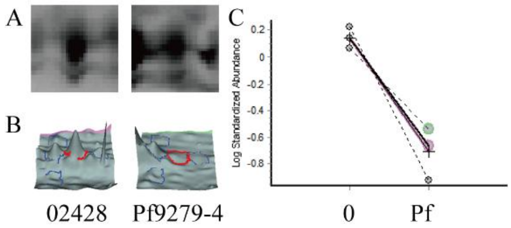

Spot 98(Heat shock protein 81-3, HSP81-3)

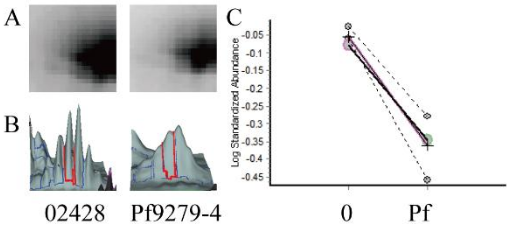

Spot 99(heat shock protein 81-1, HSP81-1)

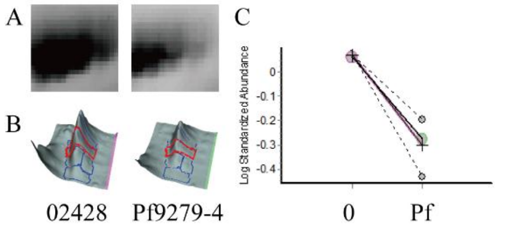

Spot 100(ATP synthase β chain)

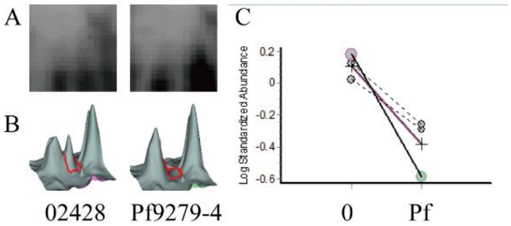

Spot 139(chlorophyll A-B binding protein, CAB)

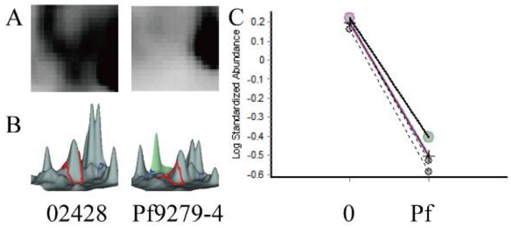

Spot 141(ATP synthase γ chain)

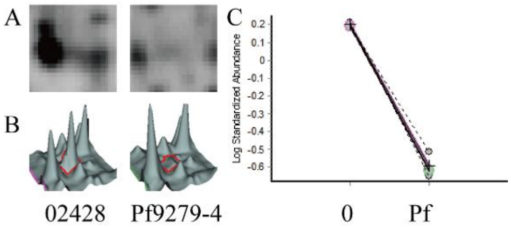

Spot 142(Soluble inorganic pyrophosphatase, SIP)

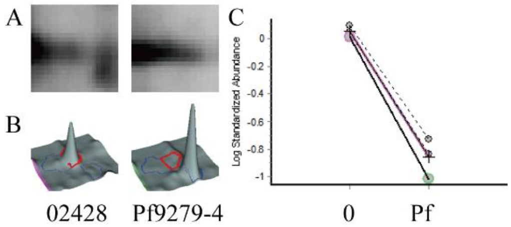

Spot 143(Alpha/beta hydrolase fold-3 domain containing protein, NYC3)

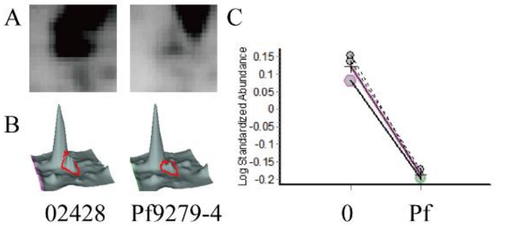

Spot 144(chlorophyll A-B binding protein, CAB)

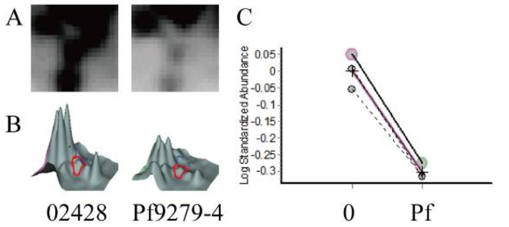

Spot 138(glycosyl hydrolase)

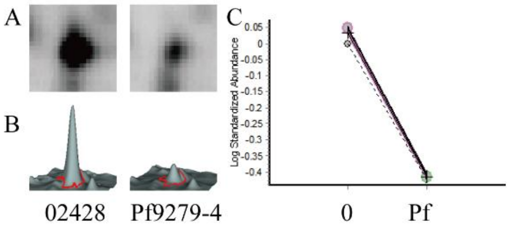

Spot 105(14-3-3-like protein GF14-D)

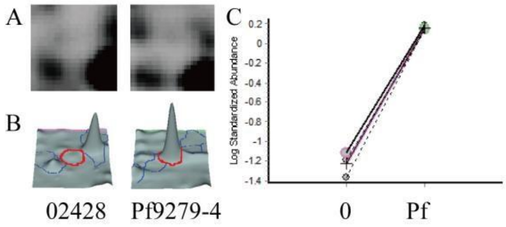

Spot 106(Mannose-binding lectin domain containing protein)

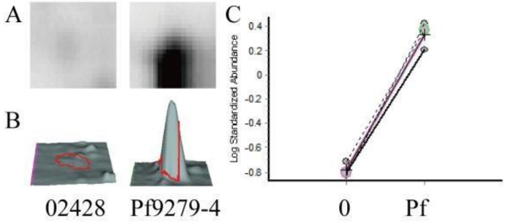

Spot 107(Glycin-rich RNA binding protein, GRP)

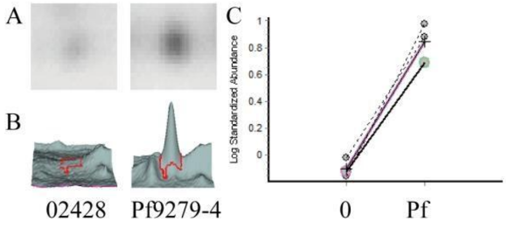

Spot 108(G-box binding factor, 14-3-3 protein)

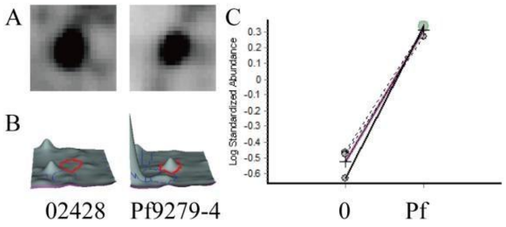

Spot 109(Apyrase)

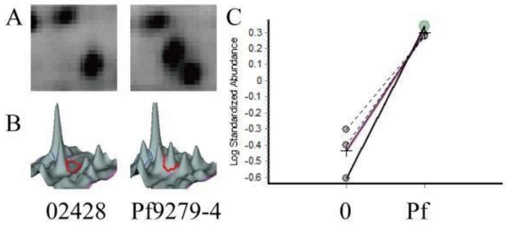

Spot 110(transcription factor BTF3, BTF3)

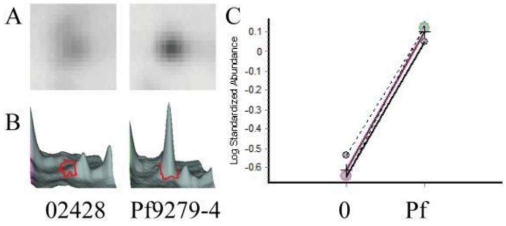

Spot 111(Photosystem I reaction center subunit IV, PS I subunit IV)

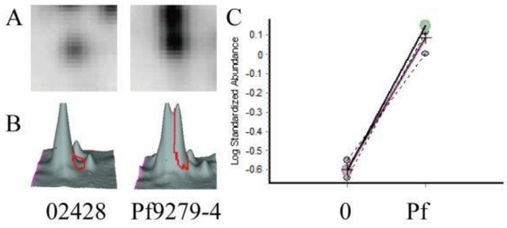

Spot 112(GDP-mannose 3,5-epimerase 2)

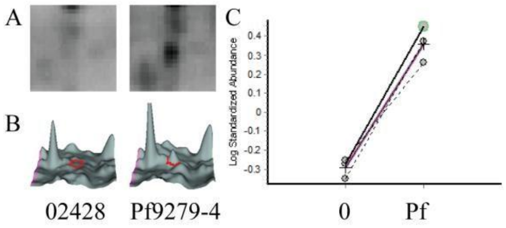

Spot 113(Protein disulfide isomerase-like 1-1, PDI)

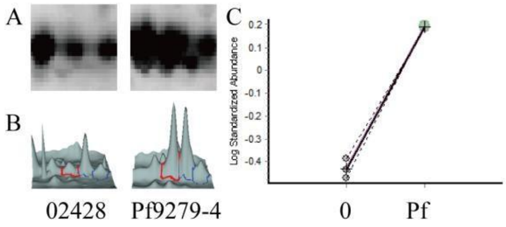

Spot 114(14-3-3)

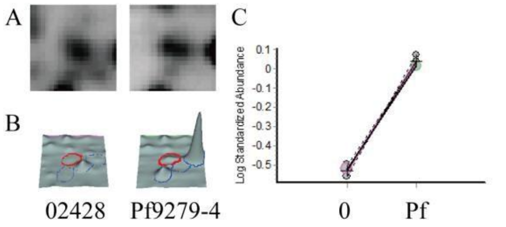

Spot 137(chlorophyll A-B binding protein, CAB)

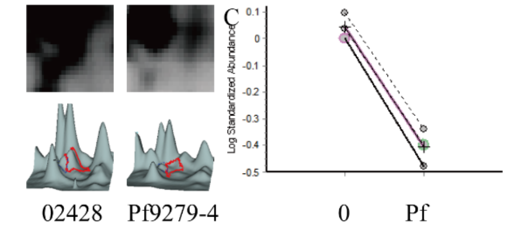

Spot 116(Chloroplast 23 kDa polypeptide of photosystem II)

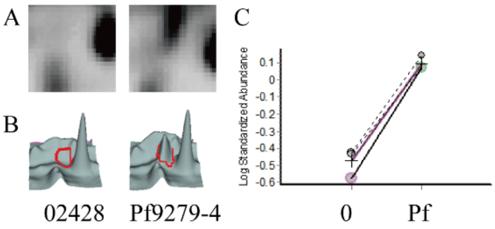

Spot 117(beta-amylase)

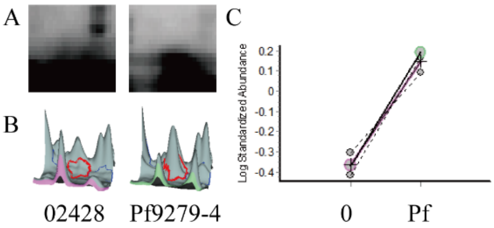

Spot 118(ferredoxin-NADP reductase, Ferredoxin)

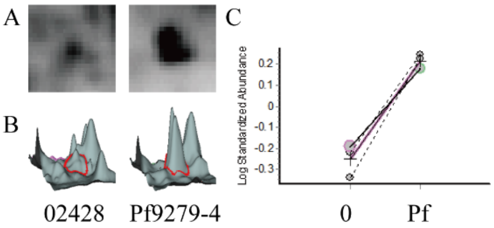

Spot 119(S-adenosylmethionine synthetase, SAM synthetase)

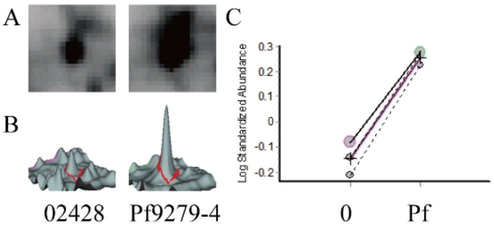

Spot 120(Isoflavone reductase-like protein, IRLs)

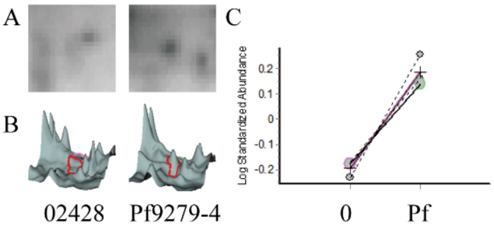

Spot 121(Glutamate-1-semialdehyde 2,1-aminomutase)

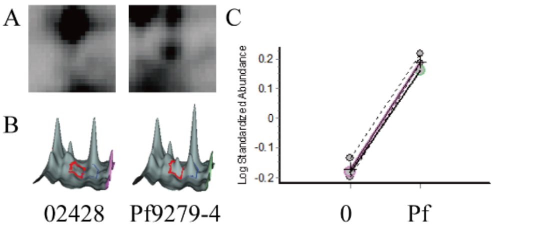

Spot 122(RuBisCO subunit binding-protein alpha)

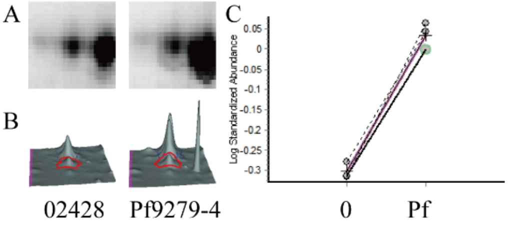

Spot 123(acetyl-CoA acetyltransferase)

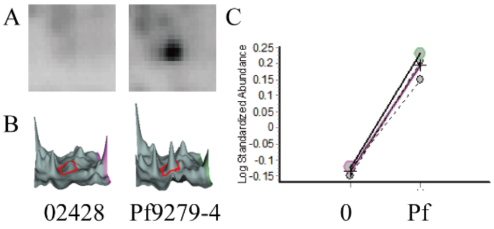

Spot 124(Esterase, SGNH hydrolase-type)

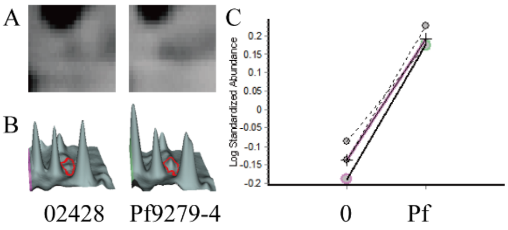

Spot 125(sucrose synthase)

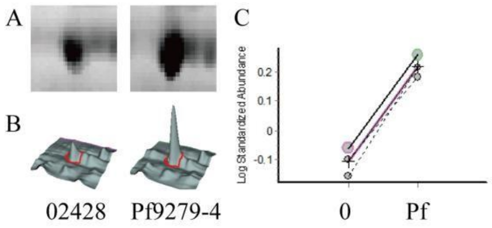

Spot 126(phosphoglycerate kinase protein, PGK)

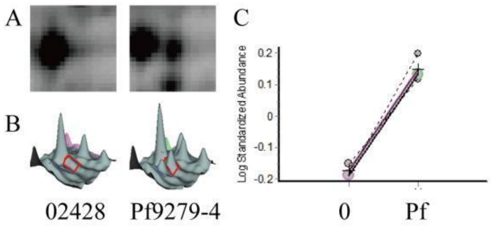

Spot 127(G-box binding factor, 14-3-3 protein)

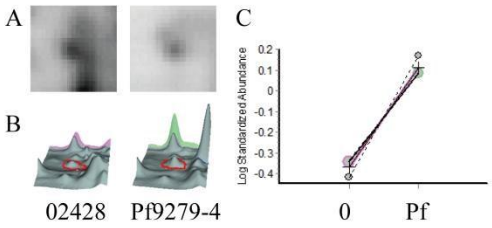

Spot 128(RuBisCO subunit binding-protein alpha subunit)

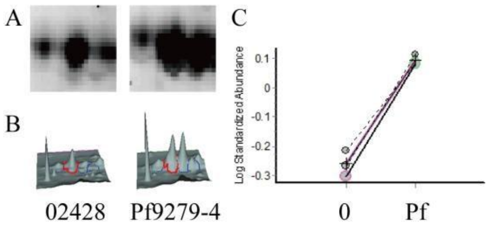

Spot 129(beta-amylase)

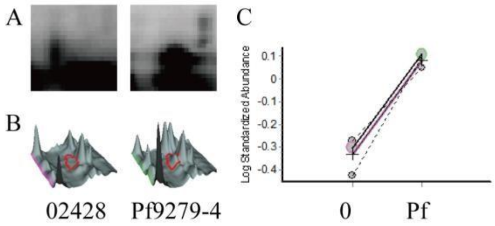

Spot 130(beta-amylase)

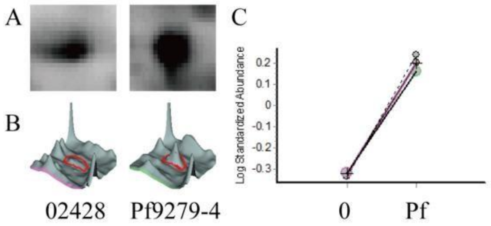

Spot 131(ATP synthase β chain)

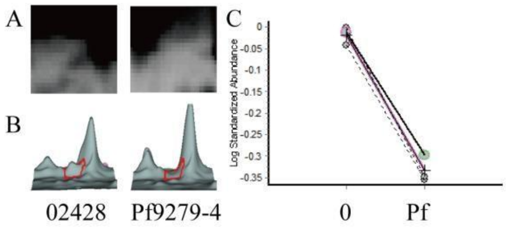

Spot 132(Ribulose bisphosphate carboxylase/ oxygenase activase, RuBisCO)

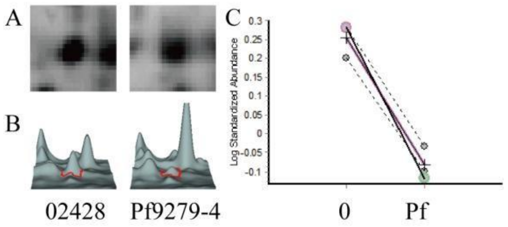

Spot 133(Flavodoxin/nitric oxide synthase domain containing protein.)

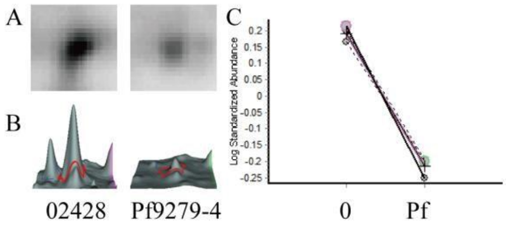

Spot 134(Asparaginyl-tRNA synthetase, KS)

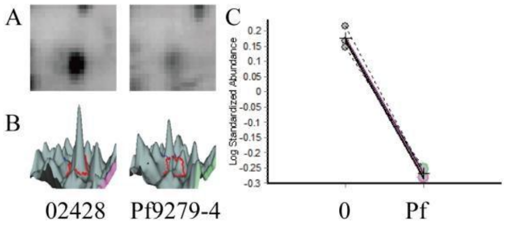

Spot 135(2,3-bisphosphoglycerate-independent phosphoglycerate mutase, PGAM)

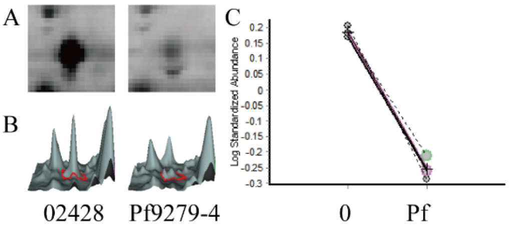

Spot 136(Isopentenyl pyrophosphate:dimethyllallyl pyrophosphate isomerase, IPI)

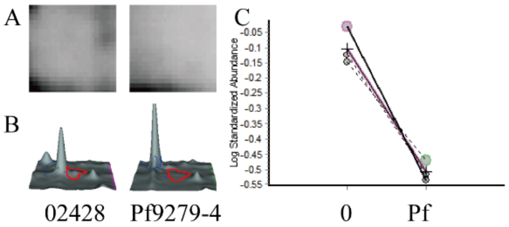

Supplementary Figure S9 | Temporal changes of DEPs after SBPH infestation at three time points (0 h, 6 h and 12h). Total leaf sheath proteins were extracted and separated by 2-DE. All the spots correspond to the DEPs in Supplementary Table S3. The center area of picture A represents differentially expressed spot. The left picture of A corresponds to spot derived from Figure 3 and the right picture of A corresponds to spot derived from Figure 4. The picture B represents three-dimensional diagram corresponding to picture A. The picture C was line chart of the differentially expressed spot.
